# Elucidation of the antipyretic and anti-inflammatory effect of 8-*O*-Acetyl Shanzhiside methyl ester based on intestinal flora and metabolomics analysis

**DOI:** 10.3389/fphar.2025.1482323

**Published:** 2025-04-28

**Authors:** Xiaolin Li, Tianlong Liu, Keke Liang, Renjie Wang, Jun Yang, Yidan Chen, Rong Wang, Maoxing Li

**Affiliations:** ^1^ College of Pharmacy, Gansu University of Chinese Medicine, Lanzhou, China; ^2^ Department of Pharmacy, The 940th Hospital of Joint Logistic Support Force of Chinese People’s Liberation Army, Gansu Plateau Pharmaceutical Technology Center, Lanzhou, China; ^3^ Academy of Military Medical, Academy of Military Sciences, Beijing, China; ^4^ National Key Laboratory of Kidney Diseases, Beijing, China

**Keywords:** 8-*O*-Acetyl Shanzhiside methyl ester, yeast-induced pyrexia in rats, intestinal flora, metabolomics, neurotransmitter

## Abstract

**Introduction:**

*Phlomoides rotata* (Benth. ex Hook.f.) Mathiesen (syn. *Lamiophlomis rotata* (Benth. ex Hook.f.) Kudô) (*P. rotate*) is a traditional Tibetan medicine known for its hemostatic, analgesic, and anti-inflammatory effects, as well as its high content of 8-*O*-Acetyl Shanzhiside methyl ester (8-*O*aS). Clinical and experimental studies have reported gastrointestinal side effects, such as diarrhea, loose stools, even to black stools, associated with *P. rotata*. Given the bitter taste characteristic, laxative and antipyretic effects of iridoid glycosides, this study aims to investigate the antipyretic and anti-inflammatory effects of 8-*O*aS (the primary iridoid glycosides of *P. rotate*) on yeast-induced pyrexia in rats. Additionally, the role 8-*O*aS in modulating the intestinal flora composition and metabolome profile is explored.

**Methods:**

The pyretic rat model was established by injected subcutaneously with 20% dry yeast suspension. Serum, hypothalamic tissues and colon content were collected for the assessment of relevant indicators. The peripheral inflammatory factors and central thermoregulatory mediators were assessed using enzyme-linked immunosorbent assay (ELISA). The expressions of mRNA and protein in hypothalamic tissue were evaluated through polymerase chain reaction (PCR), immunohistochemistry, and western blotting. 16S rDNA sequencing and LC-MS/MS were performed to determine the alteration and correlation of the intestinal flora and neurotransmitters in the colonic contents and hypothalamus.

**Results and discussion:**

Results show that 8-*O*aS treatment reduced pyrogenic cytokines (such as IL-6, IL-1β), and down-regulated the level of central thermoregulatory mediators (PGE_2_), via multiply involved in TLR4/NF-κB and HSP70/NF-κB signaling pathways. Crucially, 8-*O*aS treatment significantly reduced the relative abundance of *Alistipes *(*P* < 0.01), *Odoribacter* (*P* < 0.05) and *Alistipes_finegoldii* (*P* < 0.05) in the intestinal flora. The correlation analysis demonstrated that 8-*O*aS treatment significantly correlated with the increasing on the abundance of *Alistipes* and levels of 5-hydroxytryptamine (*P* < 0.01), and tryptamine (*P* < 0.01). Our findings indicate that 8-*O*aS exhibits significant antipyretic and anti-inflammatory properties, potentially mediated by intestinal flora and metabolites of neurotransmitters. The results of this study may help to elucidate the antipyretic and anti-inflammatory mechanism of 8-*O*aS based on intestinal flora and metabolomics analysis.

## Introduction

Fever represents a complex pathophysiological defense mechanism employed by the body in response to disease and foreign bacterial infections. It is a prevalent symptom associated with infectious and inflammatory conditions. The neurotransmitters such as acetylcholine (Ach), norepinephrine (NE), and serotonin (5-hydroxytryptamine/5-HT) play a significant role in thermoregulation (Voronova, 2021; Bo et al., 2019; Kesić et al., 2023). In recent years, a growing body of research has demonstrated that intestinal flora is involved in hypothalamic thermoregulatory processes by influencing the activation of neural signaling pathways and neurotransmitter expression through the gut-brain axis (Bo et al., 2019; Li S. et al., 2023; Mhanna et al., 2024; Loh et al., 2024). *Lactobacillus* spp. and *Bifidobacterium* spp. are capable of synthesizing γ-aminobutyric acid (GABA), while *Streptococcus* spp., *Candida* spp., and *Enterococcus* spp. have the ability to produce 5-HT (Eicher and Mohajeri, 2022). Neurotransmitters are unable to cross the blood-brain barrier (BBB) to act directly on the brain. Interestingly, certain amino acids act as precursor molecules for the synthesis of certain neurotransmitters possess the ability to traverse the BBB and influence brain function by modulating neurotransmitter-related metabolic pathways through interactions with intestinal flora. Phenylalanine can be metabolized to tyrosine, which subsequently leads to the production of neurotransmitters such as dopamine, DL-adrenaline, and NE. *Bifidobacterium* has been shown to elevate phenylalanine levels, while tryptophan acts as a precursor to the neurotransmitter 5-HT. Additionally, *Bifidobacterium infantis* is known to enhance circulating tryptophan levels (Eicher and Mohajeri, 2022). Therefore, intestinal flora influences the development of various diseases by regulating the expression of neurotransmitters within the nervous system through the gut-brain axis.

8-*O*-Acetyl Shanzhiside methyl ester (8-*O*aS), an iridoid glucoside, is isolated from the leaves of *P. rotata* (Benth. ex Hook.f.) Mathiesen (syn. *Lamiophlomis rotata* (Benth. ex Hook.f.) Kudô). *Phlomoides rotata* is a traditional Chinese medicinal plant utilized by the Tibet, Mongolian, and Naxi ethnic groups in China. The functions of this traditional Chinese medicine include promoting blood circulation to stop bleeding, expelling wind, and alleviating pain. And it is commonly utilized in traditional medicine to address issues such as dysmenorrhea due to blood stasis, metrorrhagia and metrostaxis, joint effusion of yellow, as well as injury of muscles and bones by falling and flashback deflate (Cui et al., 2020). Recent pharmacological researches have demonstrated that *P. rotata* possesses anti-inflammatory, antioxidant, antitumor, and anti-ulcer properties. It is frequently utilized in the management of postoperative bleeding, pain, arthritis, gingivitis, fractures, and various other conditions within domestic clinical practice. Its application prospects are extensive and promising. *Phlomoides rotata* has been formulated into various dosage forms such as granules, capsules, soft capsules, tablets, dispersible tablets, effervescent tablets, chewable tablets, and drop pills, which have been put on the market for use. Among them, Duyiwei tablets and Duyiwei capsules have been included in the Chinese Pharmacopoeia since 1995 and 2000, respectively. Iridoids, flavonoids and phenylethanoid glycosides are the main active components of *P. rotata*. Traditional Chinese medicine theory has established that the application of herbs with properties aimed at clearing heat and purgation, as well as bitter herbs, is an effective approach for treating heat syndrome within traditional Chinese medicine. Given the purgation properties of iridoid glycoside derived from *P. rotata*, our research group has further discovered that iridoid glycoside possess a beneficial effect on intestinal lubrication and promote bowel movements, which can shorten the defecation time of first black stool, increase the number of black stool, promote the intestinal propulsion rate, and alter the defecation situation in mice subjected to a constipation model induced by compound diphenol. The antipyretic and anti-inflammatory activity has been already observed for several iridoid glycosides, the most important is that of geniposide of *Gardenia jasminoides* Ellis as reported in the following work and the cited literature (Li et al., 2024).

8-*O*aS exhibits multiple pharmacological effects, including neuroprotective and analgesic properties. Its neuroprotective role following cerebral ischemia and reperfusion (I/R) injury is mediated through mechanisms that enhance angiogenesis (Jiang et al., 2011). Additionally, its protective effects in cerebral I/R injury are facilitated by the suppression of inflammatory processes (Zhang et al., 2014). It also provides protection against cognitive deficits and anxiety-like behaviors induced by sleep deprivation (Li Y. J. et al., 2023). However, there has yet to be any research that demonstrates whether 8-*O*aS possesses antipyretic and anti-inflammatory effects based on yeast-induced pyrexia model in rats. Therefore, given the bitter taste and laxative activity, a yeast-induced pyrexia model in rats was established to investigate whether the 8-*O*aS has antipyretic and anti-inflammatory effects.

This study aims to explore the antipyretic and anti-inflammatory effect of 8-*O*aS, focusing on the roles of intestinal flora and neurotransmitter metabolites. Our study first established a yeast-induced model pyrexia in rats, monitored the anal temperature of rats at various time points, and detected peripheral inflammatory factors as well as central thermoregulatory mediators. Furthermore, we sequenced the 16S rDNA in the intestinal flora and performed LC-MS/MS on metabolites. We then integratively analyzed their correlations to investigate the potential mechanisms underlying the antipyretic and anti-inflammatory effects of 8-OaS.

## Materials and methods

### Animals

A total of one hundred male sprague-dawley (SD) rats (weighing 180–220 g) were purchased from Ji’nan Pengyue Laboratory Animal Breeding Co., Ltd. (Jinan, China). The production license number is SCXK (LU) 2022-0006. They were fed adaptively under the conditions of temperature (20°C–25°C), relative humidity 40%–50%, and alternating light (12 h light/dark cycle) with unrestricted free access to sufficient food and water. The animals were allowed to habituate to the housing facilities for at least 1 week before the experiment began. All animal experiments were approved by the Animal Ethics Committee of the 940th Hospital (No. 2024KYLL145) and performed according to the Guidelines for the Care and Use of Laboratory Animals.

### Reagents and chemicals

8-*O*aS(purity ≥ 98%; batch number: AZCC2903) was purchased from Chengdu Alfa Biotechnology Co., Ltd. (Chengdu, China); highly active dry yeast was purchased from Angel yeast Co., Ltd. (Yichang, China). Aspirin enteric-coated tablets was purchased from Hunan Xinhui Pharmacy Co., Ltd. (Changsha, China); prostaglandin E_2_ (PGE_2_) rat assay kit, tumour necrosis factor-α (TNF-α) assay kit, NF-κB test Kit, interleukin-6 (IL-6) assay kit, interleukin-1β (IL-1β) assay kit, arginine vasopressin (AVP) assay kit, 5-HT test kit, macrophage inflammatory protein-1α (MIP-1α) assay kit, interferon-γ (IFN-γ) assay kit, corticotropin-releasing hormone (CRH/CRF) and substance P (SP) assay kit were purchased from Nanjing Jiancheng Bioengineering institute (Nanjing, China). Anti-TLR4 rabbit pAb, anti-phospho-NF-κB p65 (S536) rabbit pAb, anti- TNF-α rabbit pAb, and anti-COX2/Cyclooxygenase 2 rabbit pAb were purchased from Wuhan Servicebio Technology Co., Ltd. (Wuhan, China). HSP70 antibody was purchased from Affinity Biosciences (Liyang, China).

### Experimental animals and design

Three days prior to the formal experiment, one hundred SD male rats underwent adaptive measurements three times daily, as well as two additional measurements conducted before the establishment of the fever model, with a 1-h interval between each measurement. The rats were excluded if they exhibited a single temperature higher than 38°C or a temperature difference greater than 0.5°C between the two temperatures, and the average temperature of the two temperatures was taken as the basal body temperature of the rat.

The body temperatures of rats were measured using a calibrated medical thermometer. Prior to measurement, the temperature probe was prepared by establishing a physical limit marker at 3 cm from its tip to standardize insertion depth. A thin layer of medical-grade Vaseline was uniformly applied to the distal 3 cm of the probe to ensure adequate lubrication and prevent rectal tissue damage. During the procedure, each rat was humanely restrained using a dedicated rodent immobilization device. The lubricated probe was then gently inserted rectally until the pre-marked 3 cm depth was reached. The thermometer was activated, and temperature readings were automatically recorded upon completion of the device’s audible notification signal. Three consecutive measurements were obtained at 2-min intervals for each subject, with the probe thoroughly disinfected between animals using 70% ethanol.

Following the screening of body temperature, the seventy-two rats were randomly divided into six groups (n = 12): normal control group (NG, sterilized water for injection, 10 mL/kg), fever model group (MG, sterilized water for injection, 10 mL/kg); 8-*O*aS high dose group (8-*O*AS-H, 200 mg/kg); 8-*O*aS medium dose group (8-*O*AS-M, 150 mg/kg); 8-*O*aS low-dose group (8-*O*aS-L, 100 mg/kg); positive control group (PG, aspirin, 100 mg/kg). The dosage of 8-*O*aS is obtained based on preliminary experiments. Except for the NG, all other groups were injected subcutaneously with 20% dry yeast suspension to establish the rat fever model (Li et al., 2024; Sherif et al., 2024). After a subcutaneous injection of dry yeast for 5 h, the rats were screened out and subsequently administered corresponding drugs; the body temperature was measured continuously for 7 h following drug administration, with an interval of 1 h between each measurement. At the end of the temperature measurements, the maximum temperature rise △t at each time point was calculated (△t = body temperature at each time point - basal body temperature).

At the conclusion of temperature measurement, blood was collected from the abdominal aorta using a negative pressure tube. A volume of 5 mL was extracted from each rat and subsequently centrifuged at 2,200 *g* for 10 min. The supernatant was aspirated, aliquoted and stored in a refrigerator at −80°C. Additionally, the hypothalamic tissues and colonic contents were removed and stored at −80°C.

### Enzyme-linked immunosorbent assay (ELISA)

ELISA was conducted to assess the levels of IL-1β, IL-6, TNF-α, IFN-γ, PGE_2_, MIP-1α, and other corresponding indicators in blood serum, according to the manufacturer’s protocol (Zhang et al., 2019). The sample (50 μL) or standard (50 μL) was added to the enzyme-labeled plates that have been pre-coated with antibodies, then biotin-labeled recognition antigen was added and incubated at 37°C for 30 min. After washing five times by PBST, 50 μL of HRP-conjugated avidin was added and incubated at 37°C for 30 min. After washing the plate, the plate was developed with 50 μL of TMB substrate for 15 min in the dark. The reaction was stopped with 2 M H_2_SO_4_ and optical density was measured at a wavelength of 450 nm using a spectrophotometer.

### Real-time PCR

The RNA of hypothalamic tissue was extracted by the trizol method and reverse transcribed to cDNA using a reverse transcription kit. Primers were designed according to gene sequences (Ping et al., 2020), NF-κB: upstream GCT​GAT​GGA​GTA​CCC​TGA​AGC, downstream ATG​TCC​GCA​ATG​GAG​GAG​AAG; TNF-α: upstream: CCA​CGC​TCT​TCT​GTC​TAC​TG, downstream GCT​ACG​GGG​CTT​GTC​ACT​C; IL-6: upstream GTT​GCC​TTC​TTG​GGG​ACT​GAT​GT, downstream ATA​CTG​GGT​CTG​TTG​GGG​TGG​GT,; IL-1β: upstream GCT​TCA​AAT​CTC​ACA​GCA​GCA​T, downstream TAG​CAG​GTC​GTC​ATC​ATC​CCA​C; HSP70: Upstream CGA​GGA​GGT​GGA​TTA​GAG​GC, downstream GCT​GAG​GTG​TTC​GCA​GGA​A; TRPV4: Upstream AAG​TGG​CGT​AAG​TTC​GGG, downstream CGTGGTACGGTAAGGGT; GAPDH: Upstream ACA​GCA​ACA​GGG​TGG​TGG​AC, downstream TTT​GAG​GGT​GCA​GCG​AAC​TT. The amplification reaction of the GAPDH and the target gene was performed simultaneously for each batch of samples.

### Western blotting

The total protein in the hypothalamic tissue was extracted utilizing a high-efficiency RIPA lysis buffer, which was supplemented with protease inhibitor and phosphatase inhibitor. The protein concentration was determined by the BCA protein assay kit. The protein samples were separated by 10% SDS-PAGE, run at 80 V for 40 min and then at 110 V for 80 min, before being transferred to a polyvinylidene fluoride (PVDF) membrane. After the transmembrane process, the PVDF membrane was removed and immersed in 1×TBST containing 5% skim milk powder. This mixture was sealed at room temperature for 2 h, followed by overnight incubation with primary antibodies at 4°C. The primary antibodies used in this study were against TLR4 (1:1,000), TNF-α (1:1,000), p-NF-κB p65 (1:1,000), p-IκB-α (1:1,000), HSP70 (1:1,000), COX2 (1:1,000), and GAPDH (1:1,000). After primary antibody incubation, wash with 1×TBST at room temperature, allowing for 8 min of washing each time. Subsequently, the PVDF membrane was immersed into the secondary antibody solution (1:5,000) and incubated at room temperature for 2 h. ImageJ software was utilized for the purpose of quantitative analysis (Wang et al., 2024; Hou et al., 2024).

### Immunohistochemical staining

Hypothalamic tissues were paraffin sectioned and deparaffinized for hydration, antigen repair, and closure. Then it was incubated with 3% endogenous H_2_O_2_ for 10 min. Then, the tissue sections were incubated with primary antibodies against IL-1β (1:500) and IL-6 (1:500) overnight. On the following day, secondary antibody was added and reacted for another 10 min, and stained with diaminobenzidine for 1 min. Subsequently, the sections were counterstained with hematoxylin, dehydrated, and sealed for observation using a microscope. The whole positive rate was analyzed using ImageJ software.

### Intestinal flora analysis

Microbial DNA was extracted utilizing the HiPure Soil DNA Extraction Kit (Magen, China) in accordance with the provided instructions. Subsequently, PCR sequencing was conducted. The amplification of the target region of the 16 S rDNA gene via PCR was performed under the following conditions: 95°C for 5 min, followed by 30 cycles of 95°C for 1 min, 60°C for 1 min, and 72°C for 1 min, and finally 72°C for 7 min using the primers (sequences upstream: CCTACGGGGNGGCWGCAG; downstream: GGACTACHVGGGTATCTAAT). Amplification system: 50 μL mixture containing 10 µL 5×Q5@ Reaction Buffer, 10 µL 5×Q5@ High GC Enhancer, 1.5 µL 2.5 mM dNTPs, 1.5 µL upstream and downstream primers (10 µM), 0.2 µL Q5@ High-Fidelity DNA Polymerase and 50 ng of template DNA. Finally, Illumina sequencing was applied. Amplification product quality was assessed using a 2% agarose gel, PCR product purification was performed using AMPure XP Beads (Beckman, United States) and quantification was conducted using Qubit 3.0. Sequencing libraries were prepared using the Illumina DNA Prep Kit (Illumina, United States). Library quality assessment was performed utilizing the ABI StepOnePlus Real-Time PCR System (Life Technologies, United States). Qualified libraries were up-sequenced using PE250 mode pooling on Novaseq 6000.

After sequencing to obtain raw reads, the initial step involves filtering out low-quality reads, followed by their subsequent assembly. The double-ended reads are spliced into tags. The tags are subsequently filtered and the resulting dataset is referred to as a clean tag. Subsequently, clustering is performed based on a clean tag to eliminate the chimeric tags detected during the cluster comparison process. The final output obtained from this procedure is referred to the as effective tag. After obtaining OTUs, OTU abundance statistics were performed based on the effective tag. The following analyses were performed: species annotation, alpha diversity analysis, beta diversity analysis, species composition analysis, and differential species screening.

### Metabolomics analysis

Appropriate samples of hypothalamic tissue and colon contents were carefully placed in 2 mL centrifuge tubes. A total of 500 μL of an 80% methanol-water solution (containing 0.1% formic acid) was added to the tube. The mixture was vortexed for 1 min and subsequently ground for 5 min; after centrifugation, the supernatant was collected for LC-MS/MS analysis.

The liquid chromatography system utilized was a Waters Acquity UPLC, coupled with a mass spectrometer from AB SCIEX 5500 QQQ-MS. The chromatographic columns employed in this study were Acquity UPLC BEH C18 (1.7 µm, 2.1 mm × 100 mm) and Acquity UPLC HSS T3 (1.8 µm, 2.1 mm × 100 mm). The chromatographic separation was conducted under conditions that included a column temperature of 40°C and a flow rate of 0.20 mL/min. The composition of the mobile phase comprised two components: component A, which was water containing 0.01% formic acid, and component B, which was acetonitrile (0.01% formic acid). The total run time was 10 min, with an injection volume of 6 µL. The gradient eluting conditions for samples were as follows: 0–3 min, 95%–55% A; 3–13 min, 55%–5% A; 13–14 min, 5% A; the UHPLC elution conditions for urine samples were as follows: 0–1 min, 99% A; 1–7 min, 99%–60% A; 7–8 min, 60% A; 8–9 min, 60%–99% A; 9–10 min, 99%A. The mass spectrometric conditions were as follows: ion source, ESI; curtain gas, 35 arb; collision gas, 9 arb; ion spray voltage, 4,500 V; ion source temperature, 550°C; ion source gases, 55 arb each for Ion Source Gas1 and IonSource Gas2. The parameters for the MRM acquisition were established based on the aforementioned chromatographic and mass spectrometric conditions. Subsequently, standard solution preparations were injected into the sample vials for analysis.

Preparation of standard solutions is outlined as follows: a 100 μg/mL standard solution was prepared using 80% methanol containing 0.1% formic acid. Subsequently, a series of standard solutions with appropriate concentrations (0.24, 0.49, 0.98, 1.95, 3.91, 7.81, 15.63, 31.25, 62.5, 125, 250, and 500 ng/mL) was subsequently prepared through further dilution.

The standard curve was plotted according to the peak areas of the standards at various concentrations and their corresponding concentrations. The *R* ^2^ was exceeded 0.99, which indicated that the standard curve had a superior linear relationship and could be used for the calculating the concentrations of the neurotransmitters in the samples. Finally, the integration was carried out using MultiQuant software and quantitative analyses were performed using standard curves.

### Data analysis

Statistical analyses were performed using SPSS 25.0 and GraphPad Prism 8.0 software. All data was presented as mean ± standard deviation ( $\bar{x}$ ±s). Unless otherwise stated, statistical comparisons were performed using a single-factor analysis of variance (ANOVA) followed by the LSD test. The p-value < 0.05 indicated statistical significance. The pearson correlation coefficient was utilized in the correlation analysis between the intestinal flora and metabolites. Metabolomic profiling was performed in collaboration with Gene *Denovo* Biotechnology Co., Ltd. (Guangzhou, China) including mass spectrometry analyses and/or bioinformatics analysis.

## Results

### Antipyretic and anti-inflammatory effects of 8-*O*aS in yeast-induced pyrexia in rats

**FIGURE 1:**
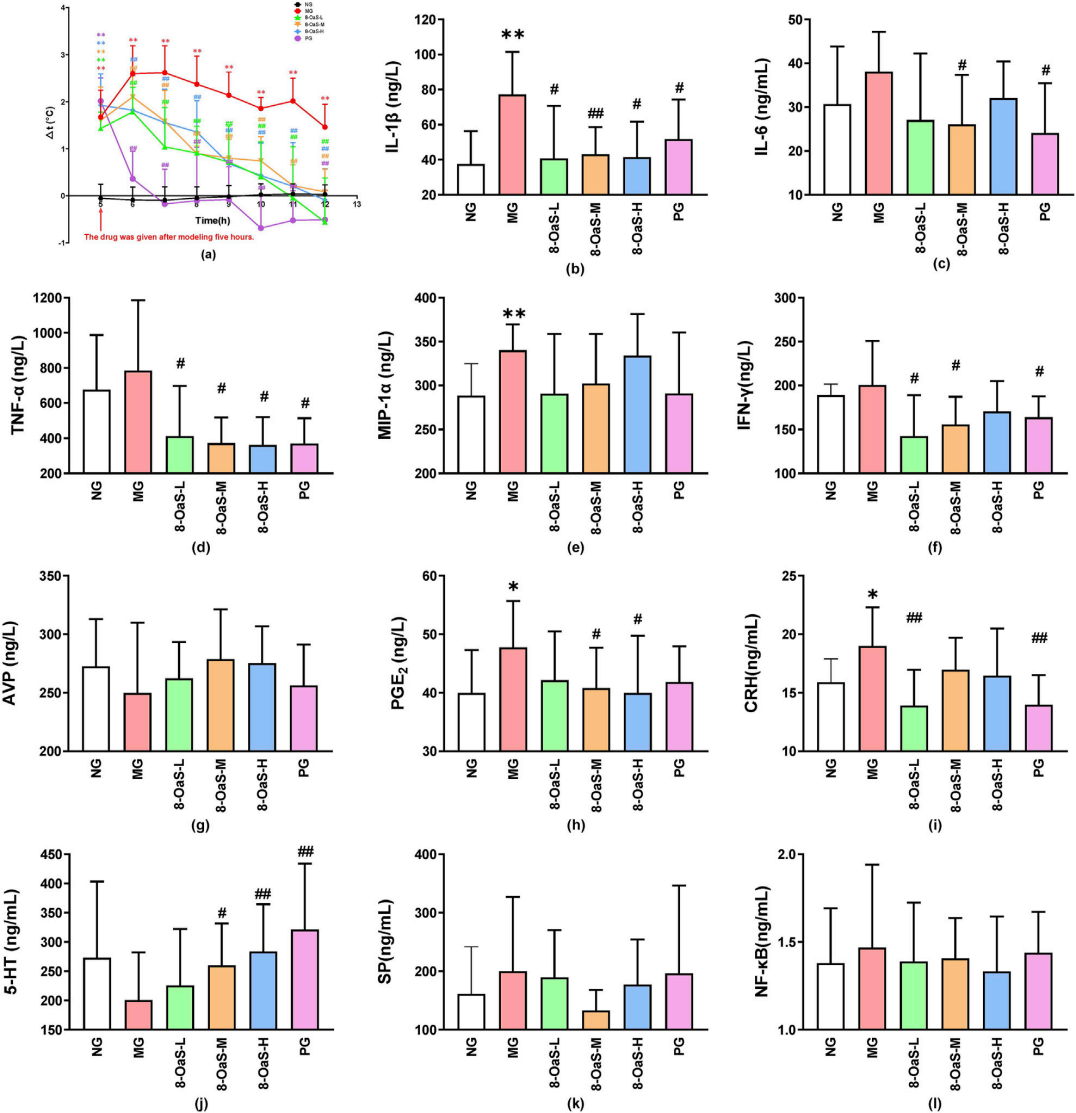
The antipyretic and anti-inflammatory effects of 8-*O*aS in yeast-induced pyrexic rats. **(a)** Temperature change, **(b)** IL-1β, **(c)** IL-6, **(d)** TNF-α, **(e)** MIP-1α, **(f)** IFN-γ, **(g)** AVP, **(h)** PGE_2_, **(i) **CRH **(j)** 5-HT **(k)** SP, and **(l)** NF-κB (n = 12). Note: Compared with the control group, *P < 0.05, **P < 0.01; compared with the fever model group, ^#^ *P* < 0.05, ^##^ *P* < 0.01.

The variations in temperature observed within 7 h post-administration are illustrated in Figure 1a. Compared with the control group, the remaining five groups exhibited a significant increase in body temperature after 5 h of modeling (*P* < 0.01), indicating that the yeast-induced pyrexia model in rats was successfully established. Compared with the fever model group, various dosage groups of 8-*O*aS demonstrated an antipyretic effect.

Serum cytokines, thermoregulatory mediators, neurotransmitters were closely associated to the degree of fever (Figures 1b–l). ELISA was employed to assess the alterations in cytokine levels within serum. The concentrations of IL-1β, IL-6, TNF-α, MIP-1α, IFN-γ, PGE_2_, CRH, SP, and NF-κB in the MG were found to be elevated. Conversely, the serum AVP, 5-HT of the MG was decreased. The above results indicated that the yeast-induced pyrexia model in rats was successfully established. After the administration of 8-*O*aS, the above indicators tended to recover to the normal levels.

**FIGURE 2:**
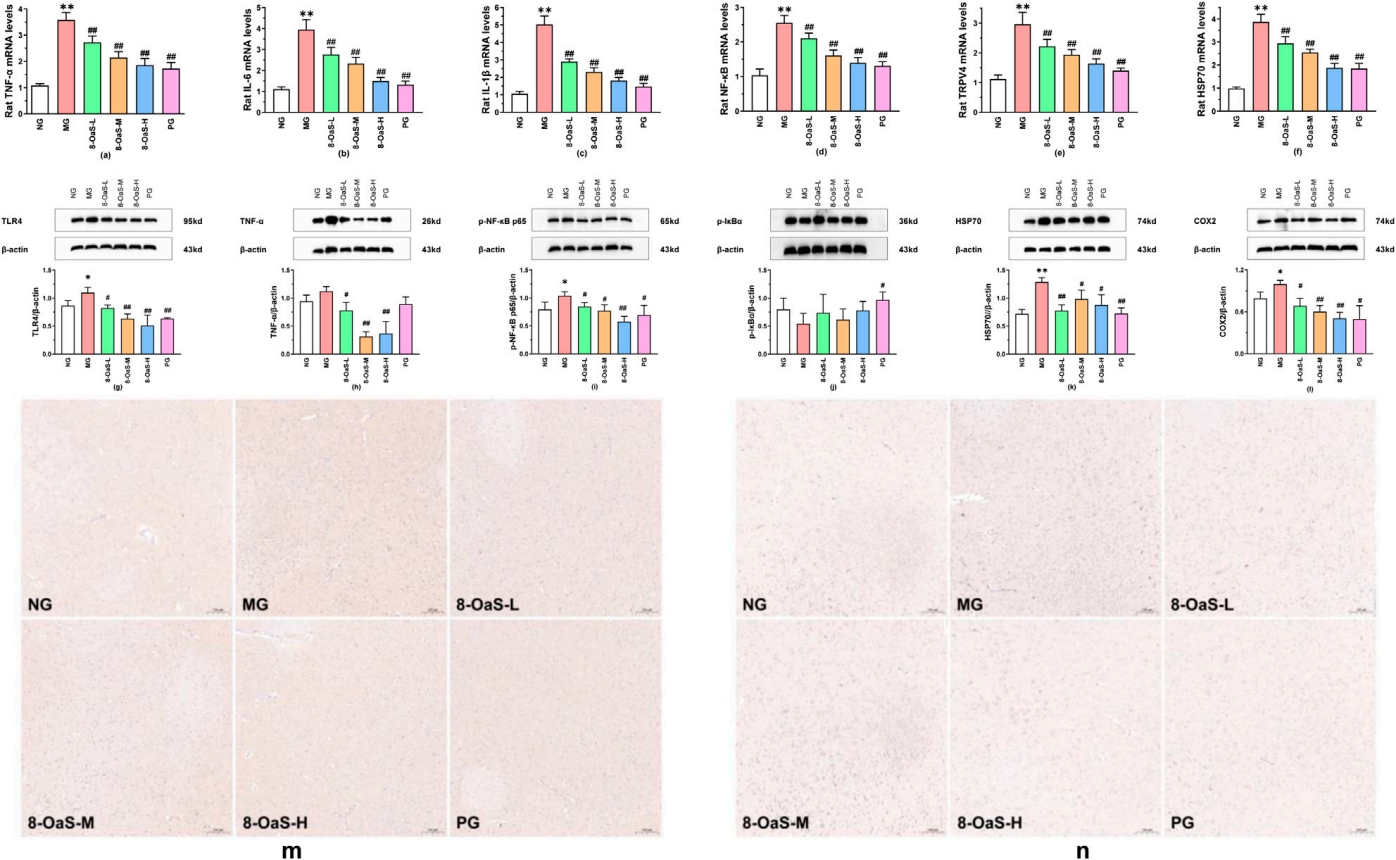
Effects of 8-*O*aS on the mRNA and proteins in yeast-induced pyrexic rats. **(a-f)**: Effects of 8-*O*aS on the mRNA expression levels of TNF-α **(a)**, IL-6 **(b)**, IL-1β **(c)**, NF-κB **(d)**, TRPV4 **(e)**, and HSP70 **(f)** in the hypothalamus of yeast-induced pyrexic rats. **(g-l)** Effects of 8-*O*aS on the proteins expression levels of TLR4 **(g)**, TNF-α **(h)**, p-NF-κB p65 **(i)**, p-Iκ Bα **(j)**, HSP70 **(k)**, and COX2 **(l)** expression levels in the hypothalamus of yeast-induced pyrexia in rats. **(m,n)** Representative immunohistochemical staining results of IL-1β and IL-6 proteins in the hypothalamus (100 μm). Note: Compared with the normal control group, *P < 0.05, **P < 0.01; compared with the fever model group, ^#^ *P*< 0.05, ^##^ *P* < 0.01.

The mRNA expression of the hypothalamus was detected by PCR. The findings are presented in Figures 2a–f. Compared with the NG, the mRNA expressions of TNF-α, IL-6, IL-1β, NF-κB, TRPV4, and HSP70 in the MG were significantly upregulated (*P* < 0.01), indicating that the expression of mRNA was enhanced under fever conditions. Compared with the MG, the mRNA expressions of TNF-α, IL-6, IL-1β, NF-κB, TRPV4, and HSP70 in the 8-*O*aS dose groups and the PG were significantly downregulated (*P* < 0.01).

Western blotting method was used to detect the expression of proteins in the rat hypothalamus. The results are shown in Figures 2g–l. Compared with the NG, the protein expressions of TLR4 (*P* < 0.05), TNF-α, p-NF-κB p65 (*P* < 0.05), HSP70 (*P* < 0.01), COX2 (*P* < 0.05) were upregulated and the protein expression of p-IκBα was downregulated in the MG. Compared with the MG, the protein expressions of TLR4 (*P* < 0.05), TNF-α (*P* < 0.05), p-NF-κB p65 (*P* < 0.05), HSP70 (*P* < 0.01), and COX2 (*P* < 0.05) were significantly decreased in the 8-*O*aS-L, while the protein expression of p-IκBα was increased. The protein expressions of TLR4 (*P* < 0.01), TNF-α (*P* < 0.01), p-NF-κB p65 (*P* < 0.05), HSP70 (*P* < 0.01), and COX2 (*P* < 0.05) in the 8-*O*aS-M were significantly elevated, but the protein expression of p-IκBα was reduced. The protein expressions of TLR4 (*P* < 0.01), TNF-α (P < 0.01), p-NF-κB p65 (*P* < 0.01), HSP70 (*P* < 0.01), and COX2 (*P* < 0.05) were significantly downregulated and the protein expression of p-IκBα was upregulated in the 8-*O*aS-H. In the PG, the protein expression of TLR4 (*P* < 0.01), TNF-α, p-NF-κB p65 (*P* < 0.05), COX2 (*P* < 0.05) declined and the protein expression of p-IκBα significantly augmented (*P* < 0.05).

Immunohistochemical staining of IL-1β and IL-6 expressions in the hypothalamus revealed a significant increase in both IL-1β and IL-6 significantly increased in the MG (Figures 2m, n). Meanwhile, a marked decreased in the expressions of IL-1β and IL-6 were observed in the 8-*O*aS dose groups.

### Changes in the intestinal flora of yeast-induced pyrexia in rats and 8-*O*aS alters the composition of intestinal flora

**FIGURE 3:**
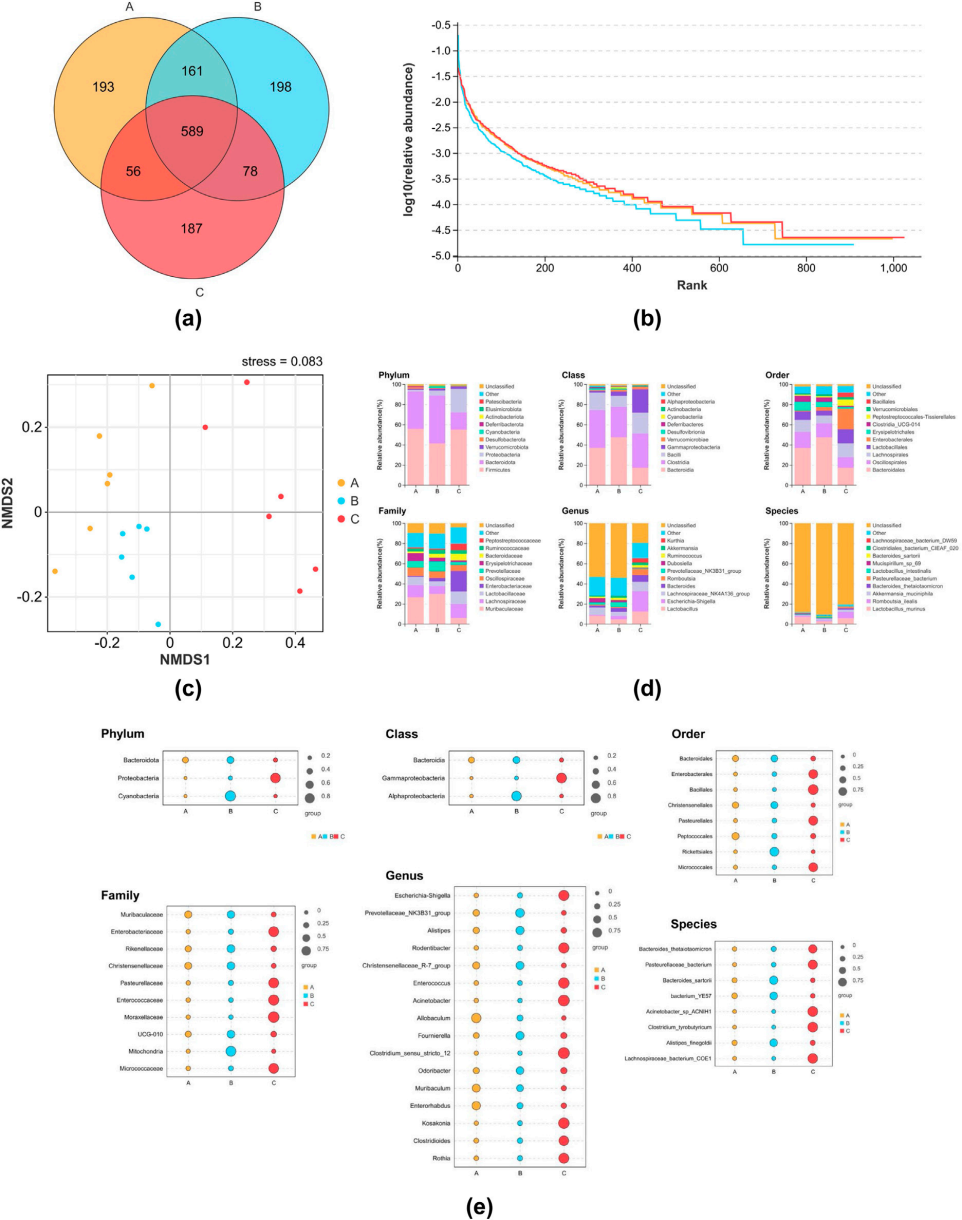
The effect of 8-*O*aS on the intestinal flora. **(a)** OUT Veen plots for each group; **(b)** Rank Abundance curves; **(c)** NMDS analysis based on OUT; **(d)** bar charts for analysis of species composition at different levels (phylum, class, order, family, genus, species); **(e)** bubble charts for screening and analysis of indicator species at different levels (phylum, class, order, family, genus, species). n = 6. Note: A: normal control group; B: fever model group; C: 8-*O*aS high dose group.

The Veen diagram presented in Figure 3a illustrates that there were 999 OTUs in the NG, with 193 of these being unique. Additionally, there were 1,026 OTUs in the MG, with 198 of these being unique, and 910 OTUs in the 8-*O*aS-H, with 187 of these being unique. The taxonomic richness and homogeneity of a sample are visualized by rank abundance curves (Figure 3b). As the number of test samples increases, the curves gradually flatten, evenly distributed and concentrated. With sufficient sampling, it can be used for subsequent data analysis. The NMDS analysis revealed significant structural differences among the three groups (Figure 3c). As shown by the β diversity Adonis grouping test (Table 1) and the Anosim grouping test (Table 2), the composition of the rat intestinal flora in the MG differed significantly from that of the NG and 8-*O*aS-H group.

**TABLE 1:** Adonis grouping test.

Diffs	D_f_	Sums of Sqs	Mean Sqs	F_value_	*R* ^2^	*P*_value_	Significant
A-vs.-B	1	0.3104	0.3104	2.1852	0.1793	0.004	**
B-vs.-C	1	0.7156	0.7156	4.7992	0.3243	0.004	**
A-vs.-B-vs.-C	2	1.1798	0.5899	3.8103	0.3369	0.001	**

Note: **: *P* < 0.01. A: normal control group; B: fever model group; C: 8-*O*aS high dose group. Diffs, difference comparison group; Df, degrees of freedom; Sums of Sqs, total variance, also known as the sum of squares of deviations; Mean Sqs, mean square (difference), that is, Sums of Sqs/Df; F _value_, F-test value; *R* ^2^, indicates that different subgroups of the sample differences in the degree of explanation, that is, subgroups of the variance of the ratio of variance to the total variance, the greater the *R* ^2^ indicates that the subgroups of the differences in the higher degree of explanation; P _value_, less than 0.05 indicates that this test is highly credible; significant: indicates whether significant differences; **: indicates that the significant differences.

**TABLE 2:** Anosim grouping test.

Diffs	R_value_	*P*_value_	Significant
A-vs.-B	0.3778	0.006	**
B-vs.-C	0.8167	0.002	**
A-vs.-B-vs.-C	0.6354	0.001	**

Note: **: *P* < 0.01. A: normal control group; B: fever model group; C: 8-*O*aS high dose group. Diffs, difference comparison group; Pvalue: P-value, less than 0.05 indicates that the confidence of this test is high; significant: indicates whether there is a significant difference.

The top ten species from each group of rat intestinal flora objects, ranked according to their constitutive abundance at the taxonomic phylum, class, order, family, genus, species level, are shown in Figure 3d. After conducting fever modeling in rats and administering drug, the proportions of the top ten species exhibited distinct changes.

The analysis of indicators calculates the indicator value of every species for each subgroup, based on the abundance and frequency of occurrence of the species in the sample. A higher value indicates that the species is more likely to serve as an indicator species for that subgroup. At the phylum level (Figure 3e), Bacteroidota, Proteobacteria, and Cyanobacteria in the three comparison groups were screened as indicator species. Welch’s t-test found that compared with the NG, the relative abundance of Cyanobacteria in the MG was significantly higher (*P* < 0.05), while the relative abundance of Cyanobacteria in the 8-*O*aS group was significantly lower (*P* < 0.05). At the genus level (Figure 3e), *Escherichia-Shigella*, *Prevotellaceae_NK3B31_group*, *Alistipes*, *Rodentibacter*, *Christensenellaceae_R-7_group*, *Enterococcus*, *Acinetobacter*, *Allobaculum*, *Fournierella*, *Clostridium_sensu_stricto_12*, *Odoribacter*, *Muribaculum*, *Enterorhabdus*, *Kosakonia*, *Clostridioides*, *Rothia* were screened as indicator species. Welch’s t-test showed that compared with the NG, the relative abundances of *Alistipes* and *Odoribacter* in the MG were significantly increased (*P* < 0.01). Compared with the MG, the relative abundances of *Alistipes* (*P* < 0.01) and *Odoribacter* (*P* < 0.05) in the 8-*O*aS-H were significantly decreased. *Bacteroides_thetaiotaomicron*, Pasteurellaceae*_bacterium*, *Bacteroides_sartorii*, *bacterium_YE57*, *Acinetobacter_sp_ ACNIH1*, *Clostridium_tyrobutyricum*, *Alistipes_finegoldii*, Lachnospiraceae*_bacterium_COE1* as indicator species screened at species level. Welch’s t-test showed that the relative abundance of *Alistipes_finegoldii* in the MG was significantly higher than that in the NG (*P* < 0.05). Compared with the MG, the relative abundance of *Alistipes_finegoldii* in the 8-*O*aS-H was significantly decreased (*P* < 0.05).

**FIGURE 4:**
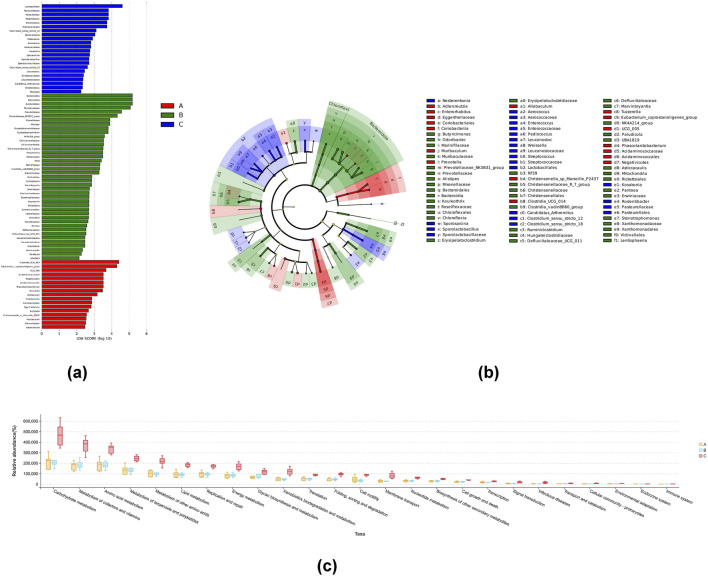
LEfSe analysis and the differences in metabolic pathways among different groups. LDA distribution of LEfSe results **(a)** based on the classification information in the genus level andthe taxonomic cladogram generated by LEfSe analysis **(b)**. The threshold value of the LDA score was set to 2, and an LDA score >2 was considered significant. n = 6. **(c)** Microbial metabolic function of intestinal flora predicted by PICRUSt2. Note: A: normal control group; B: fever model group; C: 8-*O*aS high dose group.

The diversity of intestinal flora was examined using the multi-level species hierarchy of LEfSe at genus level. The cladogram and LDA histogram (Figures 4a, b) indicated that 80 species exhibited differences in their relative abundances among the three groups, including 17, 41, and 22 species belonging to the NG, MG, and 8-*O*aS-H, respectively. The relative abundances of *Clostridia_UCG_014*, *Eubacterium_coprostanoligenes_group*, *UCG_005*, *Acidaminococcaceae*, *Negativicutes*, *Acidaminococcales*, *Phascolarctobacterium*, *Prevotella* and *Allobaculum* were abundant in the NG, while those of Bacteroidota, *Bacteroidia*, *Bacteroidales*, *Muribaculaceae*, *Prevotellaceae*, *Prevotellaceae_NK3B31_group*, *Rikenellaceae*, *Alistipes*, *Erysipelatoclostridiaceae*, *Erysipelatoclostridium*, *NK4A214_group*, *Christensenellaceae*, *Christensenellales*, *Christensenellaceae_R_7_group*, *Mitochondria*, *Rickettsiales*, *RF39*, *Marinifilaceae*, *Clostridia_vadinBB60_group*, and *Butyricimonas* were abundant in the MG. Moreover, the relative abundances of *Lactobacillales*, Pasteurellaceae, *Pasteurellales*, *Rodentibacter*, *Enterococcus*, *Enterococcaceae*, *Clostridium_sensu_stricto_12* and *Nesterenkonia* were enriched in the 8-*O*aS-H. To explore the potential effects of 8-*O*aS intervention on metabolic pathways of intestinal microflora in rats induced with yeast, we conducted PICRUSt 2 analysis based on the KEGG database (Figure 4c). In contrast to rats of the MG, multiple microbial metabolic pathways were significantly changed in 8-*O*aS-fed rats (8-*O*aS-H), which were mainly characterized by the regulation of carbohydrate metabolism, metabolism of cofactors and vitamins, amino acid metabolism, metabolism of terpenoids and polyketides, metabolism of other amino acids and so on.

### Utilizing metabolomic biomarkers in hypothalamic tissues as pharmacodynamic surrogate indices to elucidate the antipyretic and anti-inflammatory effects of 8-*O*aS

**FIGURE 5:**
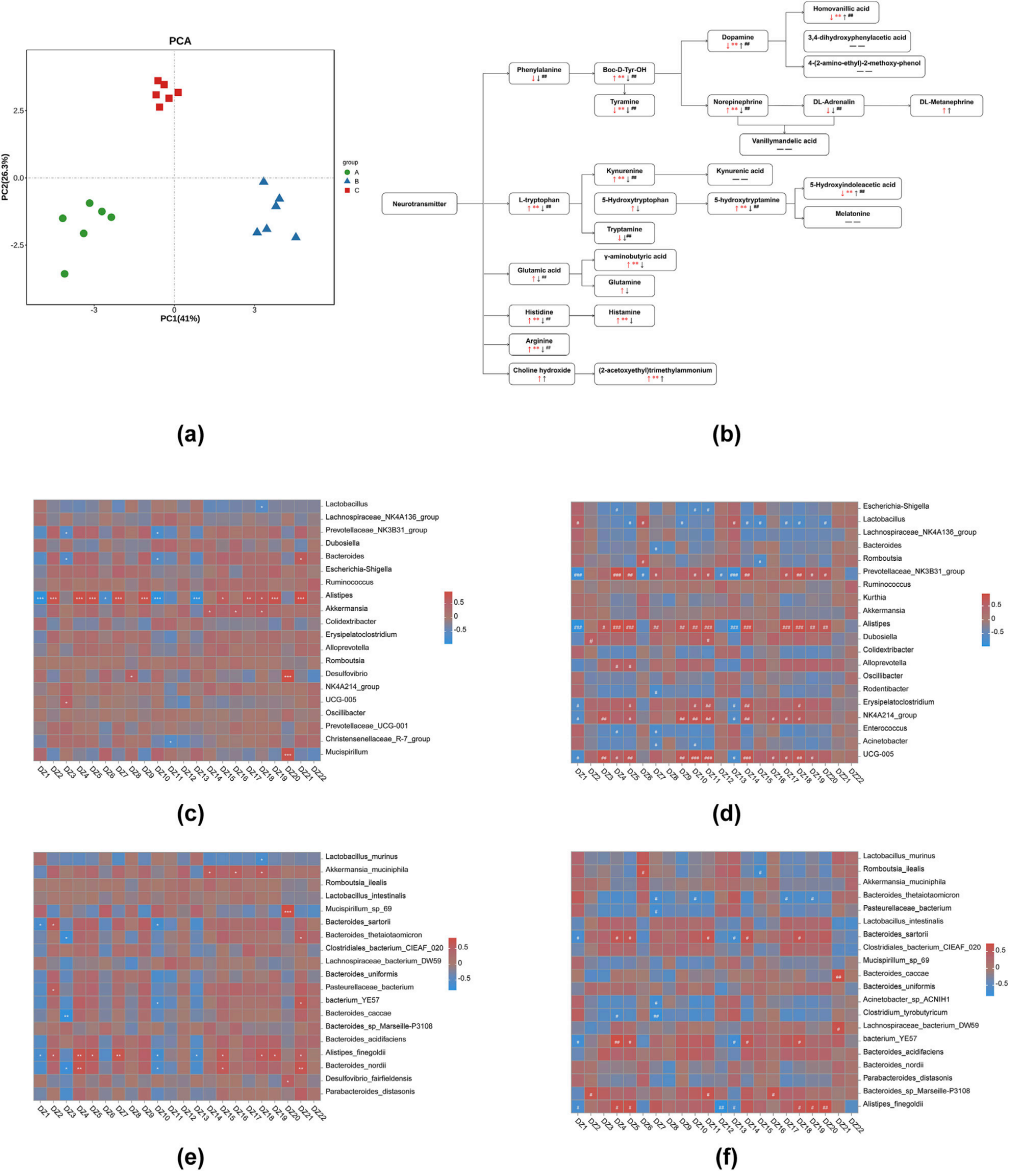
Metabolic analysis of potential biomarkers in hypothalamic tissues. **(a)** PCA analysis; **(b)** the significant difference of 8-*O*aS on neurotransmitter metabolism in hypothalamic tissue of yeast-induced pyrexia in rats (Ma et al., 2021) (Note: ↑: represents increase; ↓: represents decrease; arrows are red when the fever model group is compared with the control group; arrows are black when 8-*O*aS-H is compared with the fever model group. — —: indicates that the device is not detected.); **(c)** the heatmap of correlation analysis at genus level between NG and MG; **(d)** the heatmap of correlation analysis at genus level between MG and 8-*O*aS-H; **(e)** the heatmap of correlation analysis at species level between NG and MG; **(f)** the heatmap of correlation analysis at species level between MG and 8-*O*aS-H. (DZ1: 5-Hydroxyindoleacetic acid (5-HIAA); DZ2: Histamine; DZ3: DL-Adrenaline; DZ4: Norepinephrine (NE); DZ5: 5-hydroxytryptamine (Seronin; 5-HT); DZ6: Dopamine; DZ7: Histidine; DZ8: 5-Hydroxytryptophan (HTP); DZ9: Kynurenine (KYN); DZ10: Tyramine; DZ11: Tryptamine; DZ12: DL-METANEPHRINE; DZ13: Homovanillic acid; DZ14: Glutamic acid; DZ15: γ-Aminobutyric acid (GABA); DZ16: Glutamine; DZ17: Boc-D-Tyr-OH(Tyrosine); DZ18: Arginine; DZ19: L-tryptophan; DZ20: Phenylalanine; DZ21: (2-acetoxyethyl)trimethylammonium; DZ22: Choline hydroxide.). Note: Compared with control group, *P < 0.05, **P < 0.01, ***P < 0.01; Compared with the fever model group, ^#^ *P* < 0.05, ^##^ *P* < 0.01, ^###^ *P* < 0.01. A: normal control group (NG); B: fever model group (MG); C: 8-*O*aS high dose group (8-*O*aS-H).

Neurotransmitter metabolites and its variation trends in hypothalamic tissues were showed in Figure 5; Table 3. In hypothalamic tissue, neurotransmitters of the phenylalanine metabolic pathway are impacted. Compared with NG, the contents of phenylalanine and DL-adrenaline were found to be reduced in MG; however, these differences did not reach statistical significance. The contents of tyramine, dopamine and homovanillic acid were significantly decreased (*P* < 0.01). The levels of tyrosine and NE were significantly increased (*P* < 0.01), and the levels of DL-metanephrine were increased but the differences were not statistically significant. Compared with MG, the contents of phenylalanine, tyrosine, tyramine, DL-adrenaline and NE in 8-*O*aS-H were significantly decreased (*P* < 0.01), the contents of dopamine and homovanillic acid were significantly increased (*P* < 0.01), and the content of DL-metanephrine was increased but the difference was not statistically significant. In the neurotransmitters of L-tryptophan metabolic pathway, compared with the NG, the levels of L-tryptophan, KYN and 5-HT were significantly increased in the MG (*P* < 0.01). In contrast, while the level of 5-HTP was increased, the differences observed were not statistically significant. The contents of 5-HIAA (*P* < 0.01) and tryptamine were decreased. Compared with MG, the contents of L-tryptophan, KYN, 5-HT and tryptamine in 8-*O*aS-H were significantly decreased (*P* < 0.01), while the contents of 5-HTP were decreased but the difference was not statistically significant. The contents of 5-HIAA was significantly increased. In the neurotransmitters of glutamate metabolic pathway, the contents of glutamate, GABA and glutamine in MG were increased compared with NG, and the difference of GABA content was statistically significant (*P* < 0.01). Compared with the MG, the contents of glutamic acid (*P* < 0.01), GABA and glutamine in the 8-*O*aS-H were decreased. In the neurotransmitters of histidine metabolic pathway, the contents of histidine (*P* < 0.01) and histamine (*P* < 0.01) in the MG were significantly elevated compared with NG. Compared with MG, the content of histidine (*P* < 0.01) and histamine decreased in the 8-OaS-H. In the neurotransmitter of arginine metabolic pathway, the level of arginine in MG was significantly increased compared with NG (*P* < 0.01). Compared with MG, the level of arginine in 8-*O*aS-H was significantly decreased (*P* < 0.01). Among the neurotransmitters of the choline metabolic pathway, choline in the fever model group compared with NG, and the content of acetylcholine increased (*P* < 0.01). Compared with MG, the contents of choline and acetylcholine increased in the 8-*O*aS-H, but there was no statistical significance.

**TABLE 3:** Hypothalamic neurotransmitter metabolite standard curves and levels in yeast-induced pyrexic rats.

Number	Name	Standard curve	*R* ^2^	A	B	C	Fold (B/A)	Fold (C/B)
1	5-HIAA	y = 2,626.64x + 492.15	0.9999	0.047215 ± 0.002552	0.006678 ± 0.000303**	0.034984 ± 0.001303^##^	0.141437204	5.238738
2	Histamine	y = 203.17x + 41.62	0.9999	0.198284 ± 0.008485	0.284496 ± 0.020837**	0.281854 ± 0.010514	1.434792752	0.990714
3	DL-Adrenaline	y = 1,650.72x + 603.62	0.9998	0.228375 ± 0.014434	0.219118 ± 0.02423	0.183271 ± 0.011758^##^	0.959465488	0.836407
4	Norepinephrine	y = 1,955.07x + 348.01	0.9999	3.083602 ± 0.13771	3.63352 ± 0.135757**	2.659784 ± 0.157092^##^	1.178336397	0.732013
5	5-hydroxytryptamine (Seronin/5-HT)	y = 264.94x + 36.57	0.9999	0.056736 ± 0.010095	0.163188 ± 0.013704**	0.070399 ± 0.005118^##^	2.876299653	0.4314
6	Dopamine	y = 639.94x + 143.84	0.9998	0.042024 ± 0.004151	0.036415 ± 0.001931**	0.041184 ± 0.003516^##^	0.866541623	1.130946
7	Histidine	y = 1,593.13x + 282.44	1.0000	1.471756 ± 0.074157	1.7916 ± 0.104177**	1.615396 ± 0.090799^##^	1.217321484	0.90165
8	5-Hydroxytryptophan (HTP)	y = 5,758.69x + 1,401.50	0.9994	0.06087 ± 0.016125	0.066134 ± 0.009746	0.058849 ± 0.01016	1.086489105	0.889842
9	Kynurenine (Kyn)	y = 40,895.20x + 3,465.60	0.9999	0.00278 ± 0.000711	0.006647 ± 0.000446**	0.005571 ± 0.000455^##^	2.391560225	0.838131
10	Tyramine	y = 1,338.47x + 20.75	0.9997	0.068404 ± 0.00367	0.050769 ± 0.00433**	0.03725 ± 0.002705^##^	0.742193192	0.733711
11	Tryptamine	y = 13,833.30x + 4,282.54	0.9999	0.071598 ± 0.08941	0.012142 ± 0.001635	0.007275 ± 0.001485^##^	0.169588777	0.59914
12	DL-METANEPHRINE	y = 273.52x + 77.00	0.9996	5.878193 ± 1.13383	6.340637 ± 0.53496	6.696331 ± 0.235348	1.07867102	1.056098
13	Homovanillic acid	y = 1,410.99x + 548.64	0.9999	0.058349 ± 0.003431	0.012015 ± 0.000969**	0.057274 ± 0.003378^##^	0.205917837	4.766814
14	L-Glutamic acid	y = 34,535.71x + 2,293.67	0.9999	47.71541 ± 5.049066	49.52209 ± 4.066673	27.4802 ± 1.644749^##^	1.037863664	0.554908
15	γ-Aminobutyric acid (GABA)	y = 80,208.06x + 4,534.85	0.9999	74.38002 ± 6.256568	88.90237 ± 4.827286**	85.24453 ± 4.772599	1.19524522	0.958856
16	D-Glutamine	y = 11,718.16x + 1,195.11	0.9999	13.36782 ± 2.059013	14.42443 ± 2.174848	13.70714 ± 0.95925	1.079041429	0.950273
17	Boc-D-Tyr-OH(Tyrosine)	y = 10,667.74x + 3,563.37	0.9999	3.890283 ± 0.410652	5.163715 ± 0.380754**	3.519054 ± 0.356443^##^	1.327336496	0.681497
18	Arginine	y = 5,661.67x + 1,446.60	0.9998	19.55715 ± 1.241994	22.62697 ± 0.768431**	13.51525 ± 0.472335^##^	1.156966607	0.597307
19	L-tryptophan	y = 63,209.18x + 1,904.67	0.9995	1.542211 ± 0.067428	2.310407 ± 0.240642**	1.912186 ± 0.116505^##^	1.498113506	0.82764
20	Phenprobamate (Phenylalanine)	y = 80,916.28x + 3,956.43	0.9996	9.408213 ± 11.68514	5.82411 ± 0.958279	4.009632 ± 0.247151^##^	0.619045245	0.688454
21	(2-acetoxyethyl)trimethylammonium	y = 919.63x + 161.03	0.9999	89.31803 ± 3.864719	118.3229 ± 8.798268**	119.2085 ± 4.188516	1.324737206	1.007484
22	Choline hydroxide	y = 22,660.63x + 5,005.46	0.9999	10.11443 ± 0.81096	10.6686 ± 1.010172	11.17756 ± 0.51638	1.054789388	1.047707

Note: **: *P* < 0.01. A: normal control group; B: fever model group; C: 8-*O*aS high dose group.

The pearman correlation analysis of intestinal flora and metabolomics between 8-*O*aS-H and the MG at the genus level (Figure 5d). It was worth noting that *Alistipes* have significant negative associated with 5-HIAA and homovanillic acid, and a significant positively associated with DL-adrenaline, NE, 5-HT, histidine, KYN, tyramine, tryptamine, glutamic acid, tyrosine, arginine, L-tryptophan and phenylalanine.

The pearman correlation analysis of intestinal flora and metabolomics between 8-*O*aS-H and the MG at the species level (Figure 5f). It is worth noting that *Alistipes*_*finegoldii* showed a significant negative correlation with 5-HIAA, DL-METANEPHRINE and homovanillic acid and a significant positive correlation with NE, 5-HT, arginine, L-tryptophan and phenylalanine.

### Utilizing metabolomic biomarkers in colon contents to elucidate the antipyretic and anti-inflammatory effects of 8-*O*aS

**FIGURE 6:**
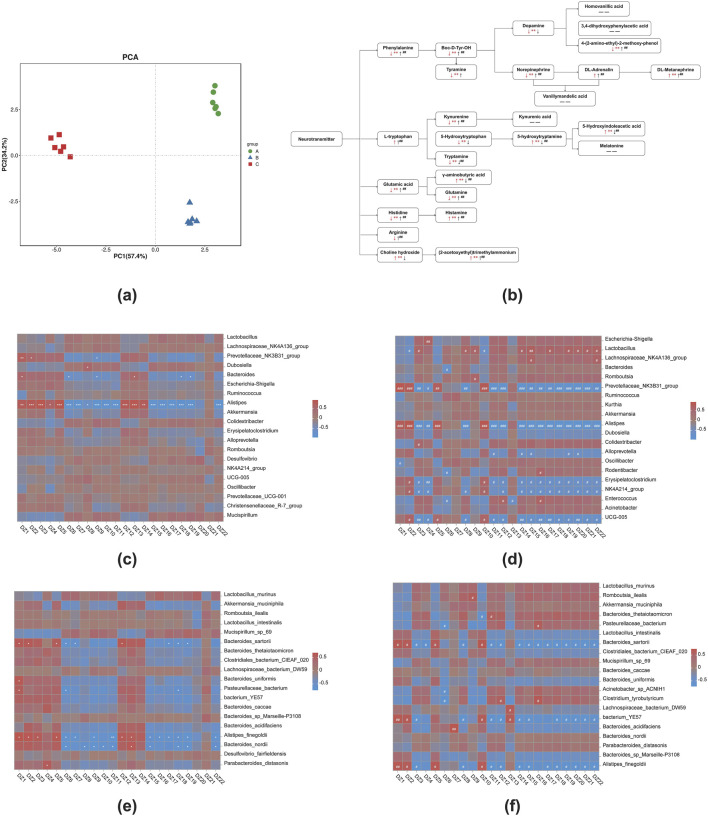
Metabolic analysis of potential biomarkers in colon contents. **(a)** PCA analysis; **(b)** the significant difference of 8-*O*aS on neurotransmitter metabolism in colon contents of yeast-induced pyrexia in rats (Ma et al., 2021) (↑: represents increase; ↓: represents decrease; arrows are red when the fever model group is compared with the control group; arrows are black when 8-*O*aS-H is compared with the fever model group. — —: indicates that the device is not detected.); **(c)** the heatmap of correlation analysis at genus level between NG and MG; **(d)** the heatmap of correlation analysis at genus level between MG and 8-*O*aS-H; **(e)** the heatmap of correlation analysis at species level between NG and MG; **(f)** the heatmap of correlation analysis at species level between MG and 8-*O*aS-H. (DZ1: 5-Hydroxyindoleacetic acid (5-HIAA); DZ2: γ-Aminobutyric acid (GABA); DZ3: Histamine; DZ4: DL-Adrenaline; DZ5: 5-hydroxytryptamine (Seronin; 5-HT); DZ6: Dopamine; DZ7: 5-Hydroxytryptophan (HTP); DZ8: Kynurenine (KYN); DZ9: Tyramine; DZ10: Tryptamine; DZ11: 4-(2-amino-ethyl)-2-methoxy-phenol (3-methoxytyramine); DZ12 (2-acetoxyethyl)trimethylammonium (acetylcholine; Ach); DZ13: Choline hydroxide; DZ14: DL-METANEPHRINE; DZ15: Glutamic acid; DZ16: Norepinephrine; DZ17: Glutamine; DZ18: Histidine; DZ19: Boc-D-Tyr-OH(Tyrosine); DZ20: Arginine; DZ21: L-tryptophan; DZ22: Phenylalanine.). Note: Compared with control group, *P < 0.05, **P < 0.01, ***P < 0.01; Compared with the fever model group, ^#^ *P* < 0.05, ^##^ *P* < 0.01, ^###^ *P* < 0.01. A: normal control group (NG); B: fever model group (MG); C: 8-*O*aS high dose group (8-*O*aS-H).

In colon contents (Figure 6; Table 4), the neurotransmitters associated with the phenylalanine metabolic pathway are affected. Compared with NG, the contents of phenylalanine, tyrosine, tyramine, dopamine, NE and 3-methoxytyramine(3-MT) in MG were significantly decreased (*P* < 0.01), while the content of DL-metanephrine was significantly increased (*P* < 0.01). The content of DL-adrenaline was increased. Compared with MG, the contents of phenylalanine, tyrosine, NE, DL-adrenaline, DL-metanephrine and 3-MT in 8-*O*aS-H were significantly increased (*P* < 0.01), while the content of tyramine was increased and dopamine content decreased, but the differences were not statistically significant. In the neurotransmitters of L-tryptophan metabolic pathway, compared with NG, the contents of 5-HT and 5-HIAA acid were significantly increased in MG (*P* < 0.01), while the content of L-tryptophan was increased but the difference was not statistically significant. The contents of KYN, 5-HTP and tryptamine were significantly decreased (*P* < 0.01). Compared with MG, the levels of 5-HT and 5-HIAA acid in 8-*O*aS-H were significantly decreased (*P* < 0.01), while the contents of L-tryptophan and KYN were significantly increased (*P* < 0.01). The content of 5-HTP and tryptamine (*P* < 0.01) was decreased, but the difference of 5-HTP was not statistically significant. In the neurotransmitter of glutamate metabolic pathway, compared with NG, the content of GABA acid in MG was significantly increased (*P* < 0.01), and the contents of glutamate and glutamine were significantly decreased (*P* < 0.01). Compared with the MG, the content of GABA in 8-*O*aS-H was significantly decreased (*P* < 0.01), and the contents of glutamate and glutamine were significantly increased (*P* < 0.01). In the neurotransmitters of the histidine metabolic pathway, the content of histidine in MG was significantly decreased (*P* < 0.01) and the content of histamine was significantly increased (P < 0.01) compared with the NG. Compared with MG, the histamine and histidine contents of 8-*O*aS-H were significantly increased (*P* < 0.01). In the neurotransmitter of arginine metabolic pathway, the content of arginine in MG decreased compared with NG. Compared with MG, the content of arginine in 8-*O*aS-H was significantly increased (P < 0.01). In the neurotransmitters of choline metabolic pathway, the contents of choline and Ach in MG were significantly increased compared with NG (P < 0.01). Compared with MG, the choline content in 8-*O*aS-H was decreased without statistical significance, but the Ach content was significantly increased (*P* < 0.01).

**TABLE 4:** Standard curves and levels of neurotransmitter metabolites in colon contents of yeast-induced pyrexic rats.

Number	Name	Standard curve	*R* ^2^	A	B	C	Fold (B/A)	Fold (C/B)
1	5-HIAA	y = 2,626.64x + 492.15	0.9999	0.034611 ± 0.001141	0.091692 ± 0.040368**	0.022985 ± 0.001039^##^	2.649208219	0.250672121
2	γ-Aminobutyric acid	y = 80,208.06x + 4,534.85	0.9999	0.123688 ± 0.004746	0.152367 ± 0.008428**	0.040604 ± 0.002325^##^	1.231869918	0.266488344
3	Histamine	y = 203.17x + 41.62	0.9999	0.060681 ± 0.005625	0.097872 ± 0.005082**	0.164993 ± 0.014521^##^	1.612896828	1.685800852
4	DL-Adrenaline	y = 1,650.72x + 603.62	0.9999	0.127347 ± 0.006225	0.138873 ± 0.011429	0.179385 ± 0.016034^##^	1.090508808	1.291717932
5	5-hydroxytryptamine (Seronin)	y = 264.94x + 36.57	0.9999	1.168141 ± 0.076248	2.781558 ± 0.142555**	2.315621 ± 0.128935^##^	2.381183441	0.832490537
6	Dopamine	y = 639.94x + 143.84	0.9999	0.038943 ± 0.003231	0.020562 ± 0.002681**	0.020301 ± 0.00356	0.528005139	0.987280518
7	5-Hydroxytryptophan (HTP)	y = 5,758.69x + 1,401.50	0.9994	0.321604 ± 0.042553	0.146566 ± 0.010702**	0.146348 ± 0.003778	0.455735646	0.998507497
8	Kynurenine (Kyn)	y = 40,895.20x + 3,465.60	0.9999	0.001188 ± 7.16E-05	0.001035 ± 5.92E-05**	0.002393 ± 0.000251^##^	0.871279755	2.31206051
9	Tyramine	y = 1,338.47x + 20.75	0.9997	0.036082 ± 0.001558	0.021609 ± 0.001885**	0.022758 ± 0.003372	0.598884422	1.053193395
10	Tryptamine	y = 13,833.30x + 4,282.54	0.9999	1.77937 ± 0.044348	0.824226 ± 0.076935**	0.204631 ± 0.01947^##^	0.463212168	0.248270931
11	4-(2-amino-ethyl)-2-methoxy-phenol	y = 11,364.20x + 2,282.29	0.9999	0.052284 ± 0.002038	0.038354 ± 0.002474**	0.06461 ± 0.00196^##^	0.733561299	1.68458336
12	(2-acetoxyethyl)trimethylammonium	y = 919.63x + 161.03	0.9999	3.952651 ± 0.146873	5.999216 ± 0.290192**	19.96216 ± 1.5319^##^	1.517770279	3.327460922
13	Choline hydroxide	y = 22,660.63x + 5,005.46	0.9999	0.208381 ± 0.013291	0.537794 ± 0.036806**	0.505666 ± 0.032299	2.580821527	0.940259739
14	DL-METANEPHRINE	y = 273.52x + 77.00	0.9996	0.12735 ± 0.023086	0.198776 ± 0.0109**	0.904836 ± 0.118869^##^	1.560867594	4.552045298
15	L-Glutamic acid	y = 34,535.71x + 2,293.67	0.9999	24.64342 ± 0.665618	18.73314 ± 0.389293**	34.41749 ± 4.302741^##^	0.760168078	1.83725093
16	Norepinephrine	y = 1,955.07x + 348.01	0.9999	13.20296 ± 1.505581	7.40493 ± 0.250628**	10.53341 ± 0.752077^##^	0.56085373	1.422486104
17	D-Glutamine	y = 11,718.16x + 1,195.11	0.9999	1.34784 ± 0.083598	0.68738 ± 0.023181**	0.827461 ± 0.031545^##^	0.509986024	1.203790782
18	Histidine	y = 1,593.13x + 282.44	1.0000	4.367248 ± 0.177801	2.537304 ± 0.218178**	10.32286 ± 0.610623^##^	0.580984631	4.068437864
19	Boc-D-Tyr-OH(Tyrosine)	y = 10,667.74x + 3,563.37	0.9999	9.704506 ± 0.808194	6.806136 ± 0.365195**	16.61662 ± 1.490747^##^	0.701337746	2.4414181
20	Arginine	y = 5,661.67x + 1,446.60	0.9998	10.82479 ± 9.89566	2.740709 ± 0.139097	12.7836 ± 0.994513^##^	0.253188265	4.664341902
21	L-tryptophan	y = 63,209.18x + 1,904.67	0.9995	0.508506 ± 0.051069	0.516647 ± 0.044808	4.698053 ± 0.404531^##^	1.016009302	9.093356516
22	P henprobamate (Phenylalanine)	y = 80,916.28x + 3,956.43	0.9996	9.14644 ± 0.25487	5.416307 ± 0.19539274**	15.6515 ± 3.007695^##^	0.592176468	2.889699713

Note: **: *P* < 0.01. A: normal control group; B: fever model group; C: 8-*O*aS high dose group.

The pearman correlation analysis of intestinal flora and metabolomics were performed between 8-*O*aS-H and the MG at the genus level (Figure 6d). *Alistipes* showed a significant negative correlation with histamine, DL-adrenaline, KYN, 3-methoxytyramine, Ach, DL-metanephrine, glutamic acid, NE, glutamine, histidine, tyrosine, arginine, L-tryptophan and phenylalanine and a significant positive correlation with 5-HIAA, GABA, 5-HT and tryptamine. At the species level (Figure 6f), It is worth noting that *Alistipes*_*finegoldii* showed a significant negative correlation with histamine, KYN, 3-methoxytyramine, Ach, DL-metanephrine, glutamic acid, glutamine, histidine, tyrosine, arginine, L-tryptophan and phenylalanine and a significant positive correlation with 5-HIAA, GABA, 5-HT and tryptamine between 8-*O*aS-H and the MG.

## Discussion

*Phlomoides rotata* contains a variety of chemical constituents such as iridoid glycosides, flavonoids and phenylethanol glycosides (Cui et al., 2020). Studies have shown that the iridoid glycosides extracts of *P. rotata* have a wide range of biological activities (Lei et al., 2023; Zheng et al., 2015). Among them, 8-*O*aS has multiple pharmacological effects, including analgesia, neuroprotection, and anti-anxiety (Li Y. J. et al., 2023; Sun et al., 2020; Zhang et al., 2018; Zhang et al., 2014). Our research group established a yeast-induced pyrexia model in rats, monitored the anal temperature of rats at various time points, selects three representative cyclohexene ether iridoid glycosides, namely, shanzhiside methylester, 8-*O*aS, and 8-deoxyshanzhiside, from *P. rotata* to investigate their antipyretic effects. In comparison to the fever model group, all treatment groups demonstrated antipyretic effects at 5 hours post-administration. Regarding the extent of body temperature reduction, the efficacy was ranked as follows: 8-*O*aS>8-deoxyshanzhiside > shanzhiside methylester, suggesting that 8-*O*aS displayed the most pronounced antipyretic effect and was comparable to that of the positive control group.

In this study, yeast-induced pyrexia model in rats was established by subcutaneous injection of 20% dry yeast on the back. After 5 h of modeling, compared with the NG, the body temperature of the other five groups showed a significant upward. Serum cytokines TNF-α, IL-6, IL-1β, MIP-1α, and IFN-γ were increased in the MG. Thermoregulatory mediator AVP and neurotransmitter 5-HT were reduced, PGE_2_, CRH, SP and serum NF-κB were higher in the MG. Compared with the MG, 8-*O*aS reversed the alterations in serum levels of cytokines in rats with yeast-induced pyrexia. Therefore, 8-*O*aS has a significant inhibitory effect on yeast-induced pyrexia in rats. It is suggested that the antipyretic mechanism may be related to down-regulating the expression of inflammatory factors and positive body temperature regulator, up-regulating the expression of negative body temperature regulator, and altering the content of neurotransmitters (5-HT) in serum.

The TLR4/NF-κB pathway is a bridge between exogenous and endogenous pyrogens in the fever progress. External stimuli cause TLR4 to undergo a series of phosphorylation cascade reactions, inducing phosphorylation and degradation of the NF-κB inhibitory protein IκB, which in turn leads to NF-κB activation and nuclear translocation. This triggers the expression and release of a variety of inflammatory factors (IL-1, IL-6, TNF-α, NO, and IFN-γ), leading to increased synthesis of COX-2 in the hypothalamus and elevated release of PGE_2_. Meanwhile, phospholipase C (PLC) was hydrolyzed to arachidonic acid (AA), which was catalyzed by COX-2 to produce PGE_2_. Endogenous pyrogens promote the release of positive thermoregulatory mediators, causing the thermoregulatory point to rise. It also acts on the negative thermoregulatory center to release negative regulatory mediators and inhibit hyperthermia (Evans et al., 2015; Cannon, 2013; Wrotek et al., 2020). The TLR4/NF-κB signaling pathway activated by various factors is critical for the fever course. In recent years, the antipyretic mechanisms of certain Chinese medicines, herbal extracts, and formulations (such as Tetrastigma hemsleyanum polysaccharide and baicalin) have been reported to be closely associated with this pathway. (Fu et al., 2024; Ye et al., 2015). As a result of this experiment, we found that TLR4 and NF-κB levels were elevated in rats with yeast-induced pyrexia. 8-*O*aS significantly inhibited the expression of TLR4 and NF-κB while elevating the p-IκB. It is suggested that 8-*O*aS can exert antipyretic effects through TLR4/NF-κB pathway. HSP70 family is a protective important molecule under heat stress state, rarely expressed in normal cells, but express in the heat stress (Ghadhanfar et al., 2019). Meanwhile, it has been shown that HSP70 limits body temperature increases by inhibiting NF-κB activation and endogenous thermogenesis (Lee et al., 2012). The results indicated that the expression levels of HSP70 mRNA and protein were elevated in rats with yeast-induced pyrexia, which was inhibited by 8-*O*aS. Transient receptor potential vanilloid 4 (TRPV4), a member of the transient receptor potential ion channel family, is widely expressed in the body, particularly in the preoptic area of the hypothalamus, and plays a role in regulating body temperature. By replicating the zebra fish fever model, it was found that TRPV1, TRPV4, and PGE_2_ co-regulated the development of fever, and the decreased expression of TRPV4 contributed to the generation of fever (Lee et al., 2005). However, Wang and colleagues (Wang et al., 2008) found that TRPV4 was increased in the hypothalamus of LPS-induced pyrexia in rats, which led to an increase in calcium influx and then upregulation of cAMP expression to mediate the fever process. TRPV4 was activated to participate in thermoregulation, but the specific mechanism of activation has not been clarified. The results showed that TRPV4 mRNA expression was increased in the MG. Nonetheless, the treatment of 8-*O*aS resulted in a downregulation of TRPV4 mRNA expression. The COX2/PGE_2_/EP3/cAMP pathway in the hypothalamus is an important positive regulatory pathway during fever (Zhang Y. Q. et al., 2022). The results of this study revealed that the expression of COX2 in the hypothalamus was elevated during fever, and decreased after 8-*O*aS administration. To summarise, 8-*O*aS reduces thermogenic cytokines and downregulates central thermoregulatory mediator PGE_2_. In addition, the mechanism of antipyretic effect of 8-*O*aS involves several pathways such as the TLR4/NF-κB pathway and the HSP70/NF-κB pathway.

The results of this experimental study showed that, at the genus level, the relative abundance of *Alistipes* and *Odoribacter* was significantly higher after model establishment and significantly lower following drug administration. At the species level, the relative abundance of *Alistipes_finegoldii* was significantly increased after modeling and significantly decreased after drug administration. *Alistipes*, *Alistipes_finegoldii* and *Odoribacter* have pro-inflammatory properties in rats with yeast-induced pyrexia. To summarise, 8-*O*aS decreases the abundance of *Alistipes*, *Alistipes_finegoldii* and *Odoribacter* thereby exerting antipyretic and anti-inflammatory effects.

*Alistipes*, *Alistipes_finegoldii* and *Odoribacter* are reported to be closely related to various diseases. *Alistipes* has been reported to have a close association with various conditions, including Alzheimer’s disease (AD), diabetes, colitis, depression and other diseases, and has a beneficial and harmful dual role (Jiang et al., 2015; Song et al., 2024; Ma et al., 2023; Zhang Q. et al., 2022; Wang et al., 2021; Wu et al., 2021; Vogt et al., 2017). *Alistipes* with pro-inflammatory properties are significantly increased in the obese population (Andoh et al., 2016; Xu et al., 2022). Under high-temperature conditions, *Alistipes* level in the intestinal flora of broilers was increased, whereas it was reduced in the antibiotic group (Li S. et al., 2023). *Alistipes finegoldi* is a gram-negative anaerobic bacterium, belongs to the genus *Alistipes* (Parker et al., 2020). The study found that *A. finegoldi* are closely related to colon cancer and inflammatory bowel disease (Radka et al., 2020; He et al., 2022; Yin et al., 2022; Zhao et al., 2020; Kang et al., 2023; Dai et al., 2018). *Odoribacter* is strictly anaerobic and is negatively correlated with the severity of NAFLD and inflammatory bowel disease, due to its anti-inflammatory activity by producing short-chain fatty acids (SCFAs) such as butyric acid (Xie et al., 2023). *Odoribacter splanchnicus* has also been associated with a positive response to fecal transplantation therapy in patients with ulcerative colitis, which correlates with reduced intestinal inflammation (Saleh et al., 2023; Zhang et al., 2020). However, it has also been suggested that *Odoribacter* has pro-inflammatory properties and is pathogenic: *Odoribacter* was inhibited in colitis mice with the human strain transplantation treatment (Dai et al., 2024). The probiotic mixture VSL#3 reduced the relative abundance of *Oscillibacter* in the intestinal flora of colitis-associated carcinogenesis mice model (Wang et al., 2018).

As a crucial source of peripheral neurotransmitters and hormones, intestinal flora exerts both direct and indirect effects on hypothalamic neurons via the bidirectional gut-brain axis connection. Given the pivotal role of the hypothalamus in thermoregulation, it is posited that intestinal flora may regulate heat production and body temperature via neurotransmitters within the gut-brain axis.

In the phenylalanine pathway, this study showed a significant decline in phenylalanine content in hypothalamic tissue after modeling and a significant decrease after 8-*O*aS administration. In contrast, the phenylalanine content was significantly reduced after modeling and significantly increased after 8-*O*aS administration in the colon contents. Phenylalanine is converted to tyrosine in the production of neurotransmitters, which are then converted to dopamine, NE, and DL-adrenaline. Some studies have found that serum phenylalanine decreased in the LPS induced fever model, and *Huanglian-Jiedu* decoction can reverse this phenomenon (Li et al., 2021).

In the tryptophan pathway, this study showed increased levels of tryptophan, KYN, 5-HTP and 5-HT and decreased levels of 5-HIAA and tryptamine in hypothalamic tissue after modeling. However, after 8-*O*aS administration, hypothalamic levels of tryptophan, KYN, 5-HTP, 5-HT and tryptophan were significantly decreased, and the levels of 5-HIAA were significantly higher. In contrast, the levels of tryptophan, 5-HT and 5-HIAA in colonic contents were increased and the levels of 5-HTP, KYN and tryptamine were decreased after modeling. But the levels of 5-HTP, 5-HT, 5-HIAA and tryptamine were decreased, and the levels of tryptophan, KYN were significantly increased after 8-*O*aS administration.

*Alistipes* could convert tryptophan to indole, have tryptophan metabolizing activity (Parker et al., 2020). Correlation analysis results showed that the relative abundance of *Alistipes* in intestinal flora significantly increased after modeling, and significantly decreased after 8-*O*aS administration. There was a significant positive correlation between *Alistipes* and body temperature, tryptophan, and 5-HT. Changes in *Alistipes* levels play an important role in regulating body temperature. Li et al. also found that compared with the room temperature control group, the relative abundance of *Alistipes* in the cecum of the high-temperature control group was increased, accompanied by increased expression of 5-HT2A and TPH2 (*P* < 0.05) (Li S. et al., 2023). Peripheral 5-HT cannot cross the BBB into the CNS, indicating that the central and peripheral 5-HT systems are completely independent. However, tryptophan, a precursor of 5-HT, can cross the BBB and then be metabolized to 5-HT through the catalytic conversion of central TPH2 or to KYN through the TDO pathway. In this study, although 5-HT cannot directly cross the BBB, tryptophan and its metabolic enzymes can increase the hypothalamic 5-HT content, which may link *Alistipes* with brain function. Furthermore, the function of central and peripheral 5-HT is different. Hypothalamic 5-HT is an important neurotransmitter involved in regulating physiological processes such as sleep, mood, and body temperature. It has been reported that the tryptophane-5-HT metabolic pathway in the hypothalamus is involved in the regulation of brain neurotransmitters, and intracerebroventricular administration of 5-HT can prevent hypotension and hypothermia (Mota et al., 2019). Systemic 5-HT-induced inhibition of BAT SNA requires GABAergic inhibition of BAT sympathetic excitatory neurons in the DMH. In addition, the endogenous release of 5-HT from rRPa (pallid nucleus of the medullary cephalic intermediate suture) inhibits BAT SNA during warming (Mota et al., 2020). In conclusion, the changes in *Alistipes* abundance and hypothalamic 5-HT content may be the potential causes of thermoregulation in rats with yeast-induced pyrexia.

The glutamic acid metabolic pathway also plays an important role in the fever process. As an important neurotransmitter for synaptic excitation in the central nervous system, it acts a crucial role in rapid synaptic transmission, learning and memory, as well as the development of the nervous system. Glutamatergic nerves distributed in the preoptic area (POA) of the hypothalamus and the glutamatergic ionotropic receptor NMDA play an important role in thermoregulation and fever (Machado et al., 2020; Krajewski-Hall et al., 2019; Hou et al., 2011; Simm et al., 2016; Morrison, 2016). Microinjection of L-glutamic acid (0.14 nM) into the POA of awake rats caused an increase in body temperature and brain temperature (Sengupta et al., 2014). Glutamic acid increases body heat production and decreases heat dissipation through NMDA, resulting in a fever effect. The application of NMDA-receptor antagonists can significantly reduce glutamate-induced fever (Hou et al., 2011). Glutamatergic preoptic neurons expressing EP3R mediate inflammatory fever (Machado et al., 2020). The use of paracetamol reduces glutamate concentrations (Blecharz-Klin et al., 2019). AS an excitatory neurotransmitter, it is utilized by end-vascular organs and causes an increase in body temperature leading to hyperthermia (Sterkel et al., 2017). Glutamate may be involved in orexin neurons associated with PGE2-induced fever (Takahashi et al., 2013). LPS injected intraperitoneally induces an increase in glutamate (Chang et al., 2013; Huang et al., 2008; Kao et al., 2011). Activation of glutamate receptors in the dorsomedial hypothalamus (DMH) is required for COX-independent, IL-1β-induced BAT thermogenesis (Mota and Madden, 2022). This study shows that hypothalamic glutamate levels were elevated after modeling and significantly decreased after 8-*O*aS administration. GABA, metabolized from glutamic acid, is the major inhibitory neurotransmitter produced and regulated mainly by astrocytes and neurons. GABA levels in the hypothalamus and colon content were significantly increased after modeling and decreased after 8-*O*aS administration. Therefore, the alterations in glutamic acid and GABA levels may serve as potential contributors to thermoregulation in rats with yeast-induced pyrexia by 8-*O*aS. The intestinal flora is involved in GABA production, as manipulation of the intestinal microbiota has been shown to affect GABA levels. Several intestinal microbes have been identified as GABA producers, including members of the genera *Anabacterium*, *Bifidobacterium*, and *Lactobacillus* (Eicher and Mohajeri, 2022). Xiaochaihu granules alleviate yeast-induced fever by modulating inflammatory/immune responses, neuromodulation, and metabolic modules (Chen et al., 2024). They released GABA predominantly from axons in DMH and their GABAergic terminals are increased due to prolonged heat exposure. These findings suggest that tonic gabaergic inhibitory signaling EP3R series neurons from the POA are fundamental determinants of body temperature for thermal homeostasis and fever (Nakamura et al., 2022).

The results of the histidine metabolism pathway showed that the contents of histidine and histamine in the hypothalamus were increased significantly after modeling, and the contents of histidine and histamine were decreased after 8-*O*aS administration. Histamine in the hypothalamic neurons regulate adaptive behavior and generate heat, in response to endogenous pyrogen (Sakata et al., 1995). The PO/AH region containing thermoregulatory neurons was identified as the main site where histamine affects body temperature. Most studies have found that histamine has a hyperthermic effect (Tabarean, 2016). However, the content of histidine was decreased significantly and histamine was increased significantly in the colon content after modeling. After 8-*O*aS administration, histidine and histamine content were significantly increased.

Dietary L-arginine supplementation maintains intestinal epithelial integrity during exercise under heat stress through a mechanism independent of T (core) regulation (Costa et al., 2014). Our study arginine pathway results showed a significant increase in hypothalamic tissue arginine content after modeling and a significant decrease after 8-*O*aS administration. Arginine content of colonic contents decreased and significantly increased after 8-*O*aS administration, it is suggested that arginine is involved in the regulation of fever process and 8-*O*aS treatment.

The choline pathway also plays an important role in the mechanism of fever. As an important parasympathetic neurotransmitter, Ach increased significantly in the hypothalamus after modeling, suggesting that Ach is involved in thermoregulation, which is consistent with the literature (Fu et al., 1996). However, there was no significant change in the hypothalamus after 8-*O*aS administration, suggesting that the drug had little effect on hypothalamic Ach. Levels of Ach in colon contents were significantly increased after modeling and administration. Choline, a precursor of the neurotransmitter Ach, showed no significant change in hypothalamic content but significantly increased in colon contents after modeling, but had no significant effect after administration. Ventricular injection of Ach 1 mg/only in rats increases body temperature (Simpson and Resch, 1986). 5-HT and Ach cause an increase in body temperature, and NE causes a decrease in body temperature, which is maintained by antagonistic effects on neurons in the preoptic area-anterior hypothalamus. Spleen meridian herbs regulate body temperature in rats with yeast-induced fever. Administration of Astragali Radix, a herb with warming properties, exacerbated the hyperthermia in yeast-induced pyrexic rats concomitant with elevated plasma choline concentrations. The cooling herbs Nelumbinis Semen and Coicis Semen decreased body temperature in yeast-induced pyrexic rats, accompanied by an increase in plasma histidine (Huang et al., 2020). Magnetic resonance (MRS) revealed an increase in the cerebellar acetylcholine/creatine ratio in febrile patients during fever compared to after recovery (*P* = 0.043) (Yang and Li, 2018). Behavioral fever leads to a significant increase in cholinergic neurotransmitter and receptor activity in target tissues (Sanhueza et al., 2021). The increase in brain Ach was accompanied by a decrease in 5-HT and 5-HIAA (Rodrigues et al., 2009).

## Conclusion

The 8-*O*aS yielded a good antipyretic and anti-inflammatory effect, and this efficacy was related to the improvement of the intestinal flora and neurotransmitters metabolites. There was a significant positive correlation between *Alistipes* and body temperature, tryptophan, and 5-HT. Changes in *Alistipes* levels play an important role in regulating body temperature. In conclusion, the changes in Alistipes abundance and hypothalamic 5-HT content may be the potential causes of thermoregulation in pyrexic rats by 8-*O*aS.

## Data Availability

The original contributions presented in the study are included in the article/Supplementary Material, further inquiries can be directed to the corresponding authors.
