# Supplementary material for: Elucidation of the antipyretic and anti-inflammatory effect of 8-O-Acetyl Shanzhiside methyl ester based on intestinal flora and metabolomics analysis
Source: Front Pharmacol. 2025 Apr 28;16:1482323. doi: 10.3389/fphar.2025.1482323 (PMC12066650; doi:10.3389/fphar.2025.1482323)
Supplement: Supplementary file 1 [file Table1.docx]

Table1 MRM acquisition parameters for mass spectrometry

| **Q1 Mass（Da）** | **Q3 Mass（Da）** | **ID** | **DP(V)** | **CE(V)** | **CXP(V)** |
| --- | --- | --- | --- | --- | --- |
| 148 | 102 | 1_Glutamic | 72 | 15 | 6 |
| 192 | 146 | 2-HIAA | 47 | 27 | 9 |
| 104 | 87 | 3_Aminobutyric_2 | 43 | 14 | 5 |
| 112 | 68 | 4_Histamindihy_1 | 55 | 29 | 8 |
| 112 | 95 | 4_Histamindihy_2 | 79 | 19 | 12 |
| 166 | 121 | 5_Norcephrine_1 | 28 | 17 | 10 |
| 166 | 134 | 5_Norcephrine_2 | 28 | 17 | 10 |
| 177 | 160 | 6_Seronin(5-HT) | 53 | 14 | 9 |
| 154 | 137 | 7_DA | 89 | 15 | 5 |
| 147 | 84 | 8_Glutamine_1 | 63 | 23 | 5 |
| 147 | 130 | 8_Glutamine_2 | 67 | 13 | 7 |
| 76 | 59 | 9_Histidine | 92 | 15 | 7 |
| 182 | 136 | 10_Tyrosine | 104 | 18 | 8 |
| 175 | 60 | 11_Arginine_1 | 163 | 17 | 4 |
| 175 | 70 | 11_Arginine_2 | 186 | 19 | 4 |
| 205 | 146 | 12_Tryptophan_1 | 76 | 24 | 8 |
| 205 | 188 | 12_Tryptophan_2 | 76 | 14 | 10 |
| 166 | 120 | 13_Phenylalanine | 26 | 13 | 4 |
| 221 | 204 | 14_HTP | 56 | 10 | 4 |
| 209 | 94 | 15_Kyn | 85 | 19 | 5 |
| 161 | 117 | 16_Tryptamine_1 | 64 | 27 | 7 |
| 161 | 144 | 16_Tryptamine_2 | 181 | 11 | 1 |
| 104 | 60 | 17_Choline_1 | 223 | 46 | 10 |
| 232.7 | 191 | 18_Melatonine | 92 | 13 | 11 |
| 180 | 148 | 19_Adrcnalin | 30 | 17 | 10 |
| 121 | 91 | 20_Tyramine | 100 | 15 | 11 |
| 168 | 91 | 21_methoxy | 89 | 25 | 10 |
| 188 | 143.6 | 22_Kynruenic_1 | -163 | -26 | -8 |
| 129 | 44.9 | 23_hydroxide | -55 | -20 | -5 |
| 196 | 136.8 | 24_Metanephrine | -60 | -27 | -8 |
| 197 | 136.9 | 25_Hydroxy | -112 | -44 | -5 |
| 181.1 | 151 | 26_Homovanillic | -60 | -9 | -9 |
| 167 | 123 | 27_DOPAC | -56 | -16 | -14 |
