# Supplementary material for: Elucidation of the antipyretic and anti-inflammatory effect of 8-O-Acetyl Shanzhiside methyl ester based on intestinal flora and metabolomics analysis
Source: Front Pharmacol. 2025 Apr 28;16:1482323. doi: 10.3389/fphar.2025.1482323 (PMC12066650; doi:10.3389/fphar.2025.1482323)
Supplement: Supplementary file 2 [file Table2.docx]

table 1 standard curve of 8-OaS on neurotransmitter metabolites in hypothalamic tissues of yeast-induced pyrexia rats

| number | name | standard curve | R^2^ | A | B | C | fold(B/A) | fold(C/B) |
| --- | --- | --- | --- | --- | --- | --- | --- | --- |
| 1 | 5-HIAA | y=2626.64x+492.15 | 0.9999 | 0.047215±0.002552 | 0.006678±0.000303^**^ | 0.034984±0.001303^##^ | 0.141437204 | 5.238738 |
| 2 | Histamine | y=203.17x+41.62 | 0.9999 | 0.198284±0.008485 | 0.284496±0.020837^**^ | 0.281854±0.010514 | 1.434792752 | 0.990714 |
| 3 | DL-Adrenalin | y=1650.72x+603.62 | 0.9998 | 0.228375±0.014434 | 0.219118±0.02423 | 0.183271±0.011758^##^ | 0.959465488 | 0.836407 |
| 4 | Norepinephrine | y=1955.07x+348.01 | 0.9999 | 3.083602±0.13771 | 3.63352±0.135757^**^ | 2.659784±0.157092^##^ | 1.178336397 | 0.732013 |
| 5 | 5-hydroxytryptamine(Seronin) | y=264.94x+36.57 | 0.9999 | 0.056736±0.010095 | 0.163188±0.013704^**^ | 0.070399±0.005118^##^ | 2.876299653 | 0.4314 |
| 6 | Dopamine | y=639.94x+143.84 | 0.9998 | 0.042024±0.004151 | 0.036415±0.001931^**^ | 0.041184±0.003516^##^ | 0.866541623 | 1.130946 |
| 7 | Histidine | y=1593.13x+282.44 | 1.0000 | 1.471756±0.074157 | 1.7916±0.104177^**^ | 1.615396±0.090799^##^ | 1.217321484 | 0.90165 |
| 8 | 5-Hydroxytryptophan(HTP) | y=5758.69x+1401.50 | 0.9994 | 0.06087±0.016125 | 0.066134±0.009746 | 0.058849±0.01016 | 1.086489105 | 0.889842 |
| 9 | Kynurenine(Kyn) | y=40895.20x+3465.60 | 0.9999 | 0.00278±0.000711 | 0.006647±0.000446^**^ | 0.005571±0.000455^##^ | 2.391560225 | 0.838131 |
| 10 | Tyramine | y=1338.47x+20.75 | 0.9997 | 0.068404±0.00367 | 0.050769±0.00433^**^ | 0.03725±0.002705^##^ | 0.742193192 | 0.733711 |
| 11 | Tryptamine | y=13833.30x+4282.54 | 0.9999 | 0.071598±0.08941 | 0.012142±0.001635 | 0.007275±0.001485^##^ | 0.169588777 | 0.59914 |
| 12 | DL-METANEPHRINE | y=273.52x+77.00 | 0.9996 | 5.878193±1.13383 | 6.340637±0.53496 | 6.696331±0.235348 | 1.07867102 | 1.056098 |
| 13 | Homovanillic acid | y=1410.99x+548.64 | 0.9999 | 0.058349±0.003431 | 0.012015±0.000969^**^ | 0.057274±0.003378^##^ | 0.205917837 | 4.766814 |
| 14 | L-Glutamic acid | y=34535.71x+2293.67 | 0.9999 | 47.71541±5.049066 | 49.52209±4.066673 | 27.4802±1.644749^##^ | 1.037863664 | 0.554908 |
| 15 | γ-Aminobutyric acid | y=80208.06x+4534.85 | 0.9999 | 74.38002±6.256568 | 88.90237±4.827286^**^ | 85.24453±4.772599 | 1.19524522 | 0.958856 |
| 16 | D-Glutamine | y=11718.16x+1195.11 | 0.9999 | 13.36782±2.059013 | 14.42443±2.174848 | 13.70714±0.95925 | 1.079041429 | 0.950273 |
| 17 | Boc-D-Tyr-OH(Tyrosine) | y=10667.74x+3563.37 | 0.9999 | 3.890283±0.410652 | 5.163715±0.380754^**^ | 3.519054±0.356443^##^ | 1.327336496 | 0.681497 |
| 18 | Arginine | y=5661.67x+1446.60 | 0.9998 | 19.55715±1.241994 | 22.62697±0.768431^**^ | 13.51525±0.472335^##^ | 1.156966607 | 0.597307 |
| 19 | L-tryptophan | y=63209.18x+1904.67 | 0.9995 | 1.542211±0.067428 | 2.310407±0.240642^**^ | 1.912186±0.116505^##^ | 1.498113506 | 0.82764 |
| 20 | Phenprobamate(Phenylalanine) | y=80916.28x+3956.43 | 0.9996 | 9.408213±11.68514 | 5.82411±0.958279 | 4.009632±0.247151^##^ | 0.619045245 | 0.688454 |
| 21 | (2-acetoxyethyl)trimethylammonium | y=919.63x+161.03 | 0.9999 | 89.31803±3.864719 | 118.3229±8.798268^**^ | 119.2085±4.188516 | 1.324737206 | 1.007484 |
| 22 | Choline hydroxide | y=22660.63x+5005.46 | 0.9999 | 10.11443±0.81096 | 10.6686±1.010172 | 11.17756±0.51638 | 1.054789388 | 1.047707 |

table 2 standard curve of 8-OaS on neurotransmitter metabolism in colon contents of yeast-induced pyrexia rats

| number | name | standard curve | R^2^ | A | B | C | fold(B/A) | fold(C/B) |
| --- | --- | --- | --- | --- | --- | --- | --- | --- |
| 1 | 5-HIAA | y=2626.64x+492.15 | 0.9999 | 0.034611±0.001141 | 0.091692±0.040368^**^ | 0.022985±0.001039^##^ | 2.649208219 | 0.250672121 |
| 2 | γ-Aminobutyric acid | y=80208.06x+4534.85 | 0.9999 | 0.123688±0.004746 | 0.152367±0.008428^**^ | 0.040604±0.002325^##^ | 1.231869918 | 0.266488344 |
| 3 | Histamine | y=203.17x+41.62 | 0.9999 | 0.060681±0.005625 | 0.097872±0.005082^**^ | 0.164993±0.014521^##^ | 1.612896828 | 1.685800852 |
| 4 | DL-Adrenalin | y=1650.72x+603.62 | 0.9999 | 0.127347±0.006225 | 0.138873±0.011429 | 0.179385±0.016034^##^ | 1.090508808 | 1.291717932 |
| 5 | 5-hydroxytryptamine(Seronin) | y=264.94x+36.57 | 0.9999 | 1.168141±0.076248 | 2.781558±0.142555^**^ | 2.315621±0.128935^##^ | 2.381183441 | 0.832490537 |
| 6 | Dopamine | y=639.94x+143.84 | 0.9999 | 0.038943±0.003231 | 0.020562±0.002681^**^ | 0.020301±0.00356 | 0.528005139 | 0.987280518 |
| 7 | 5-Hydroxytryptophan(HTP) | y=5758.69x+1401.50 | 0.9994 | 0.321604±0.042553 | 0.146566±0.010702^**^ | 0.146348±0.003778 | 0.455735646 | 0.998507497 |
| 8 | Kynurenine(Kyn) | y=40895.20x+3465.60 | 0.9999 | 0.001188±7.16E-05 | 0.001035±5.92E-05^**^ | 0.002393±0.000251^##^ | 0.871279755 | 2.31206051 |
| 9 | Tyramine | y=1338.47x+20.75 | 0.9997 | 0.036082±0.001558 | 0.021609±0.001885^**^ | 0.022758±0.003372 | 0.598884422 | 1.053193395 |
| 10 | Tryptamine | y=13833.30x+4282.54 | 0.9999 | 1.77937±0.044348 | 0.824226±0.076935^**^ | 0.204631±0.01947^##^ | 0.463212168 | 0.248270931 |
| 11 | 4-(2-amino-ethyl)-2-methoxy-phenol | y=11364.20x+2282.29 | 0.9999 | 0.052284±0.002038 | 0.038354±0.002474^**^ | 0.06461±0.00196^##^ | 0.733561299 | 1.68458336 |
| 12 | (2-acetoxyethyl)trimethylammonium | y=919.63x+161.03 | 0.9999 | 3.952651±0.146873 | 5.999216±0.290192^**^ | 19.96216±1.5319^##^ | 1.517770279 | 3.327460922 |
| 13 | Choline hydroxide | y=22660.63x+5005.46 | 0.9999 | 0.208381±0.013291 | 0.537794±0.036806^**^ | 0.505666±0.032299 | 2.580821527 | 0.940259739 |
| 14 | DL-METANEPHRINE | y=273.52x+77.00 | 0.9996 | 0.12735±0.023086 | 0.198776±0.0109^**^ | 0.904836±0.118869^##^ | 1.560867594 | 4.552045298 |
| 15 | L-Glutamic acid | y=34535.71x+2293.67 | 0.9999 | 24.64342±0.665618 | 18.73314±0.389293^**^ | 34.41749±4.302741^##^ | 0.760168078 | 1.83725093 |
| 16 | Norepinephrine | y=1955.07x+348.01 | 0.9999 | 13.20296±1.505581 | 7.40493±0.250628^**^ | 10.53341±0.752077^##^ | 0.56085373 | 1.422486104 |
| 17 | D-Glutamine | y=11718.16x+1195.11 | 0.9999 | 1.34784±0.083598 | 0.68738±0.023181^**^ | 0.827461±0.031545^##^ | 0.509986024 | 1.203790782 |
| 18 | Histidine | y=1593.13x+282.44 | 1.0000 | 4.367248±0.177801 | 2.537304±0.218178^**^ | 10.32286±0.610623^##^ | 0.580984631 | 4.068437864 |
| 19 | Boc-D-Tyr-OH(Tyrosine) | y=10667.74x+3563.37 | 0.9999 | 9.704506±0.808194 | 6.806136±0.365195^**^ | 16.61662±1.490747^##^ | 0.701337746 | 2.4414181 |
| 20 | Arginine | y=5661.67x+1446.60 | 0.9998 | 10.82479±9.89566 | 2.740709±0.139097 | 12.7836±0.994513^##^ | 0.253188265 | 4.664341902 |
| 21 | L-tryptophan | y=63209.18x+1904.67 | 0.9995 | 0.508506±0.051069 | 0.516647±0.044808 | 4.698053±0.404531^##^ | 1.016009302 | 9.093356516 |
| 22 | Phenprobamate(Phenylalanine) | y=80916.28x+3956.43 | 0.9996 | 9.14644±0.25487 | 5.416307±0.19539274^**^ | 15.6515±3.007695^##^ | 0.592176468 | 2.889699713 |
