# Supplementary material for: Elucidation of the antipyretic and anti-inflammatory effect of 8-O-Acetyl Shanzhiside methyl ester based on intestinal flora and metabolomics analysis
Source: Front Pharmacol. 2025 Apr 28;16:1482323. doi: 10.3389/fphar.2025.1482323 (PMC12066650; doi:10.3389/fphar.2025.1482323)

#### **Sample Name:** A_1 **Vial #:** 40

####
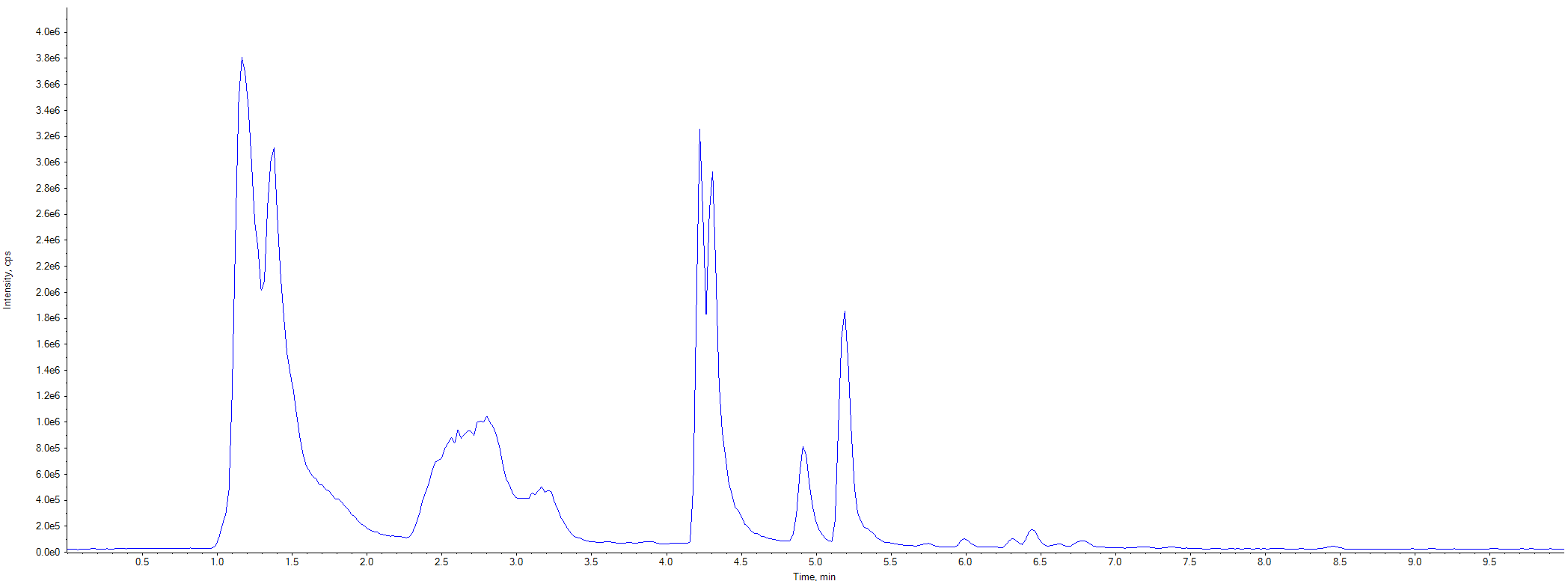


#### **Sample Name:** A_2 **Vial #:** 41

####
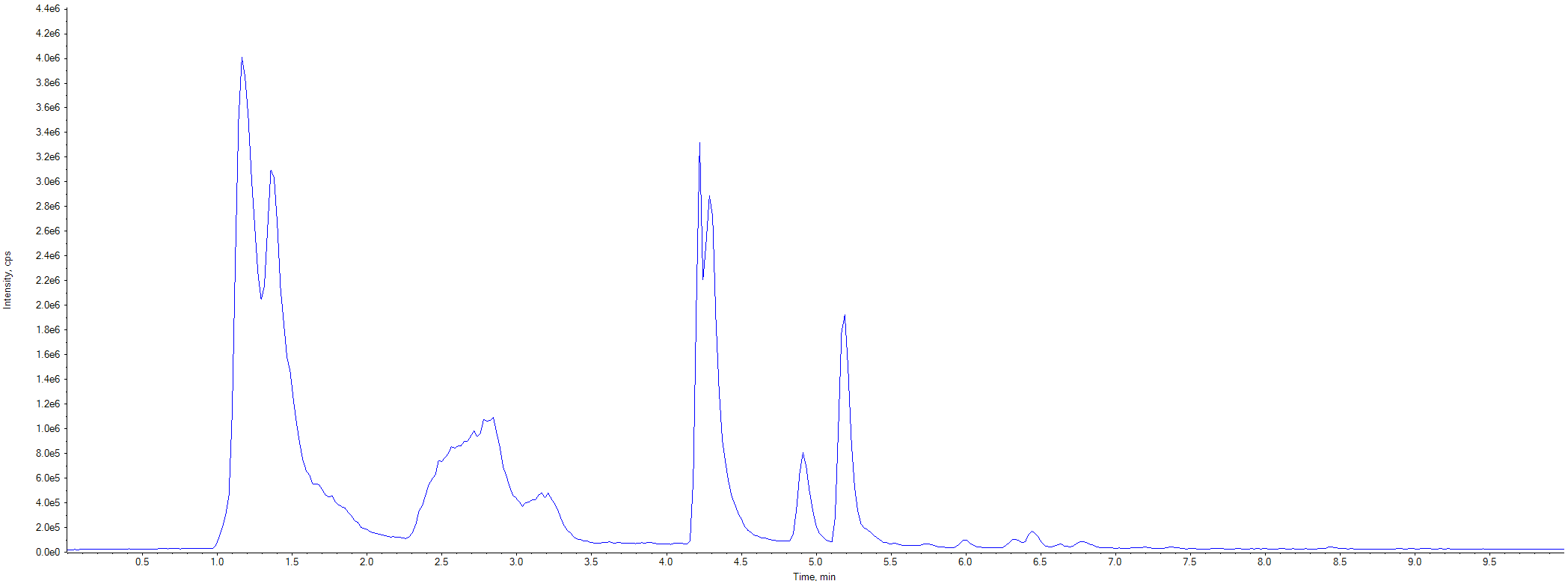


#### **Sample Name:** A_3 **Vial #:** 42

####
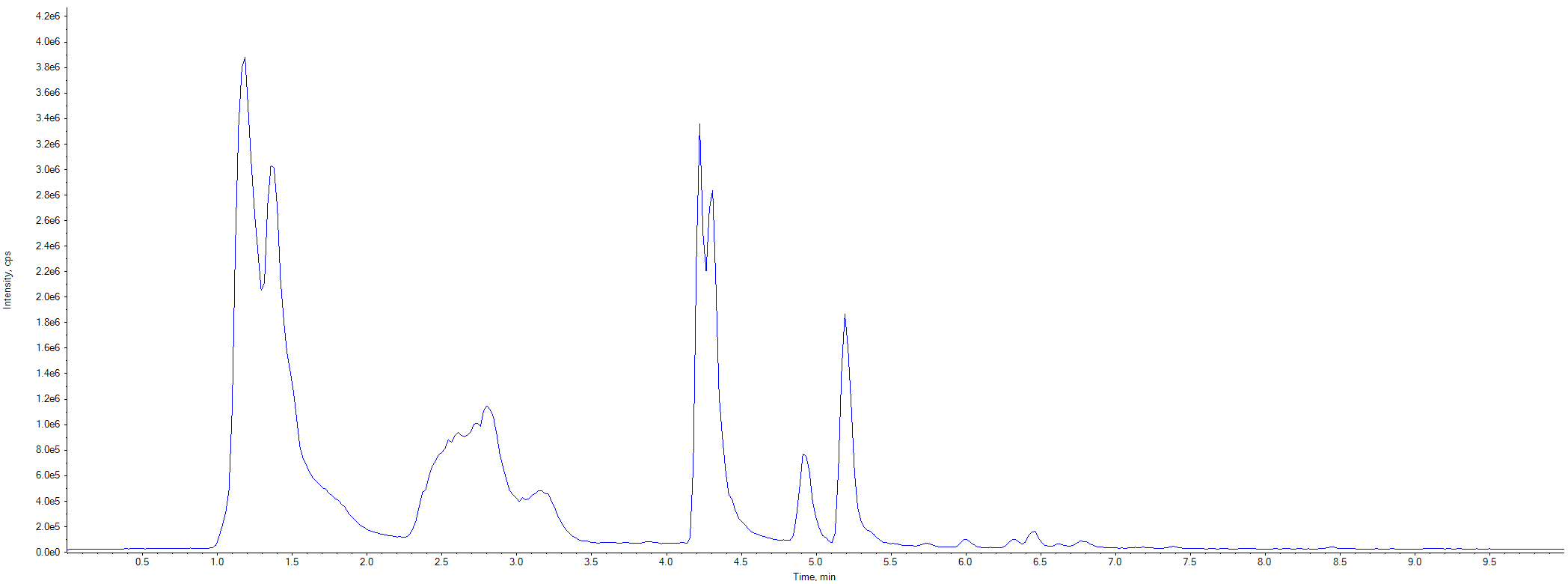


#### **Sample Name:** A_4 **Vial #:** 43

####
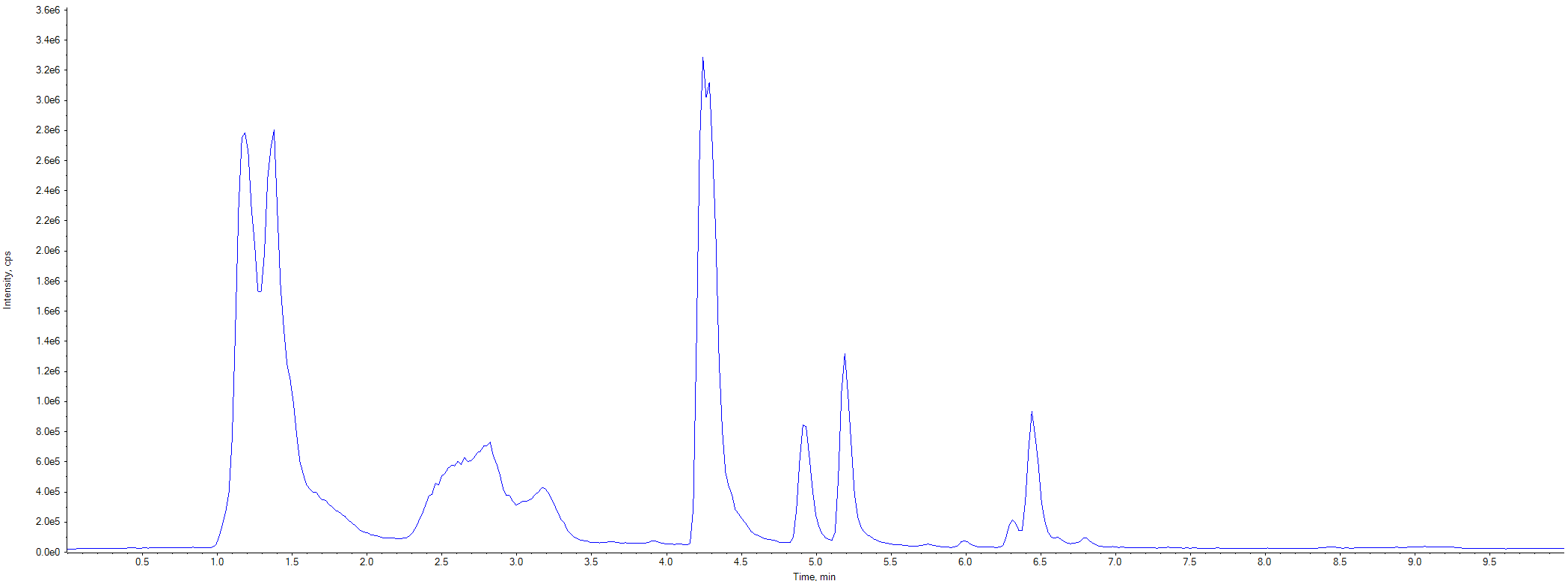


#### **Sample Name:** A_5 **Vial #:** 44

####
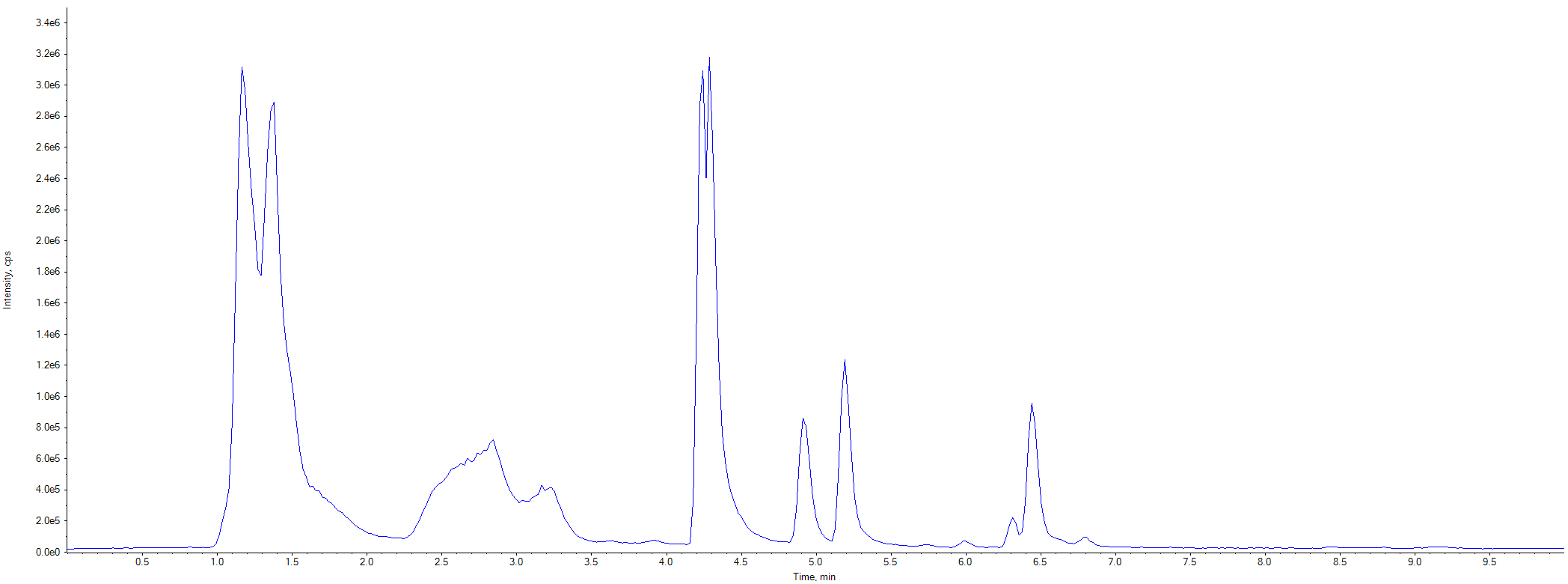


#### **Sample Name:** A_6 **Vial #:** 45

####
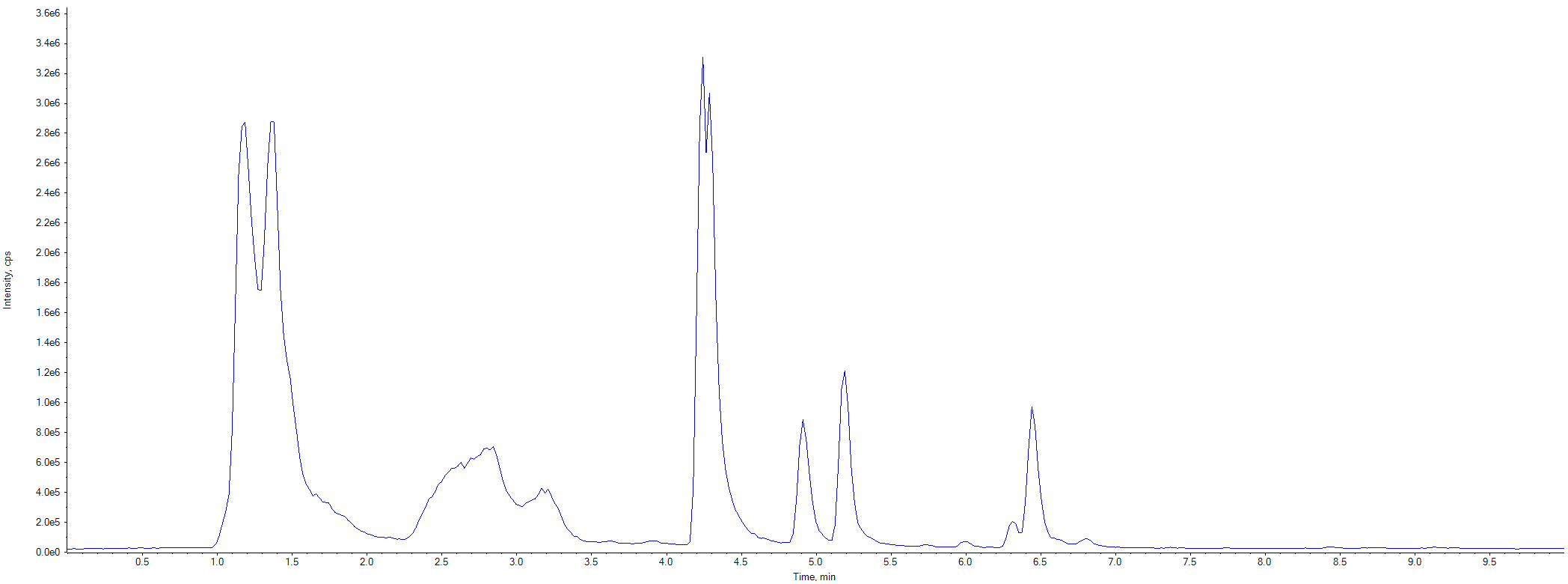


#### **Sample Name:** B_1 **Vial #:** 46

####
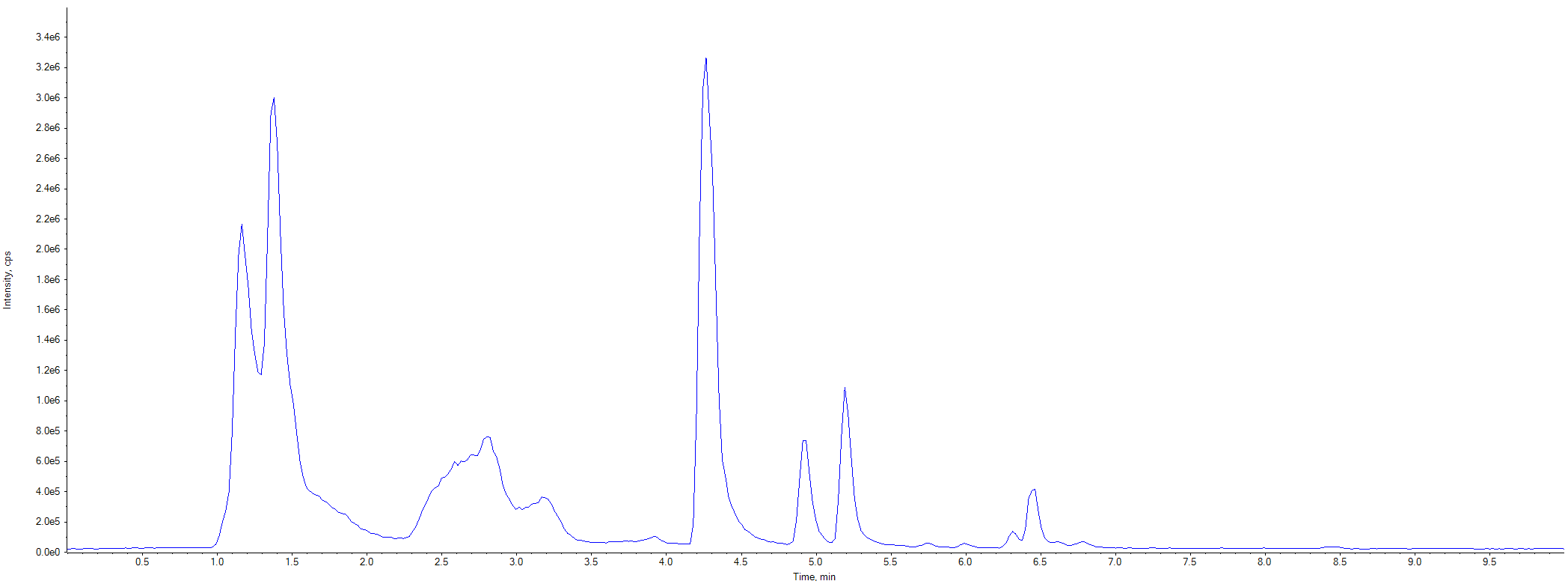


#### **Sample Name:** B_2 **Vial #:** 47

####
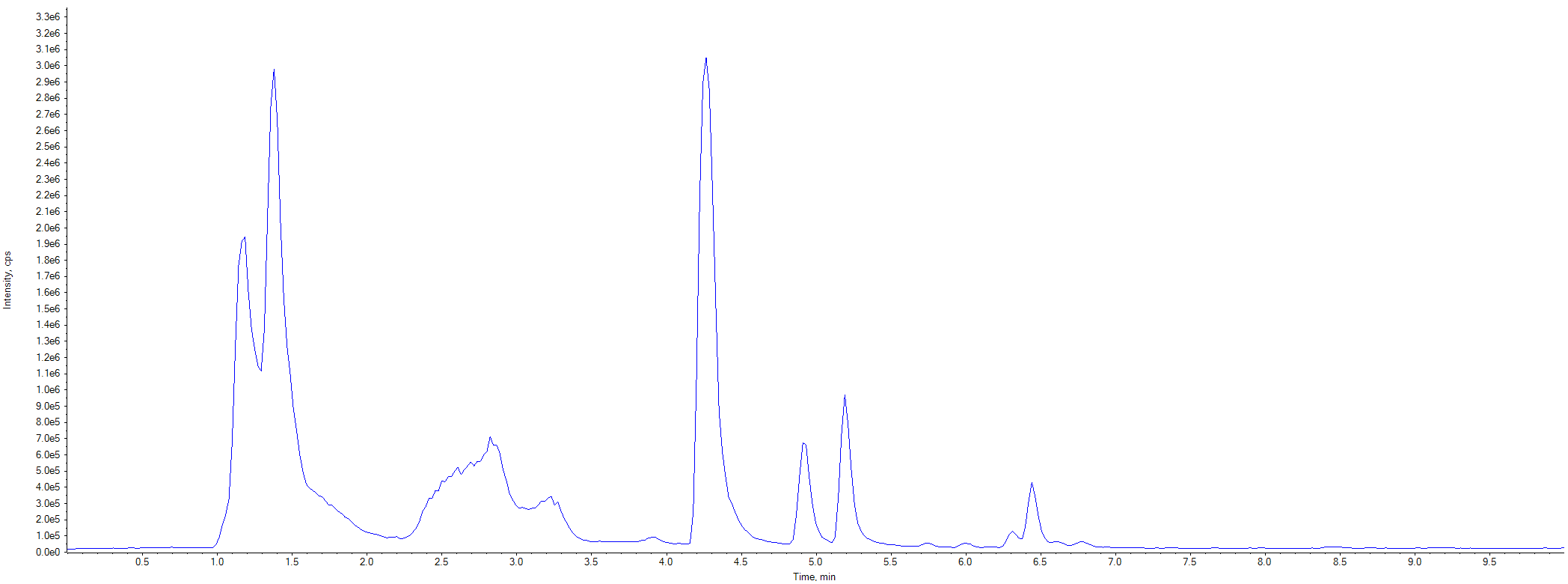


#### **Sample Name:** B_3 **Vial #:** 48

####
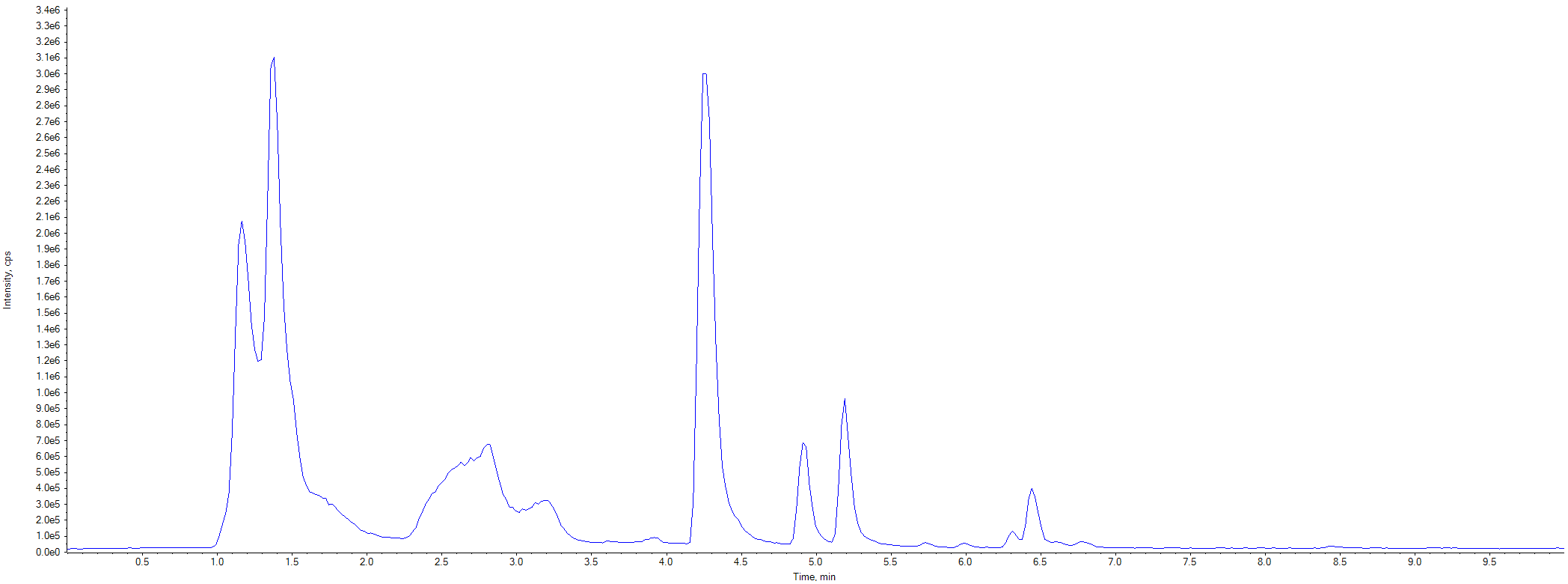


#### **Sample Name:** B_4 **Vial #:** 1

####
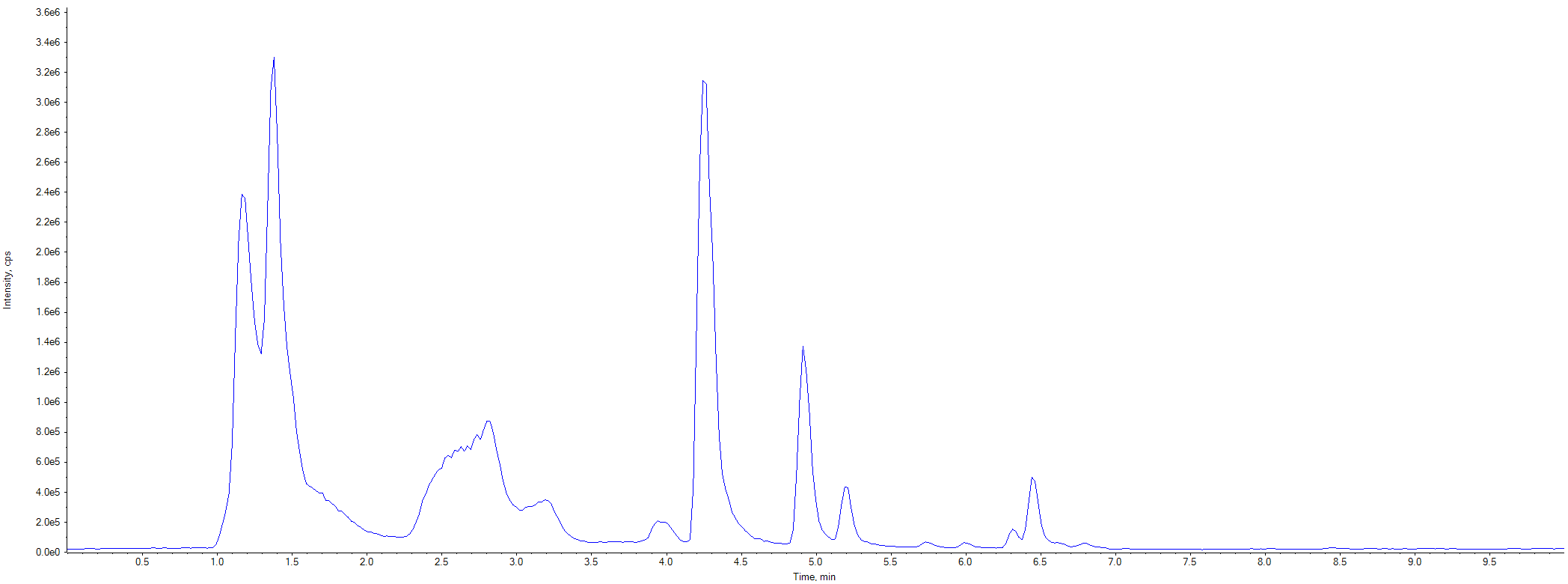


#### **Sample Name:** B_5 **Vial #:** 2

####
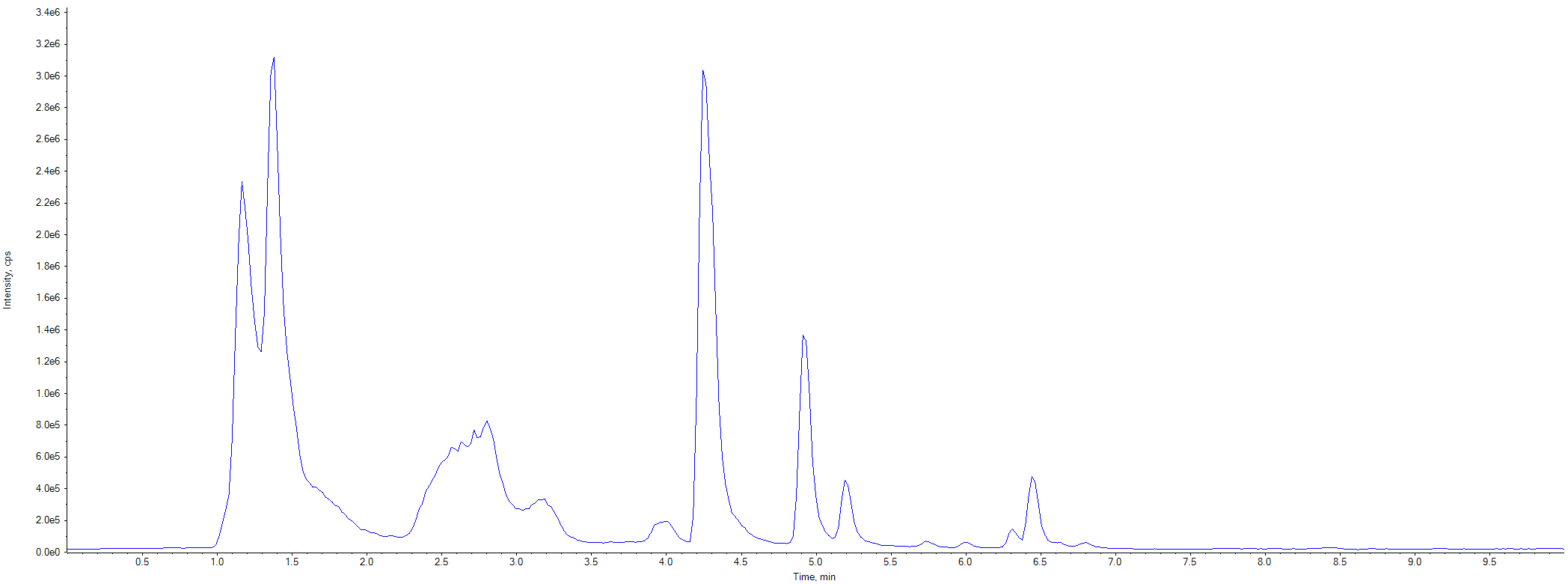


#### **Sample Name:** B_6 **Vial #:** 3

####
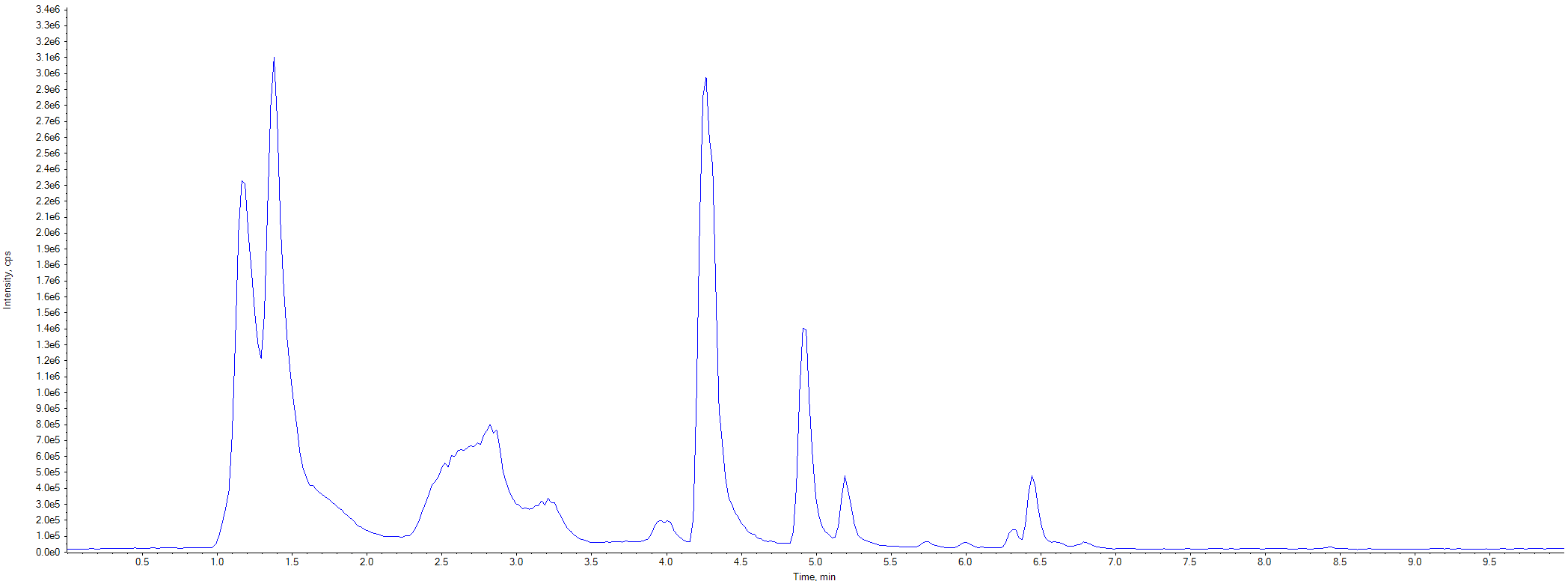


#### **Sample Name:** C_1 **Vial #:** 4

####
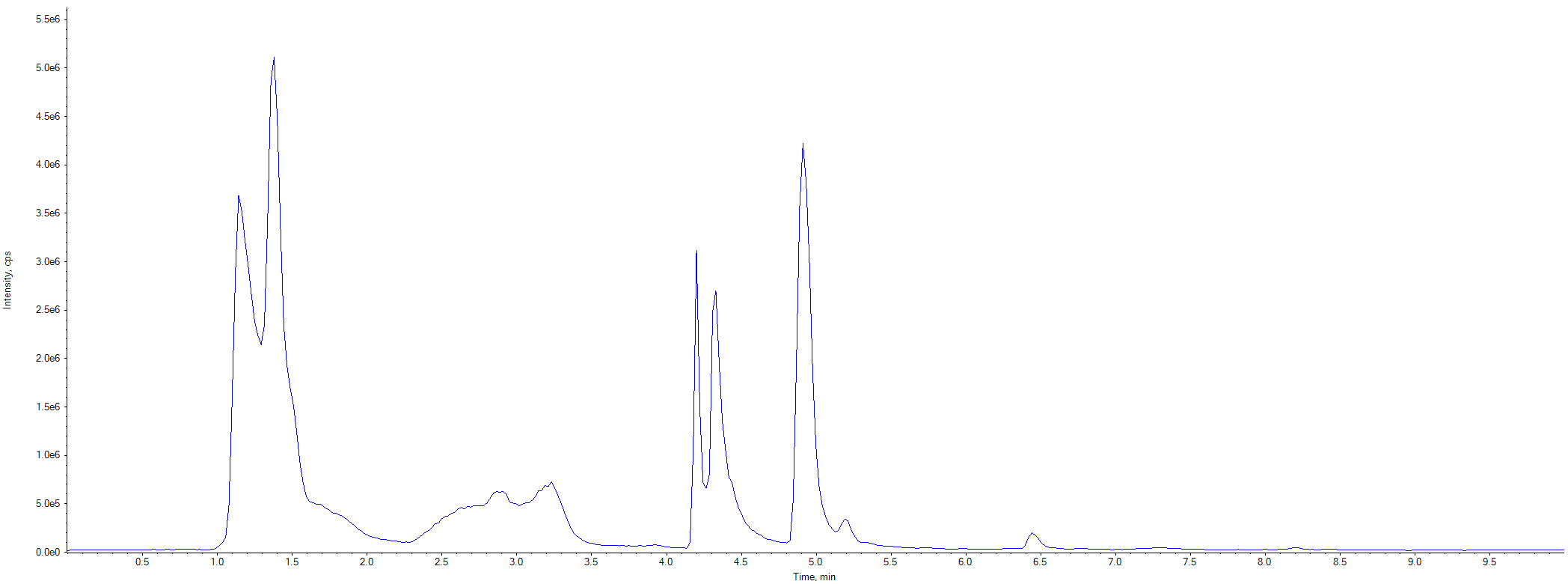


#### **Sample Name:** C_2 **Vial #:** 5

####
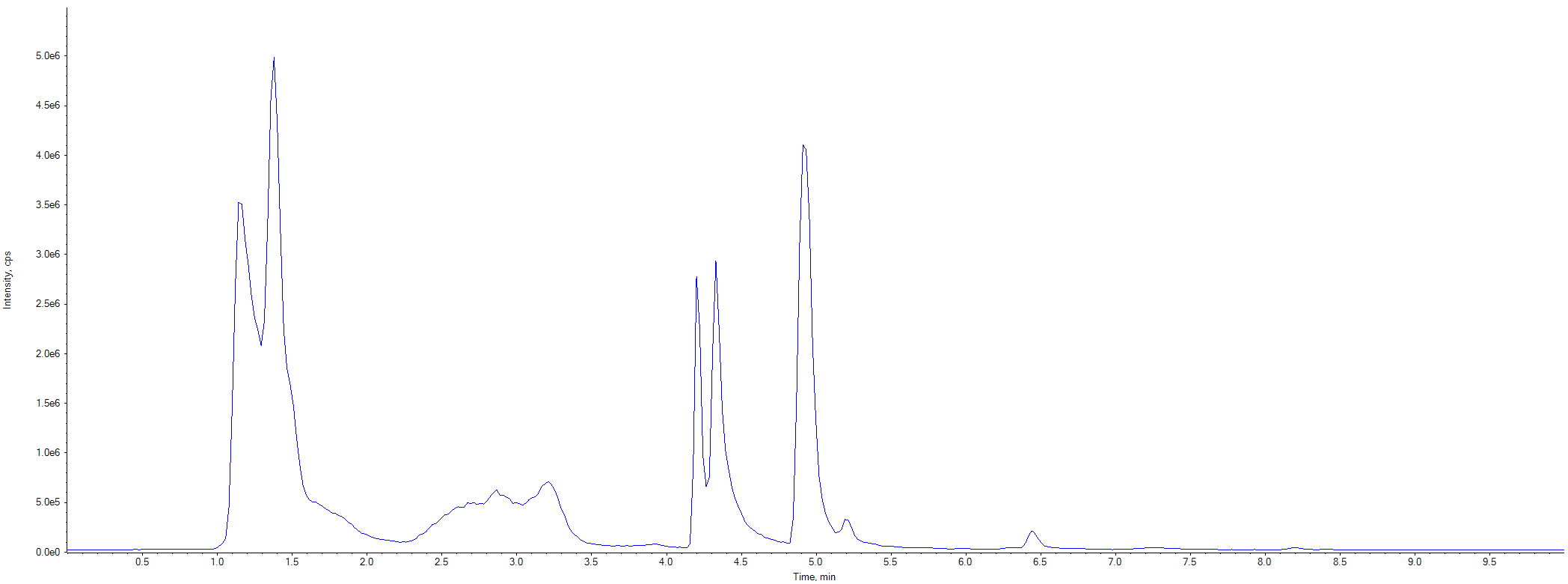


#### **Sample Name:** C_3 **Vial #:** 6

####
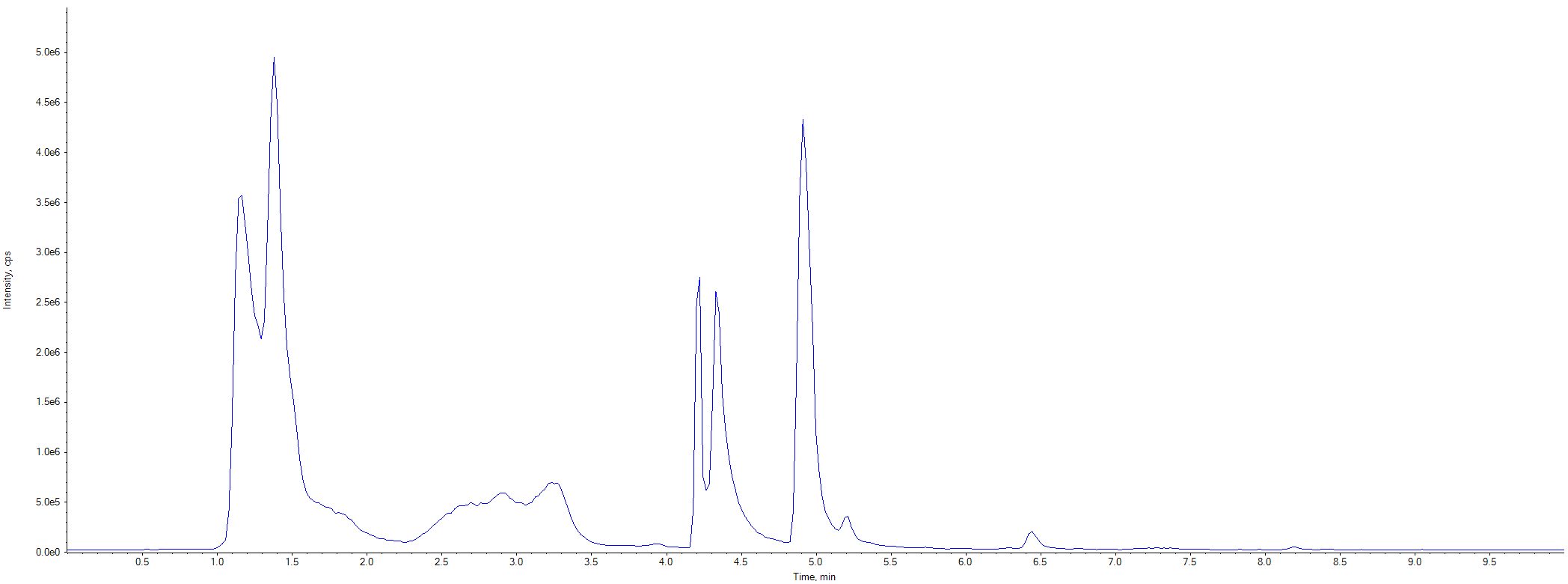


#### **Sample Name:** C_4 **Vial #:** 7

####
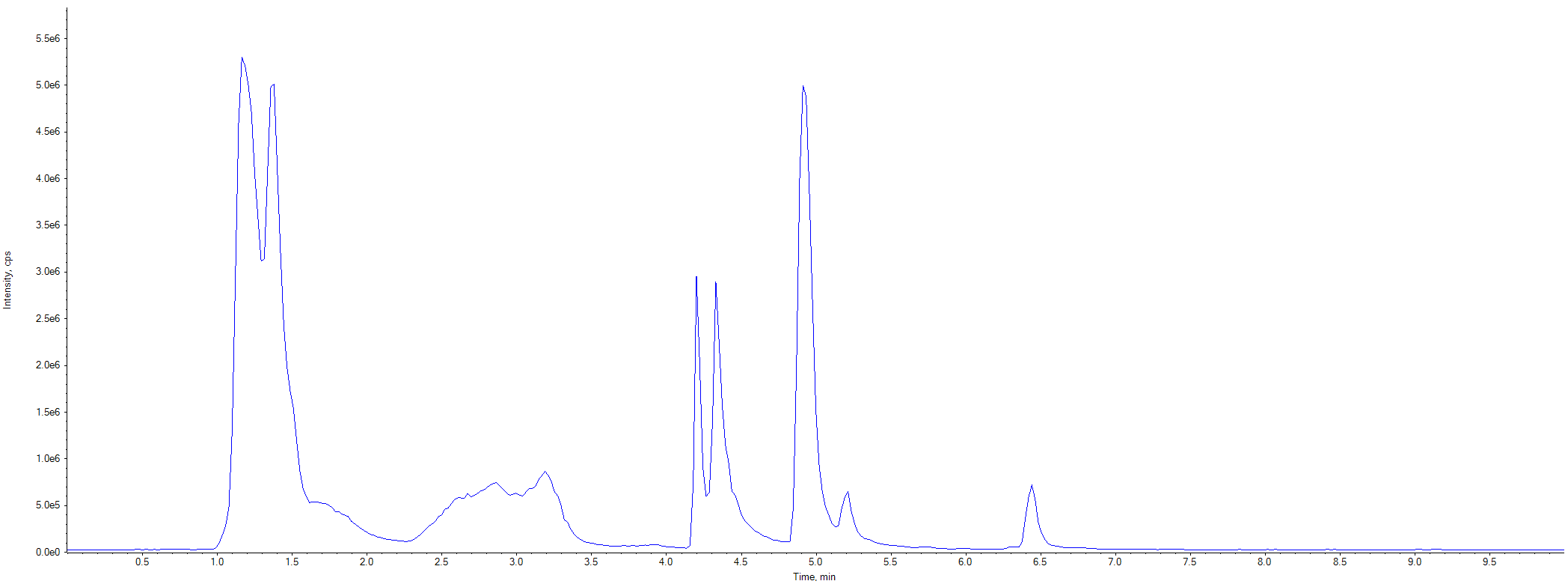


#### **Sample Name:** C_5 **Vial #:** 8

####
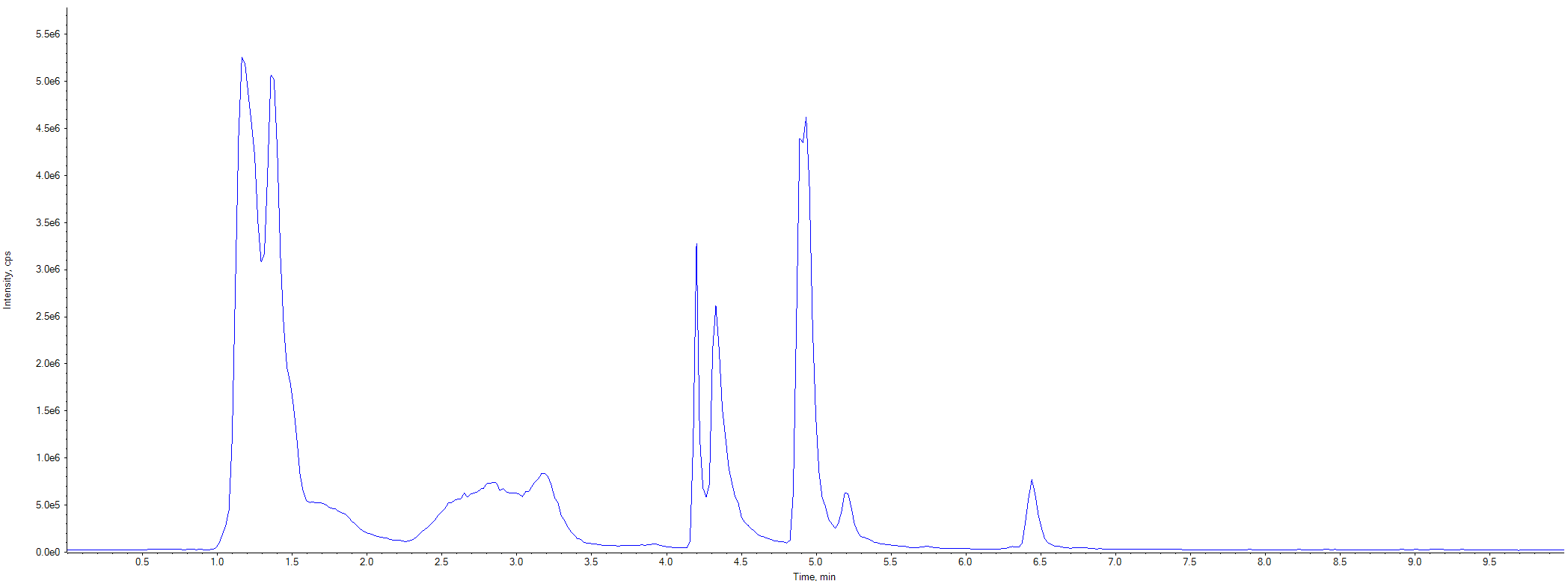


#### **Sample Name:** C_6 **Vial #:** 9

####
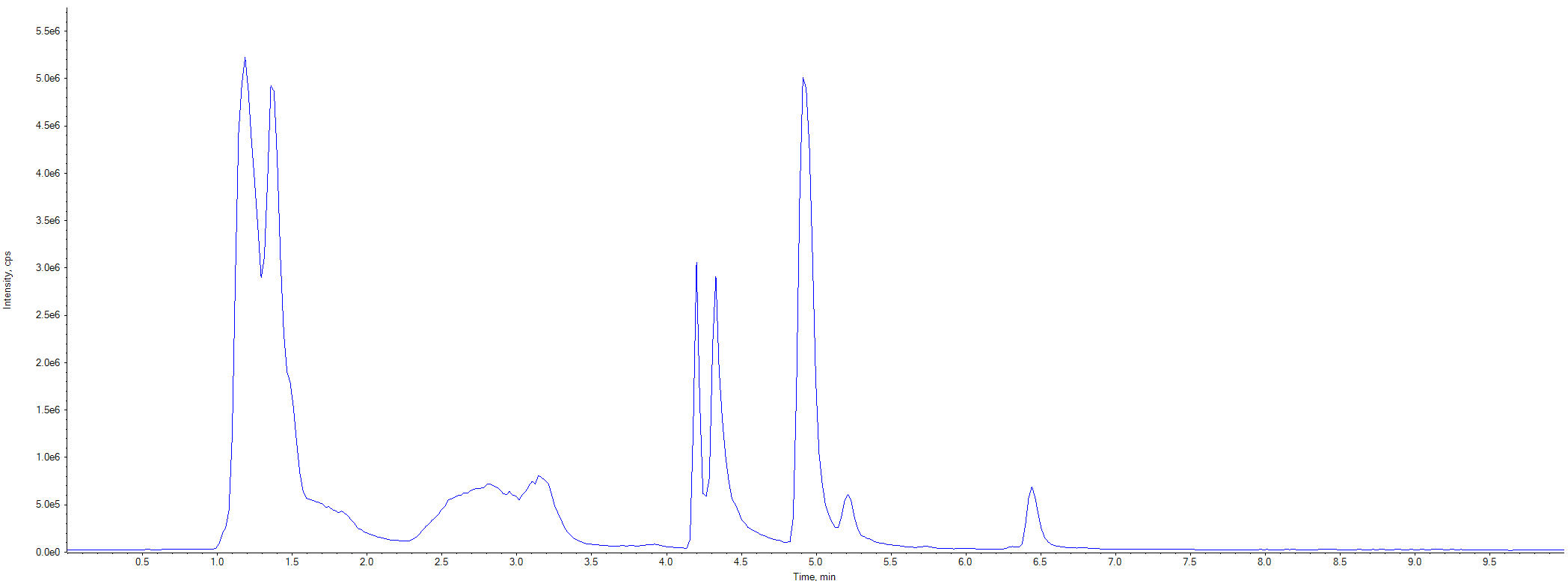


#### **Sample Name:** QC **Vial #:** 21

####
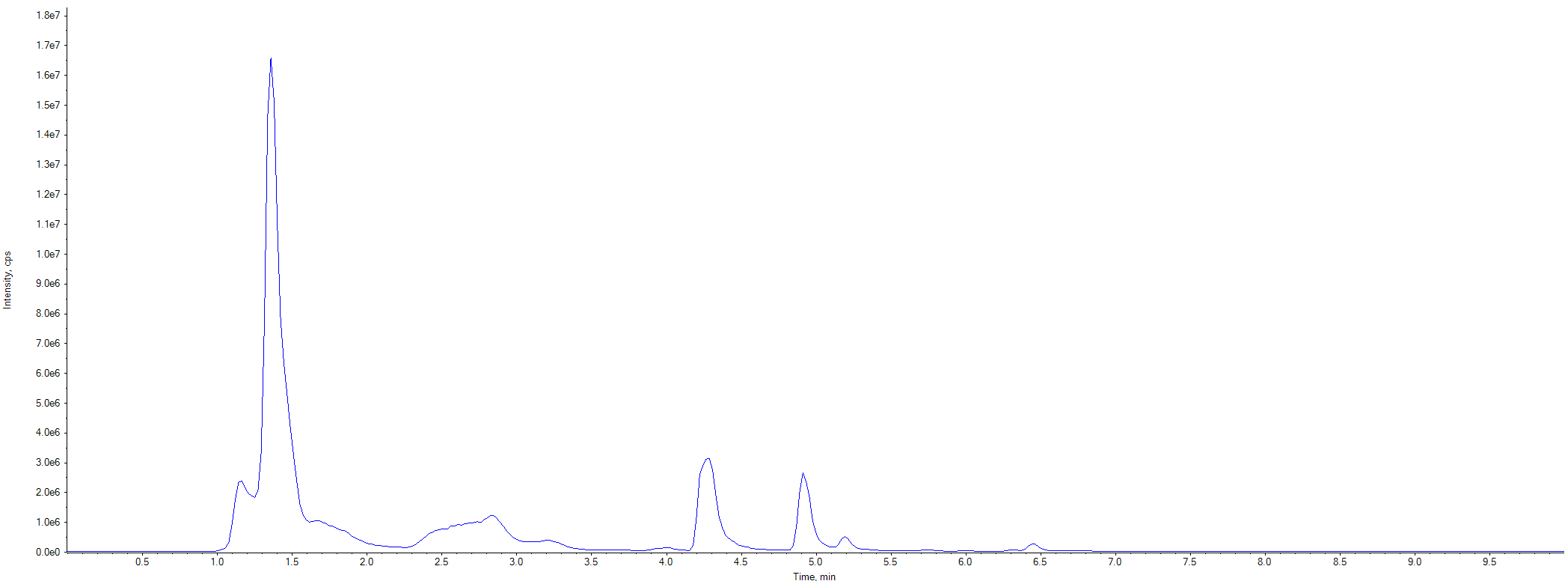


#### **Sample Name:** QC **Vial #:** 21

####
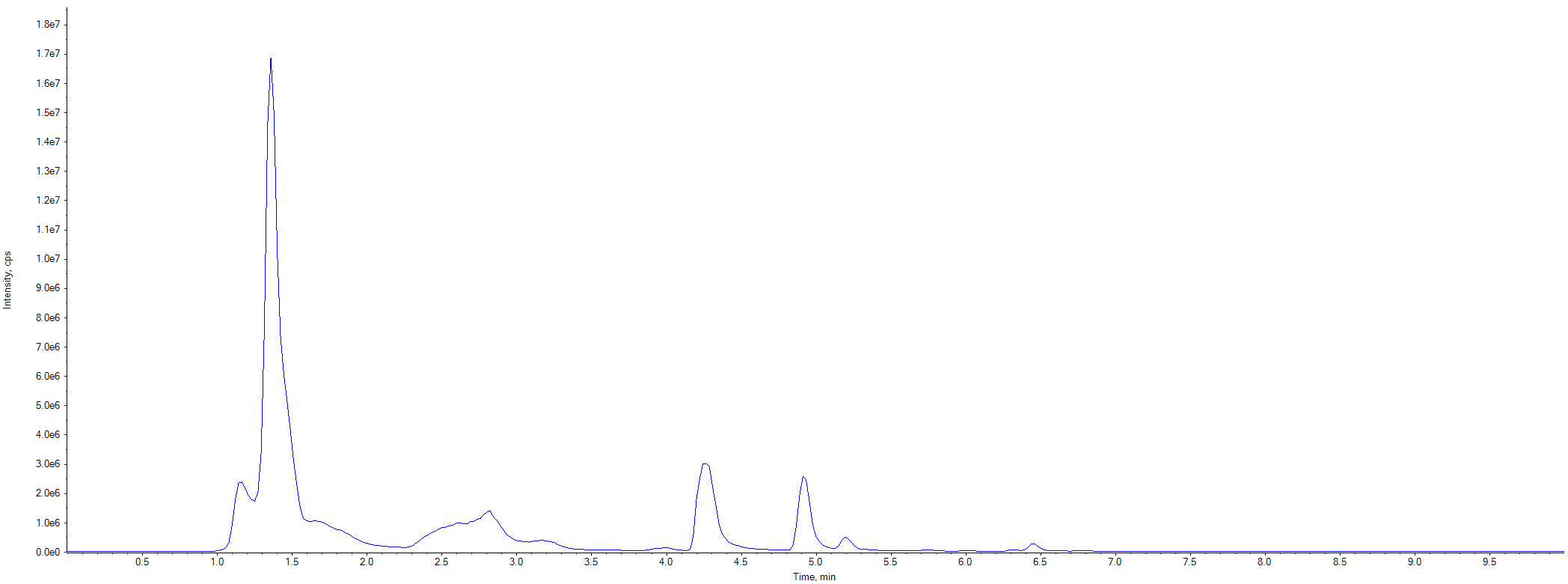


#### **Sample Name:** QC **Vial #:** 21

####
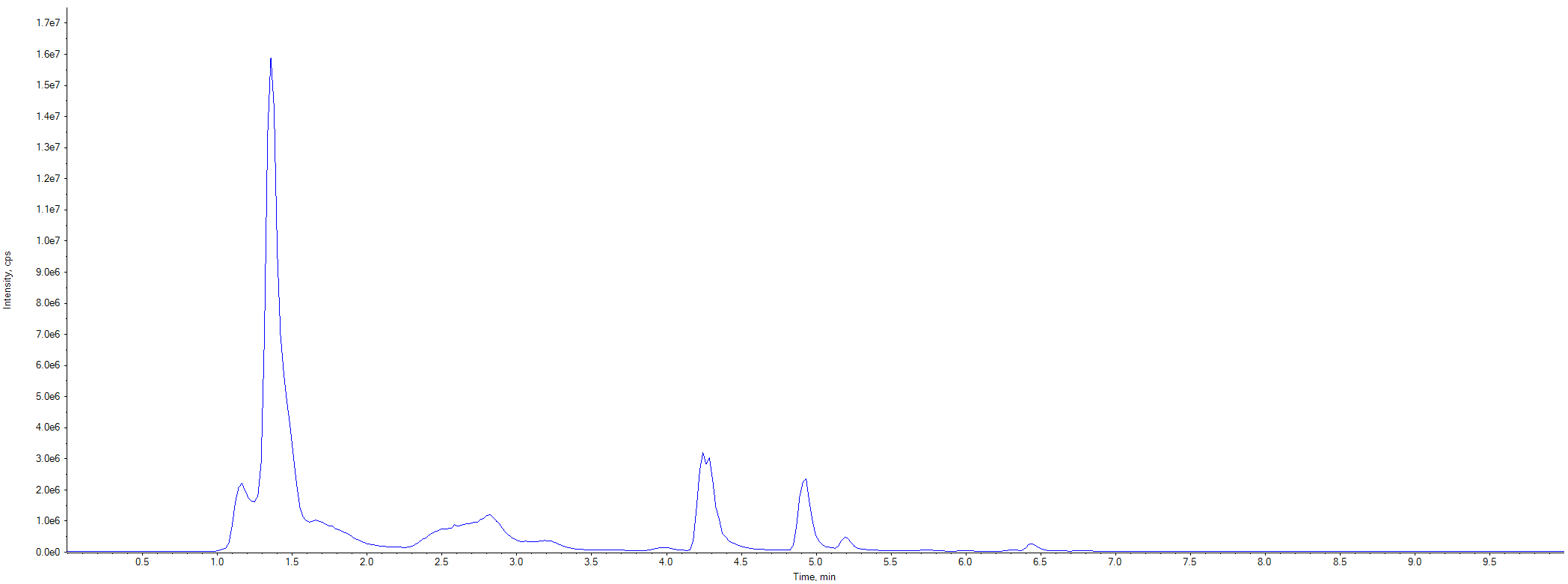


#### **Sample Name:** QC **Vial #:** 21

####
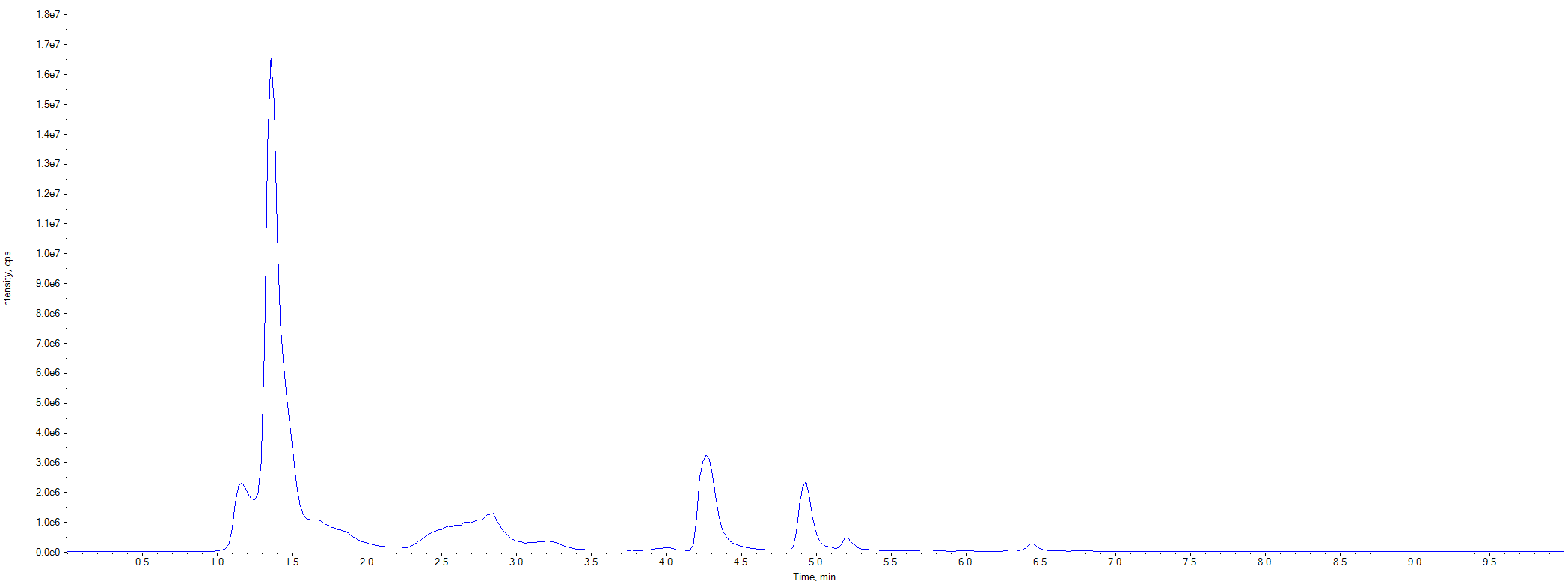


#### **Sample Name:** A_1_100 **Vial #:** 3

####
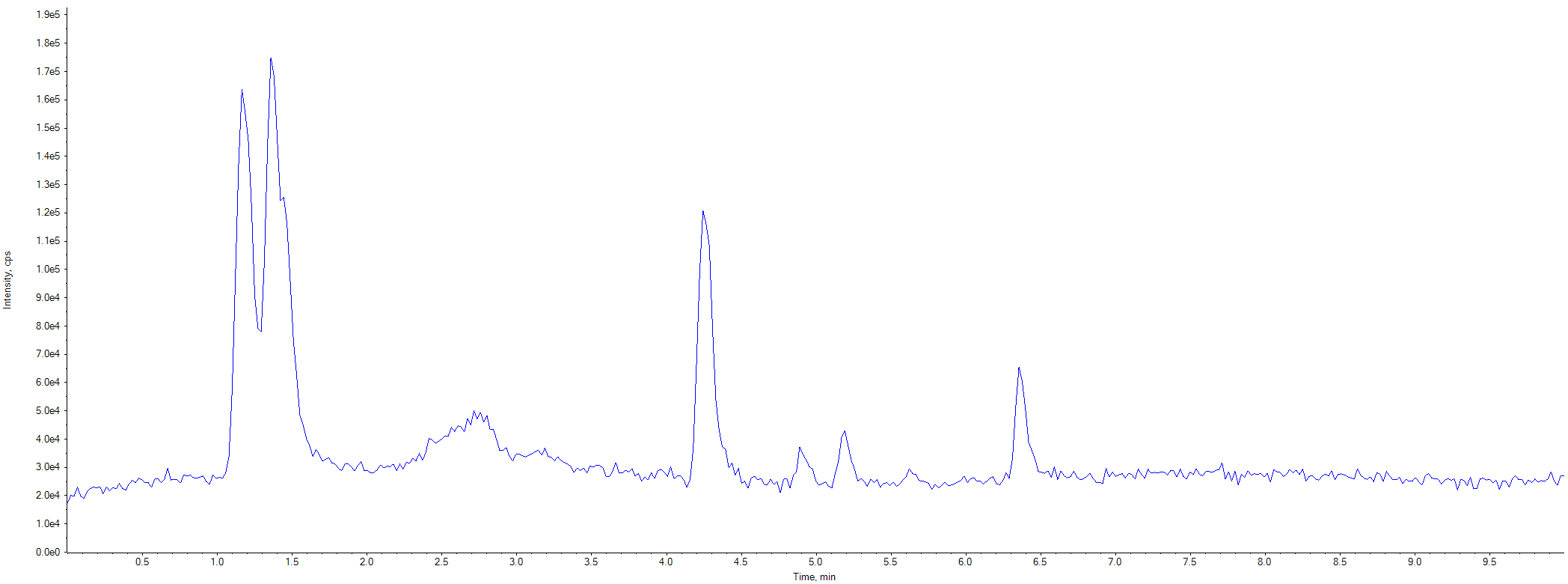


#### **Sample Name:** A_2_100 **Vial #:** 4

####
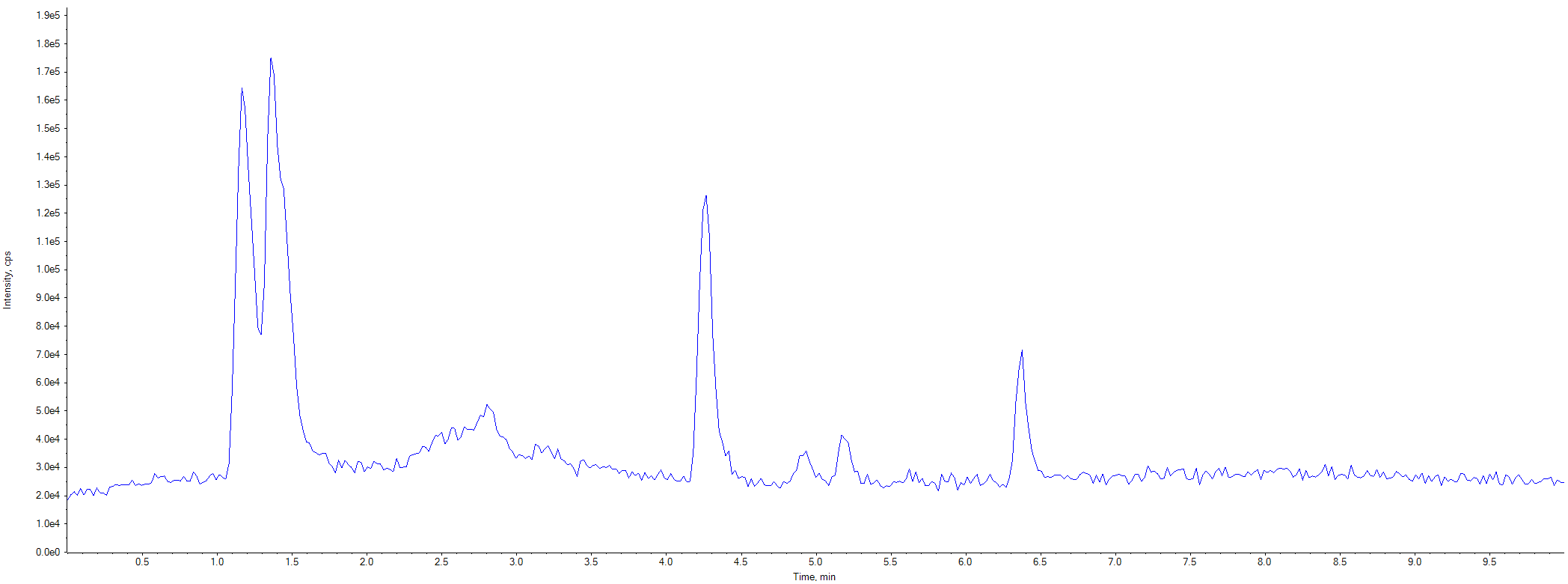


#### **Sample Name:** A_3_100 **Vial #:** 5

####
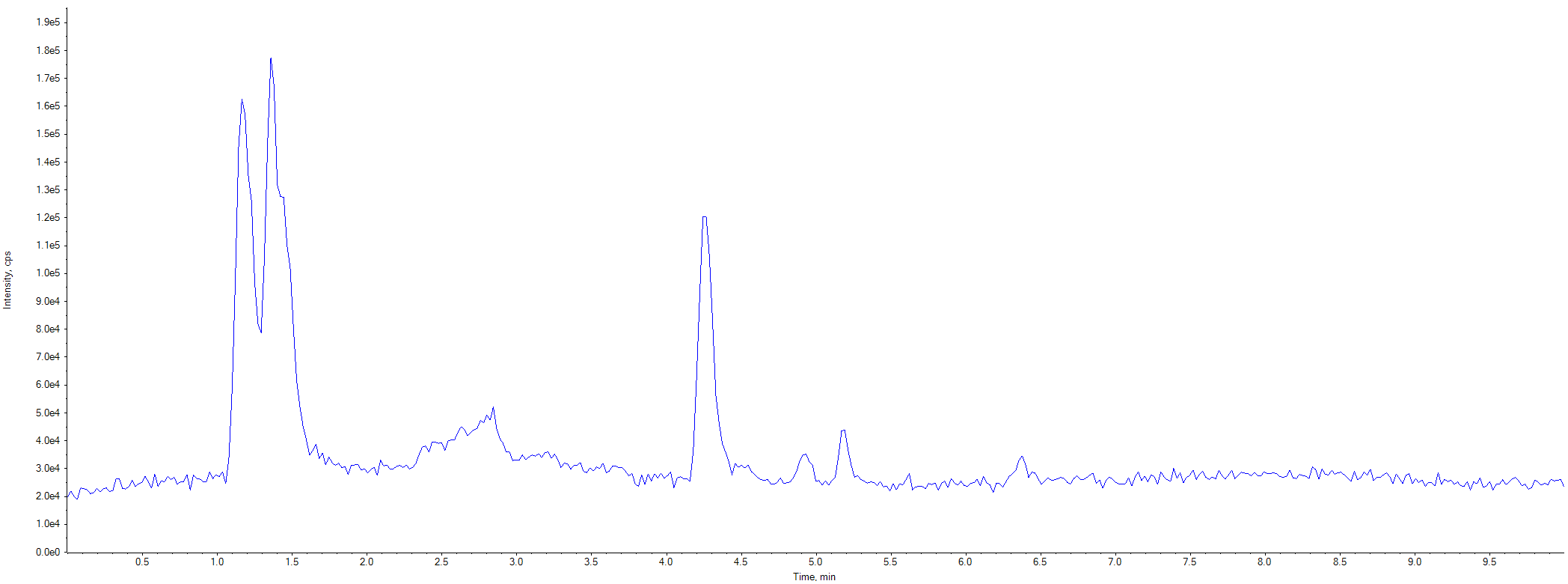


#### **Sample Name:** A_4_100 **Vial #:** 6

####
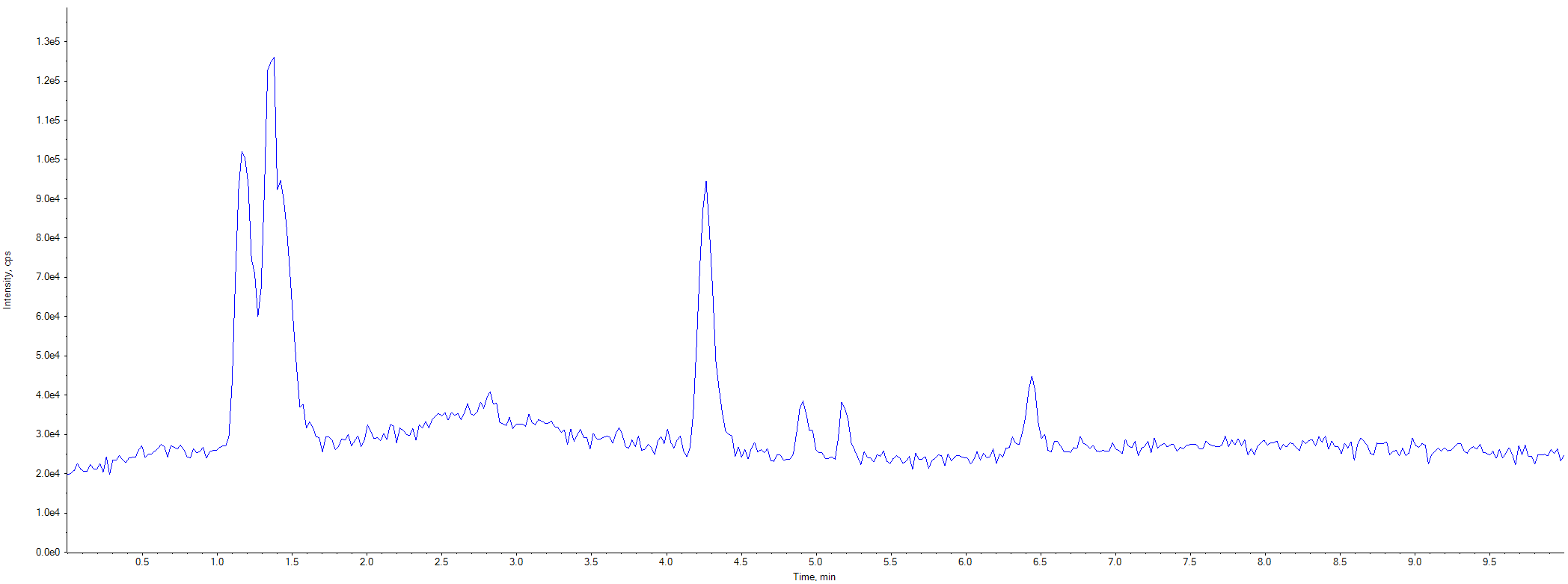


#### **Sample Name:** A_5_100 **Vial #:** 7

####
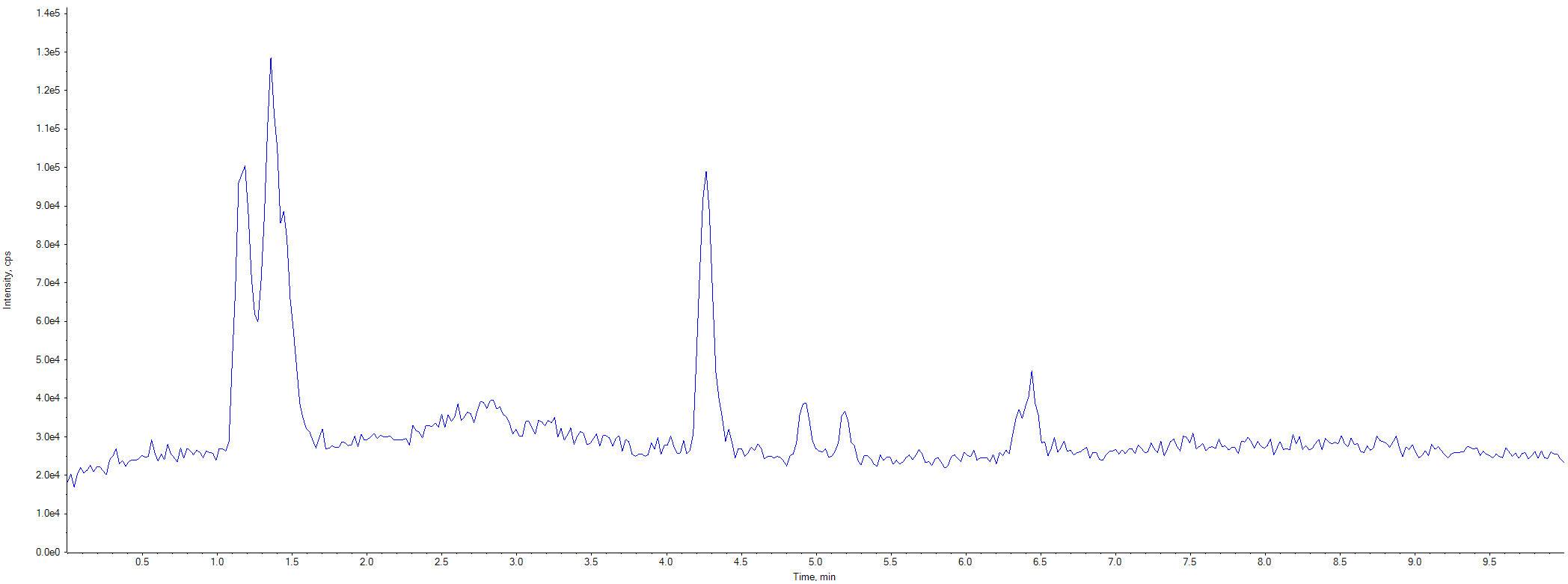


#### **Sample Name:** A_6_100 **Vial #:** 8

####
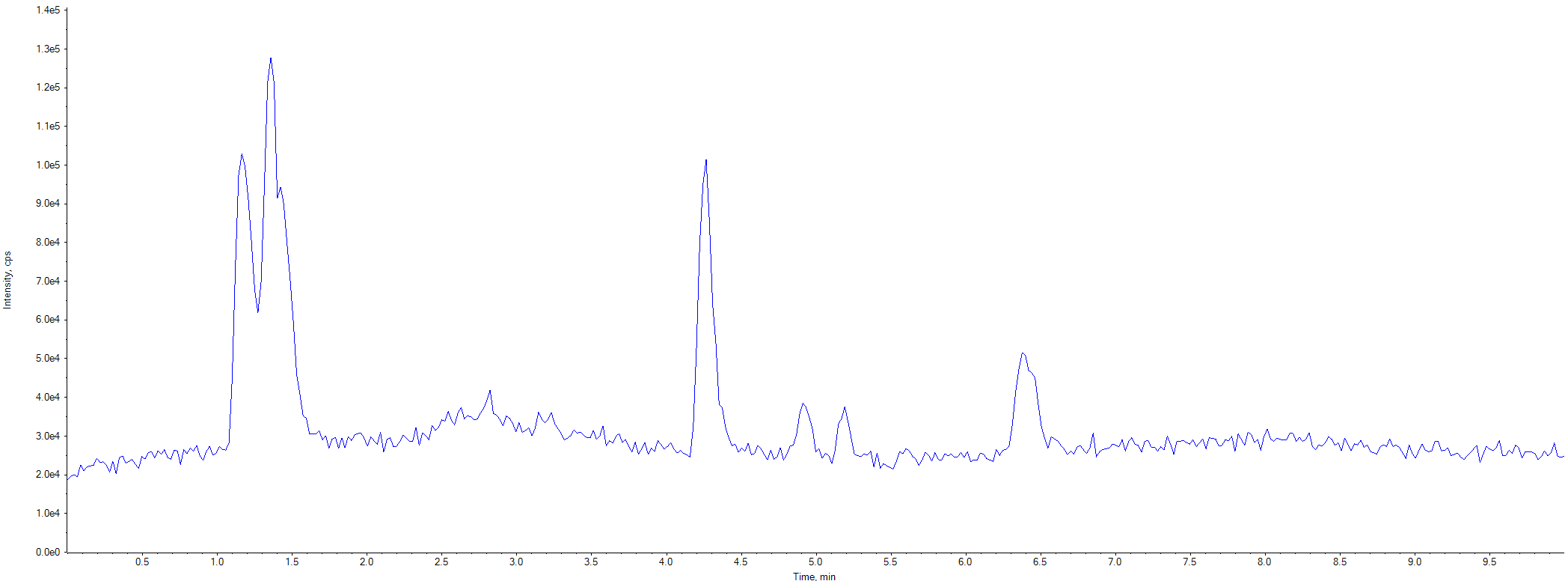


#### **Sample Name:** B_1_100 **Vial #:** 9

####
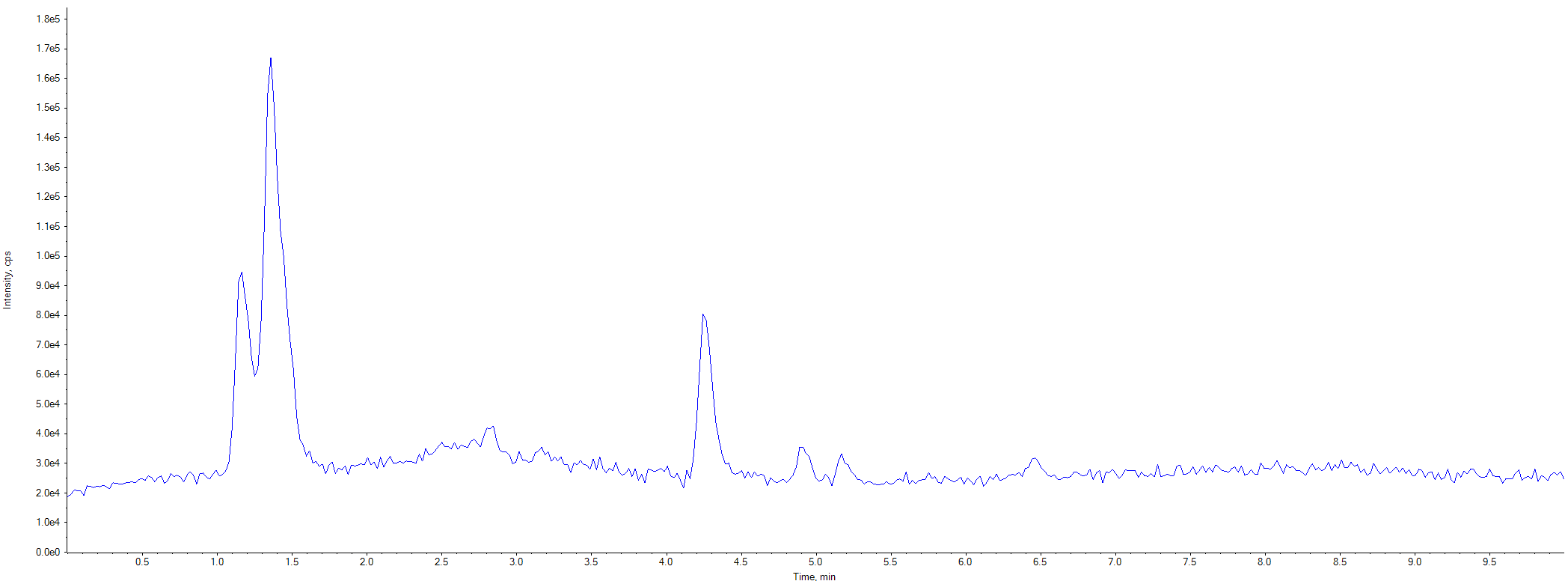


#### **Sample Name:** B_2_100 **Vial #:** 10

####
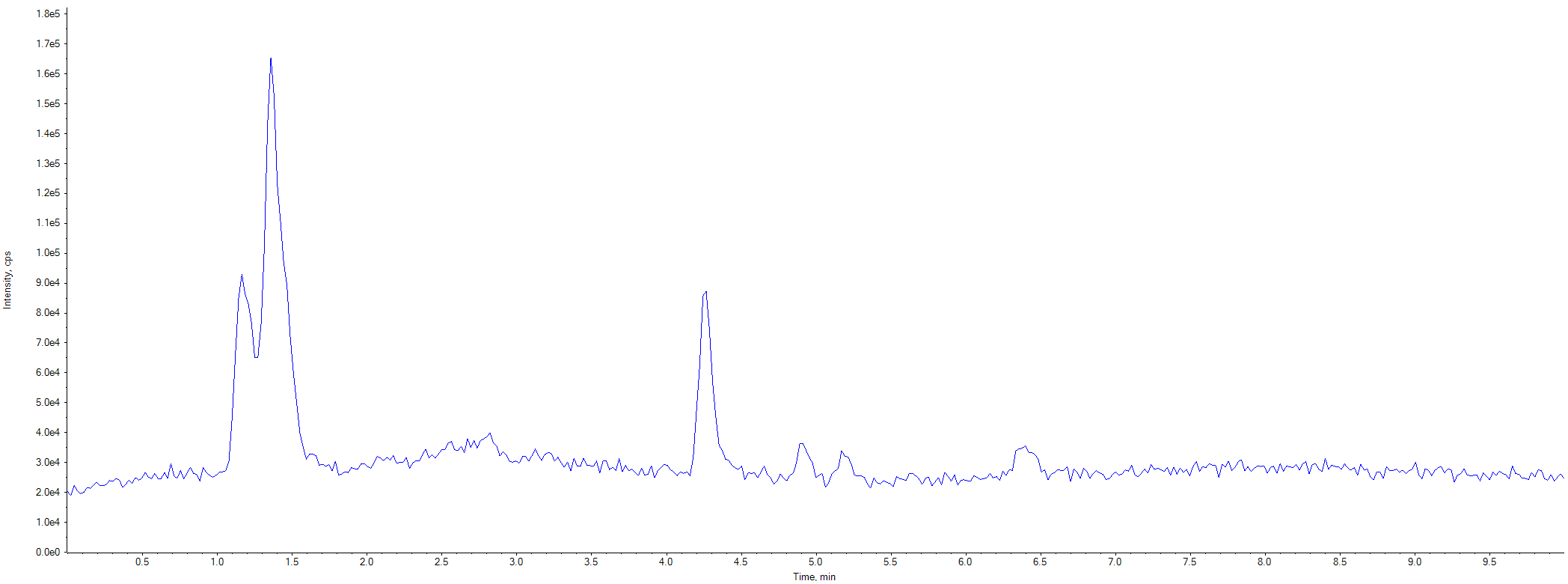


#### **Sample Name:** B_3_100 **Vial #:** 11

####
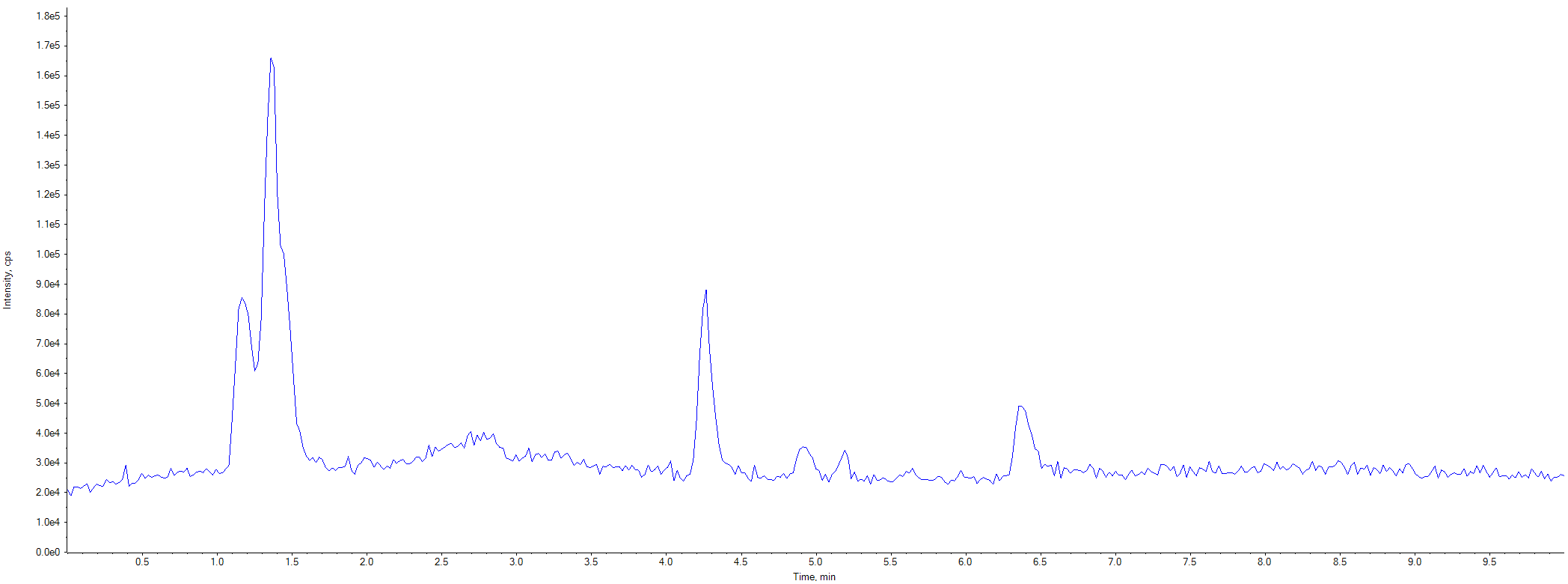


#### **Sample Name:** B_4_100 **Vial #:** 12

####
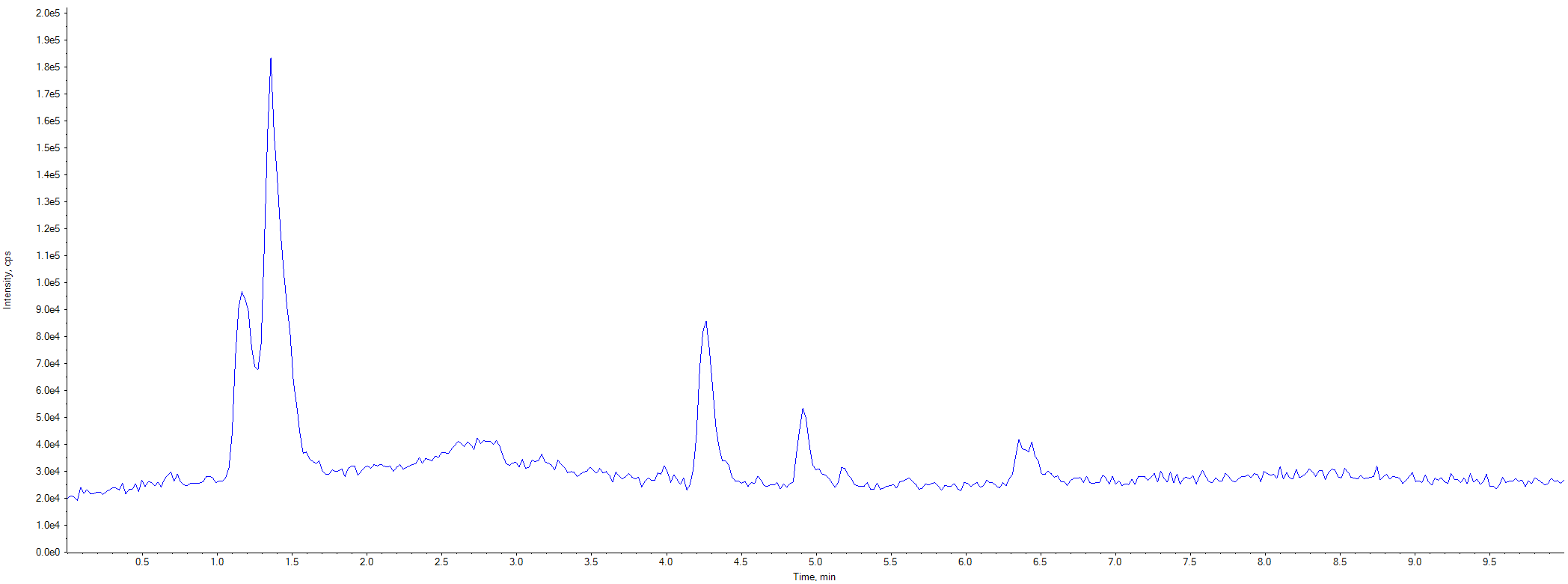


#### **Sample Name:** B_5_100 **Vial #:** 13

####
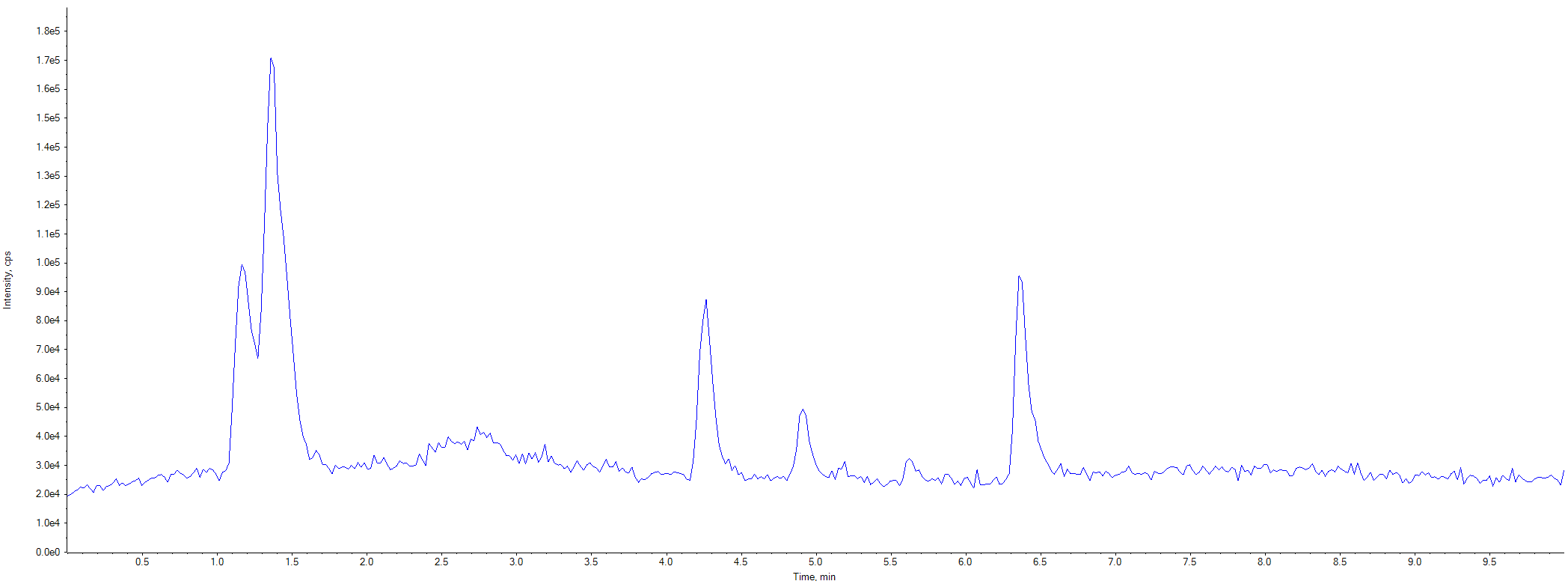


#### **Sample Name:** B_6_100 **Vial #:** 14

####
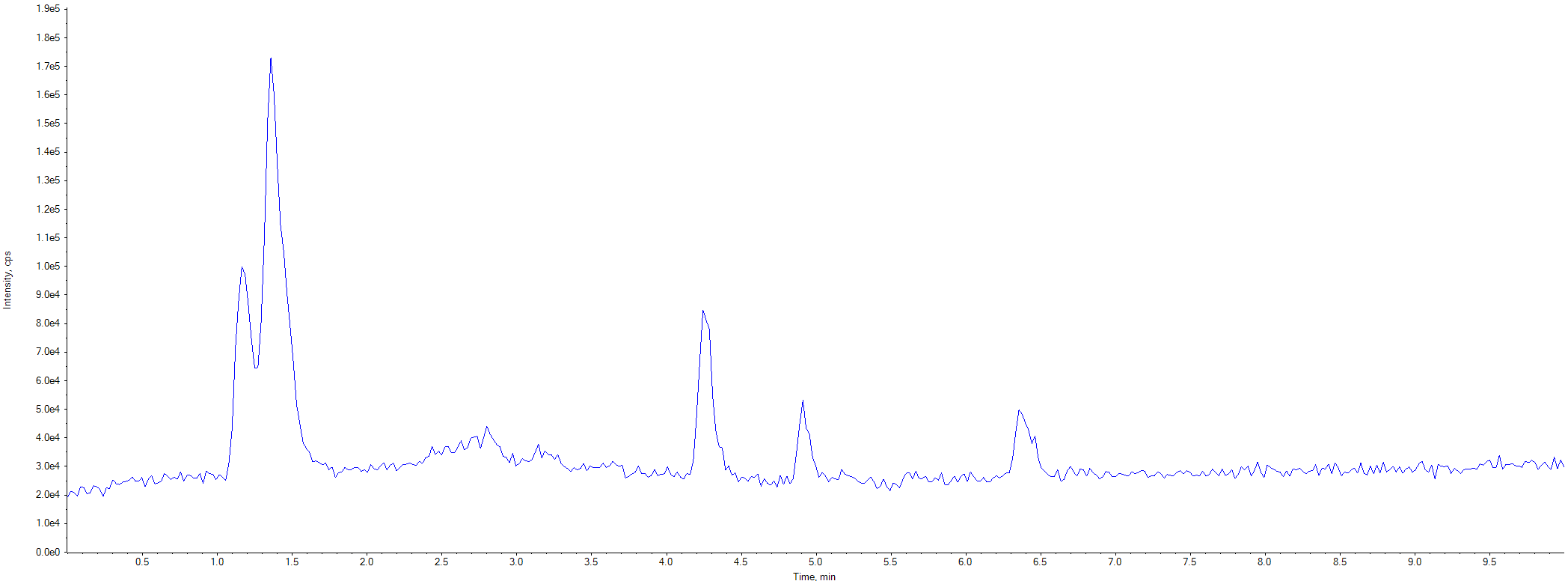


#### **Sample Name:** C_1_100 **Vial #:** 15

####
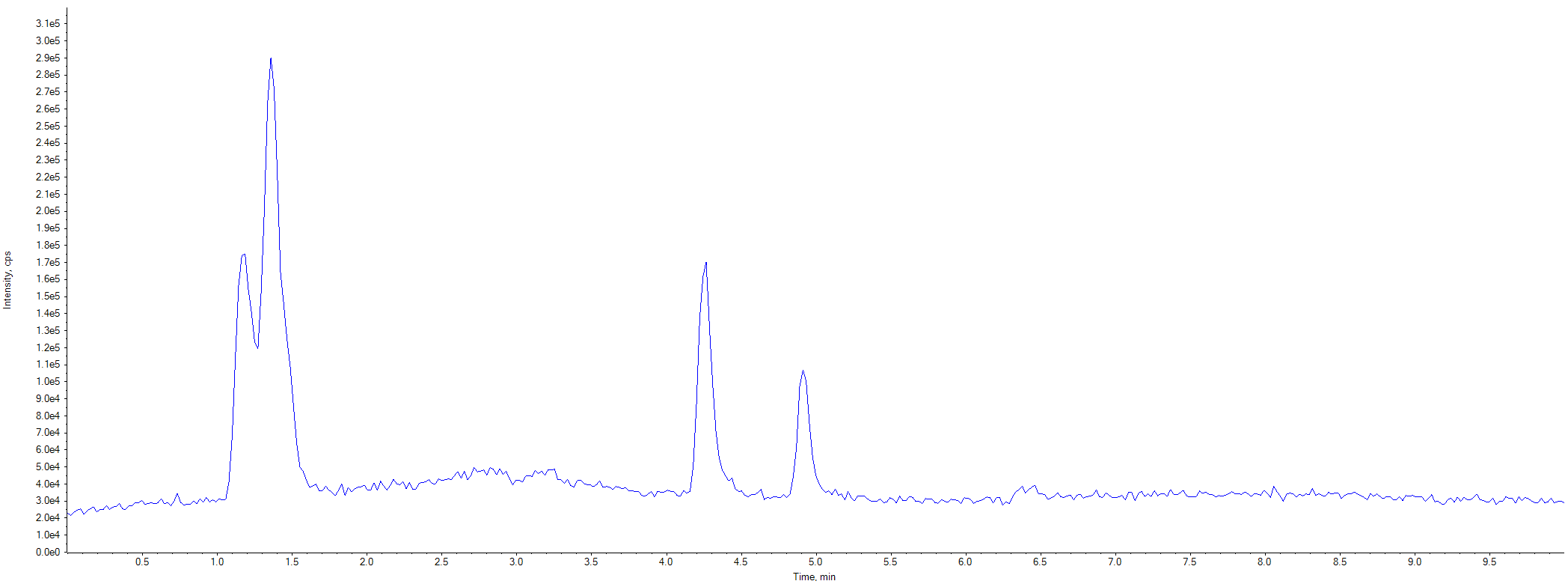


#### **Sample Name:** C_2_100 **Vial #:** 16

####
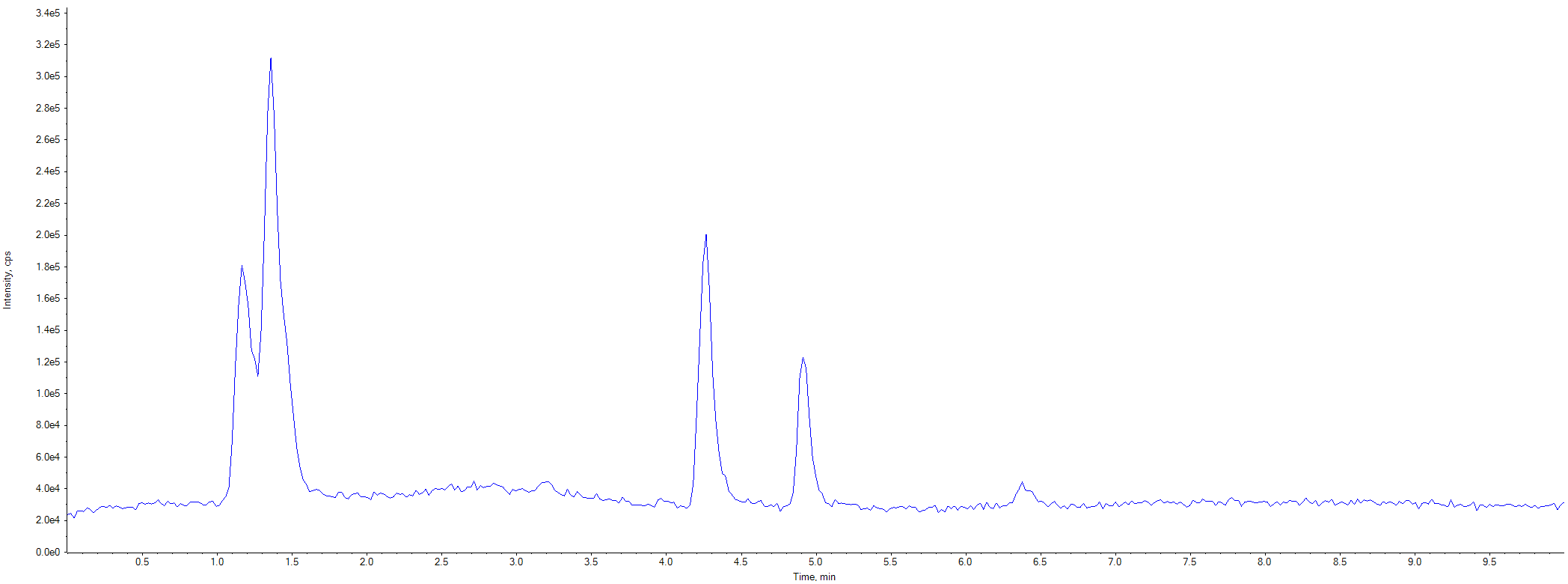


#### **Sample Name:** C_3_100 **Vial #:** 17

####
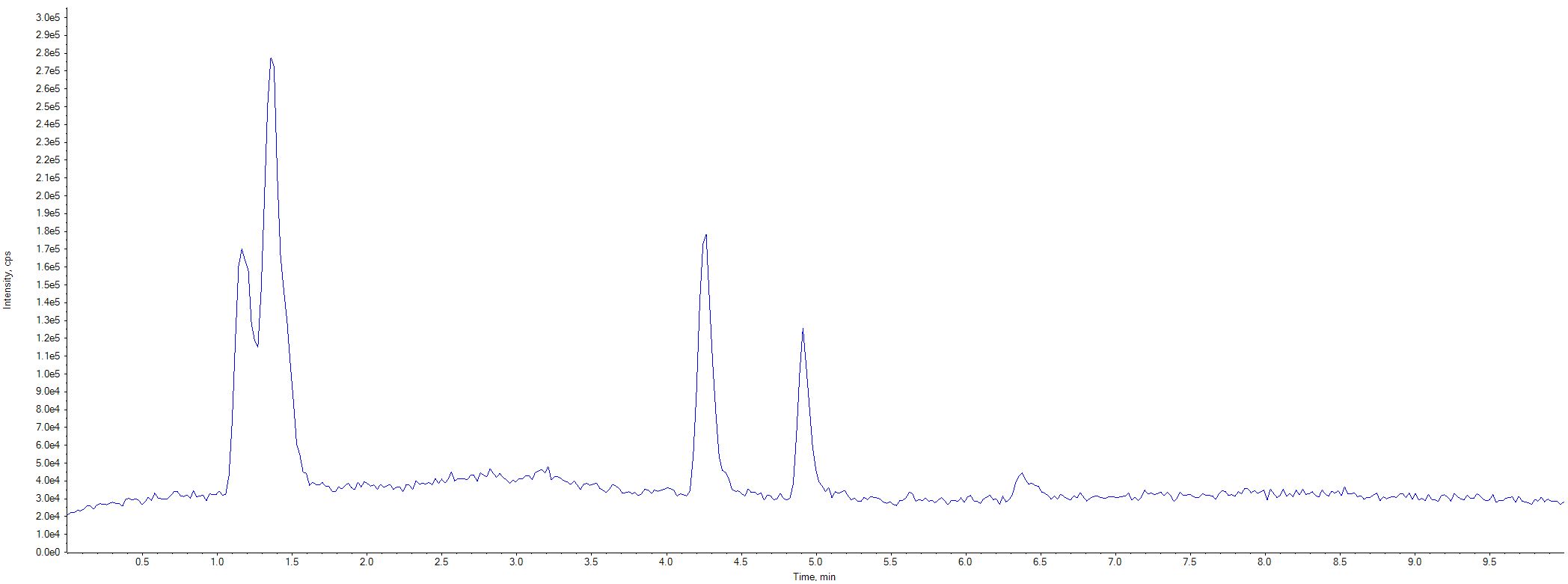


#### **Sample Name:** C_4_100 **Vial #:** 18

####
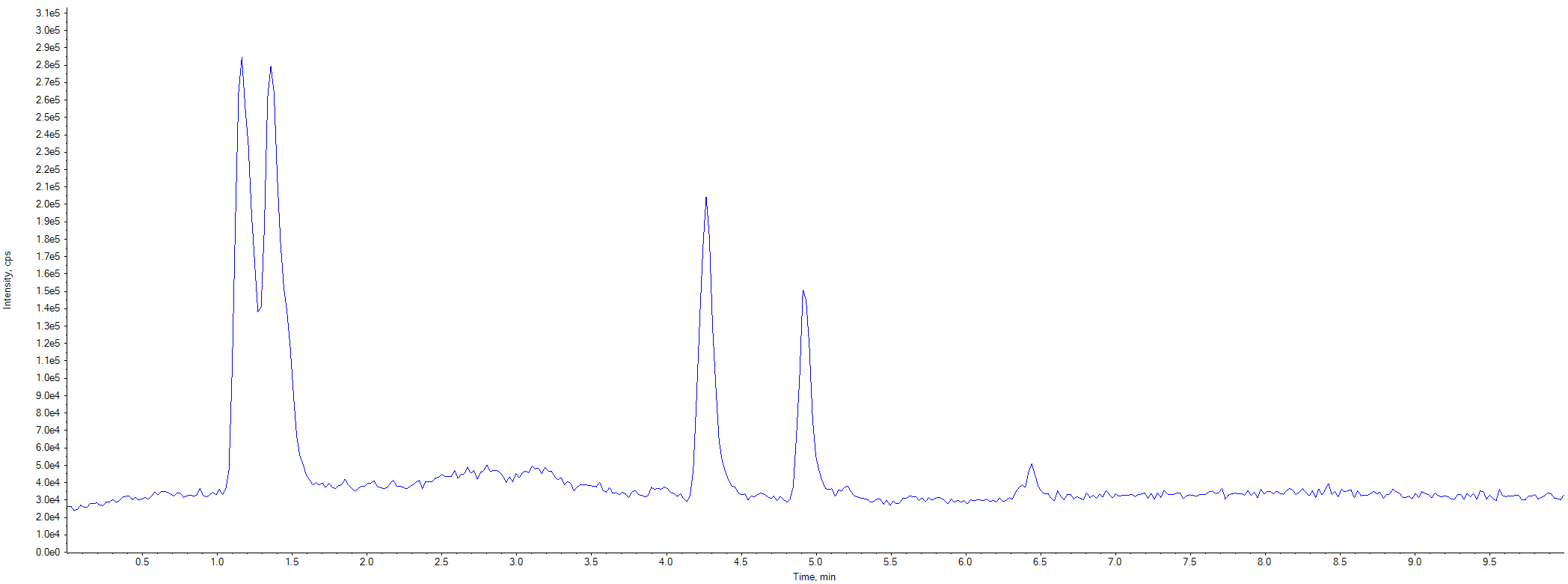


#### **Sample Name:** C_5_100 **Vial #:** 19

####
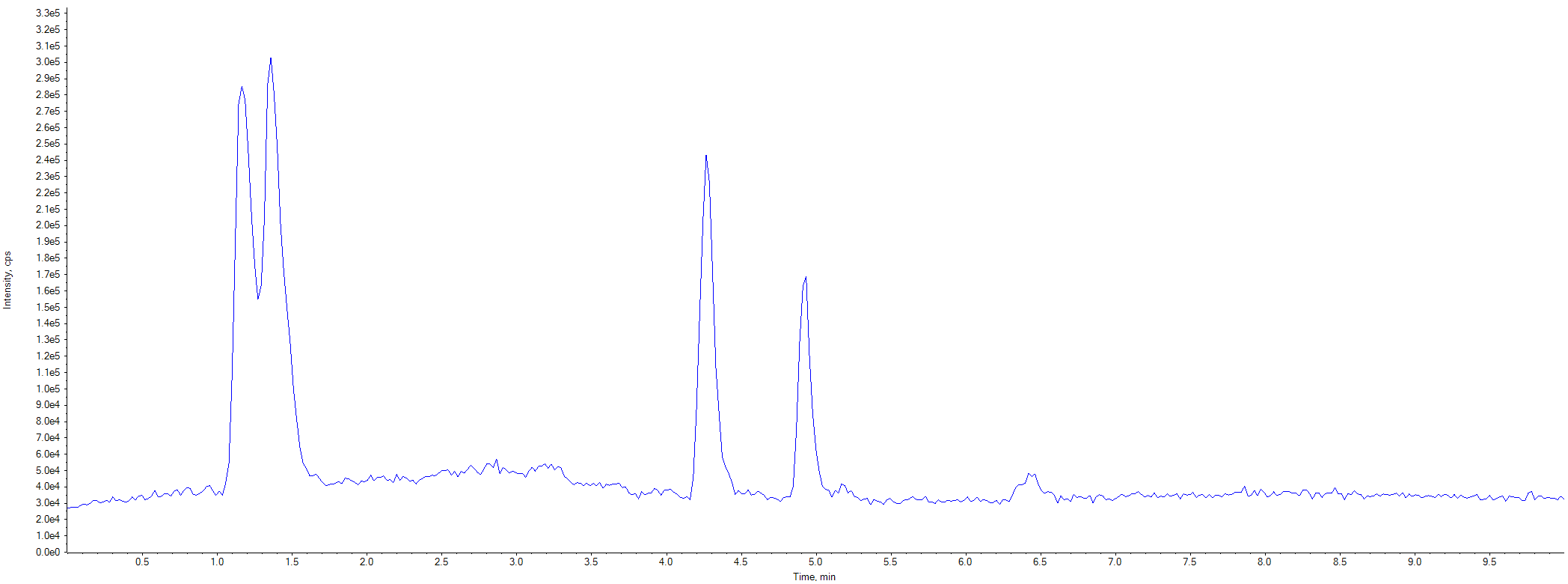


#### **Sample Name:** C_6_100 **Vial #:** 20

####
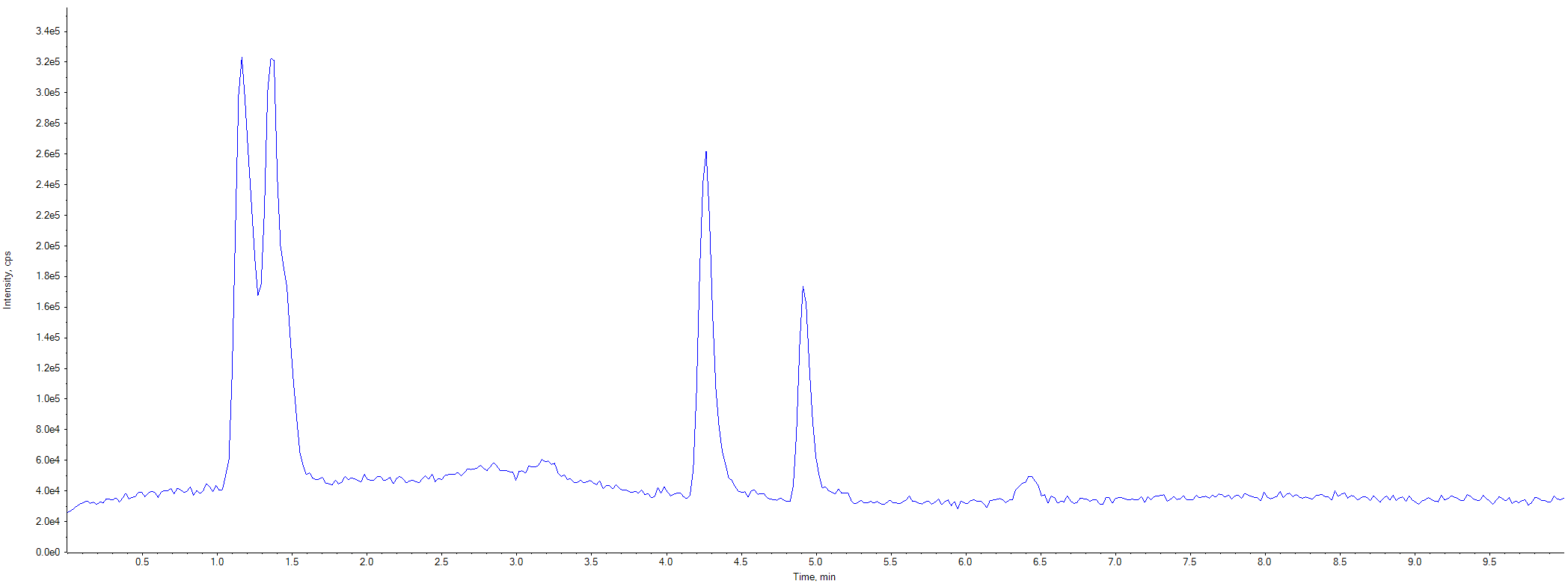


#### **Sample Name:** QC_100 **Vial #:** 31

####
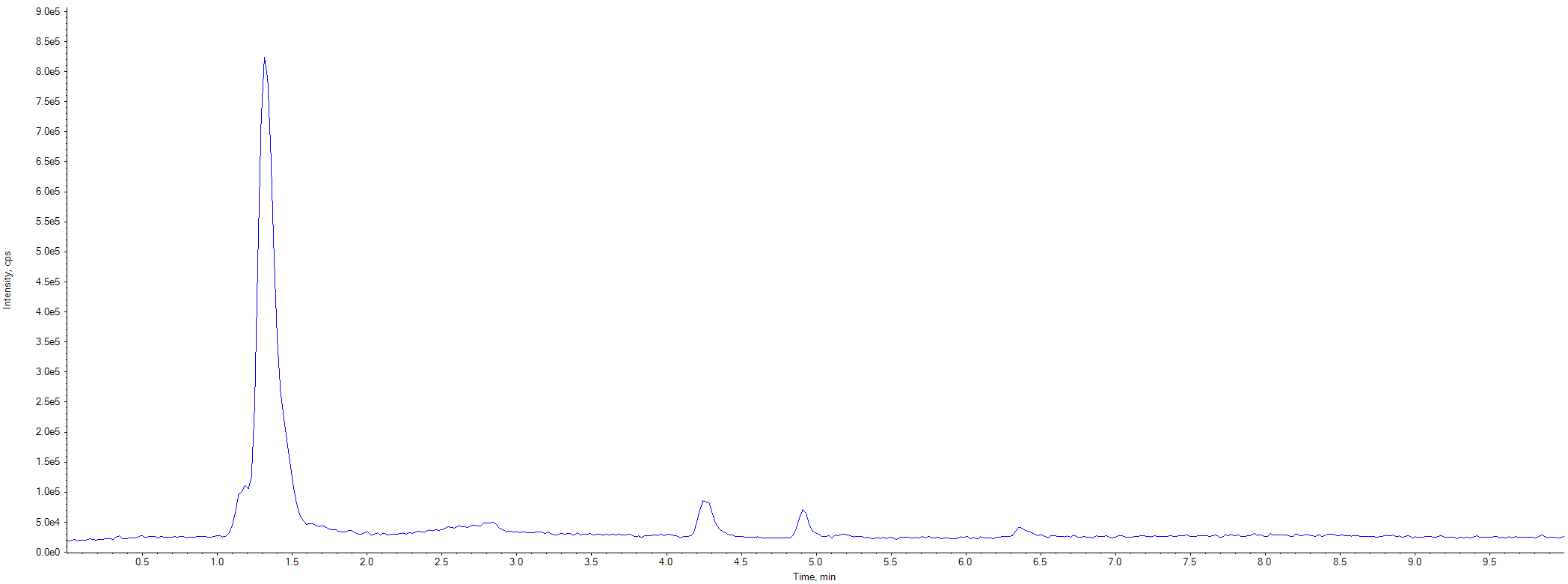


#### **Sample Name:** QC_100 **Vial #:** 31

####
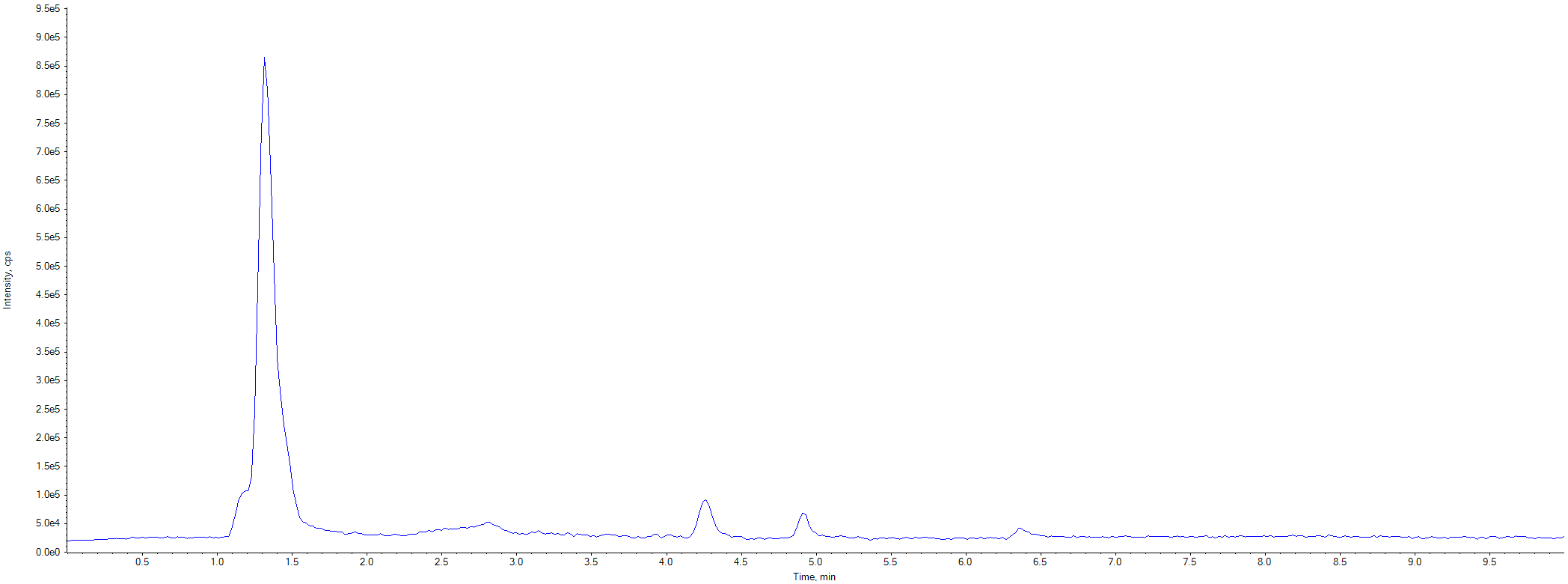


#### **Sample Name:** QC_100 **Vial #:** 31

####
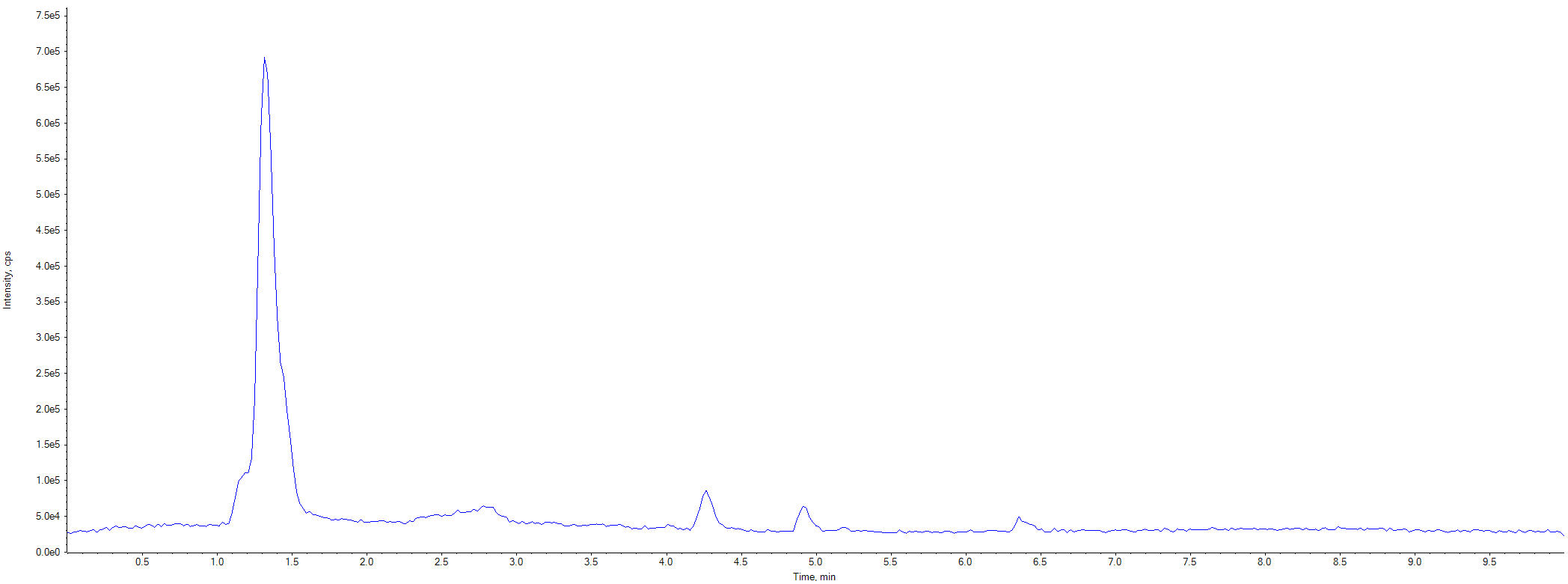


#### **Sample Name:** QC_100 **Vial #:** 31

####
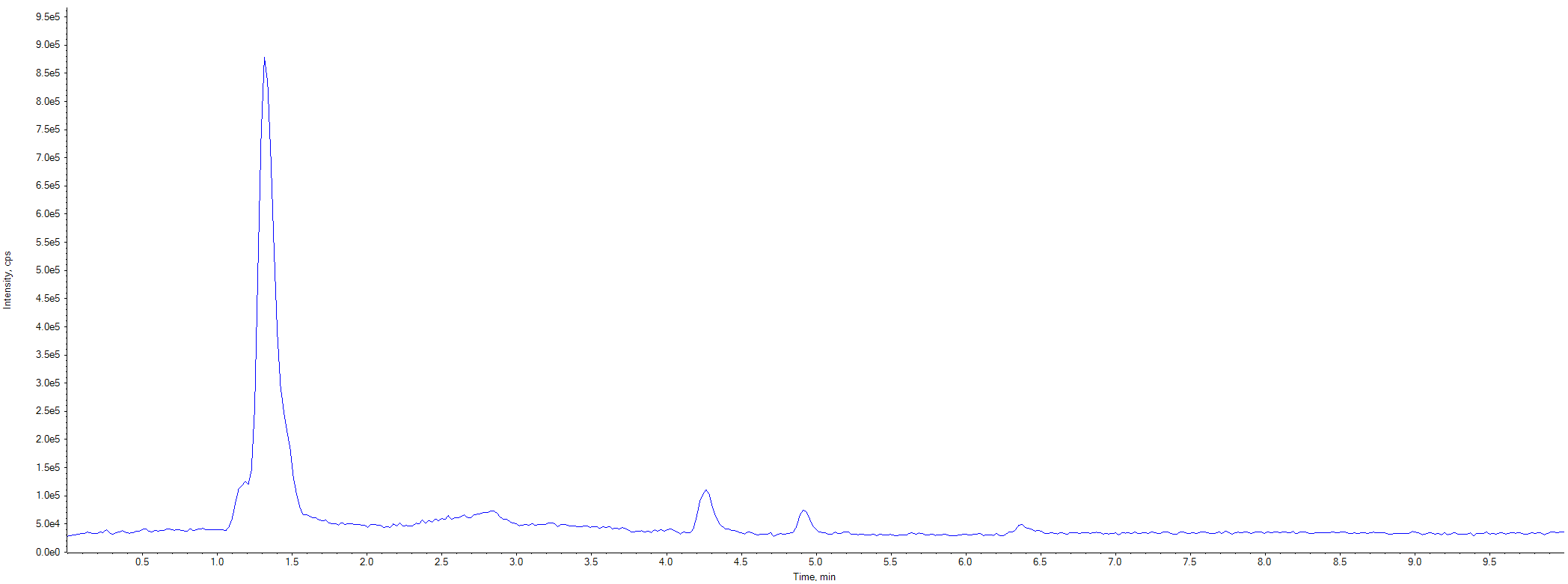

Supplement: Supplementary file 3 [file Table3.docx]
