# Supplementary material for: Elucidation of the antipyretic and anti-inflammatory effect of 8-O-Acetyl Shanzhiside methyl ester based on intestinal flora and metabolomics analysis
Source: Front Pharmacol. 2025 Apr 28;16:1482323. doi: 10.3389/fphar.2025.1482323 (PMC12066650; doi:10.3389/fphar.2025.1482323)

#### **Sample Name:** A_1 **Vial #:** 22

####
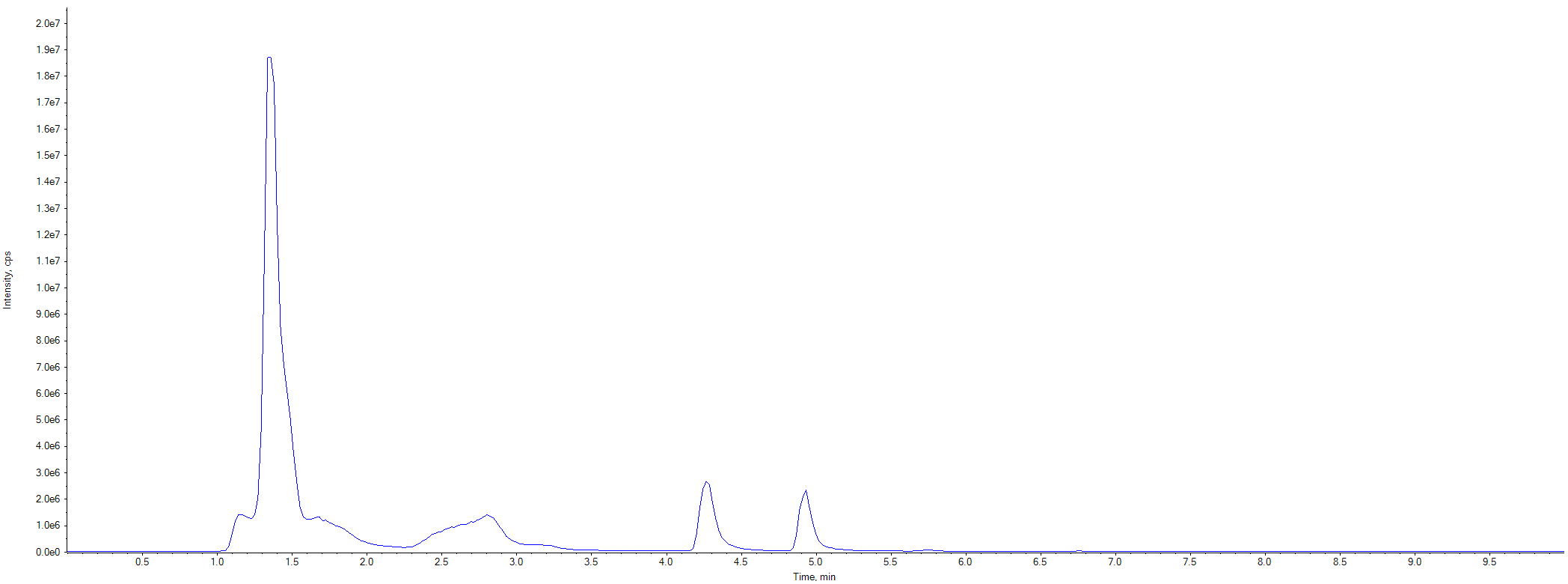


#### **Sample Name:** A_2 **Vial #:** 23

####
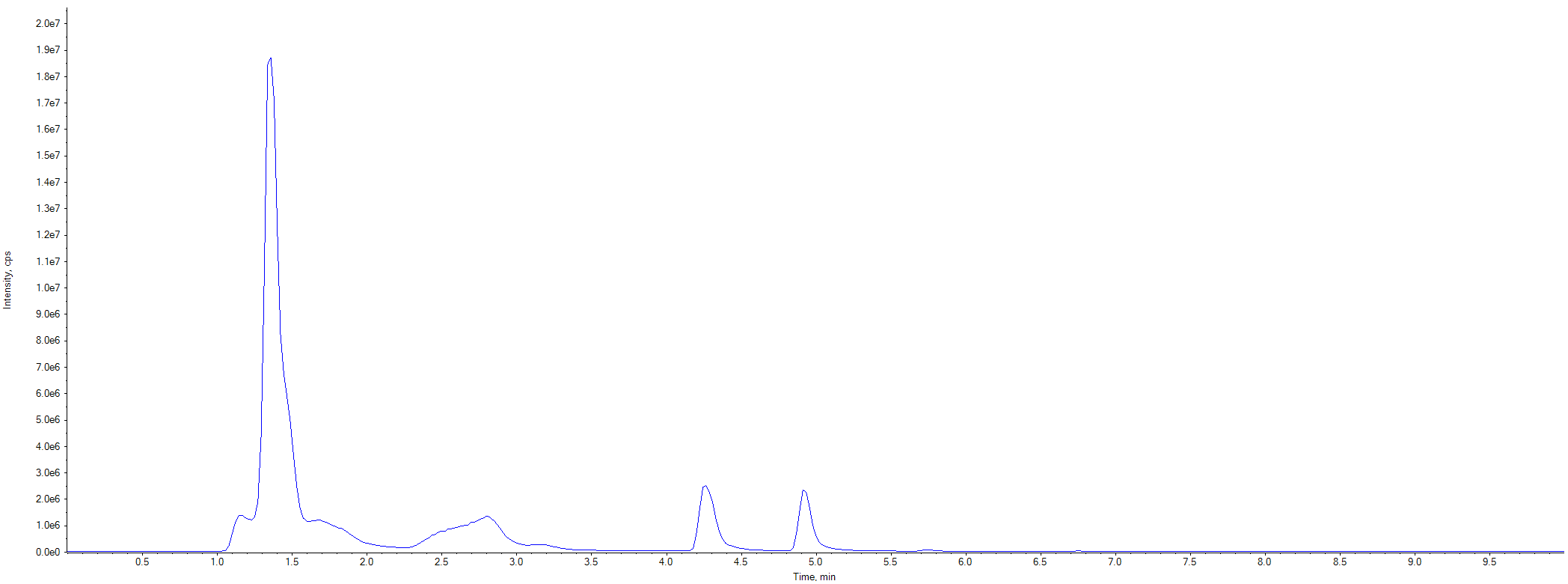


#### **Sample Name:** A_3 **Vial #:** 24

####
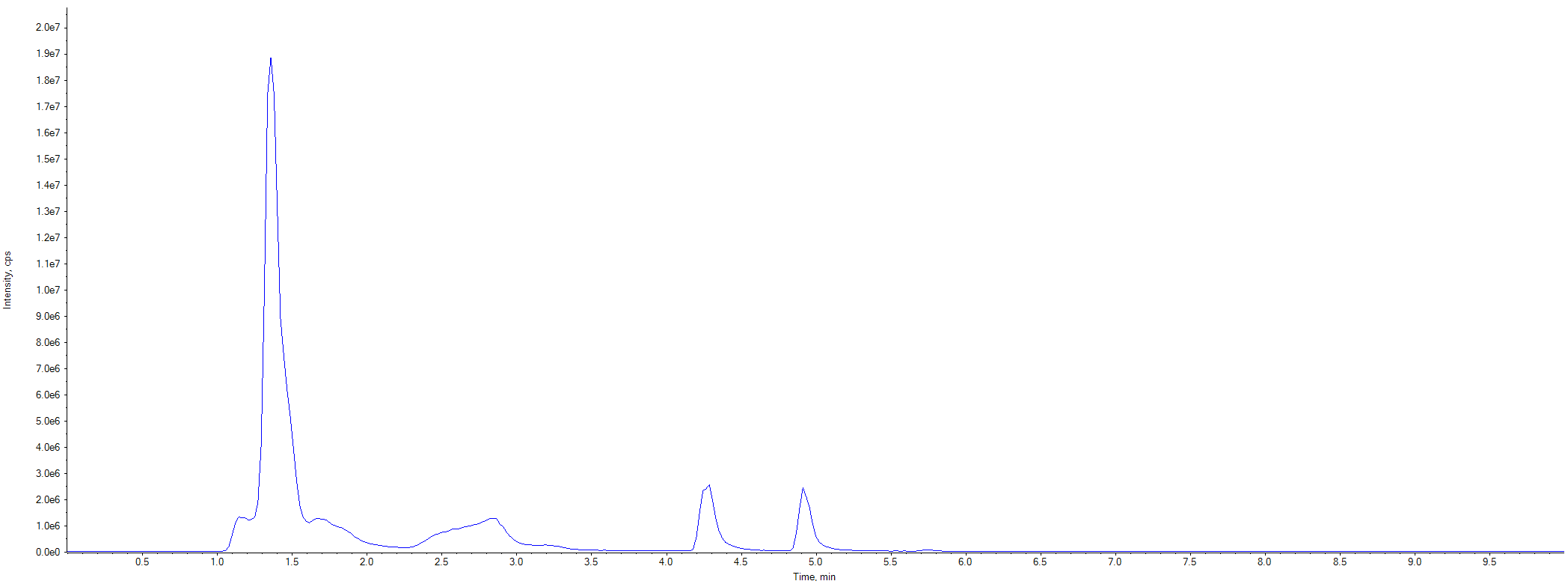


#### **Sample Name:** A_4 **Vial #:** 25

####
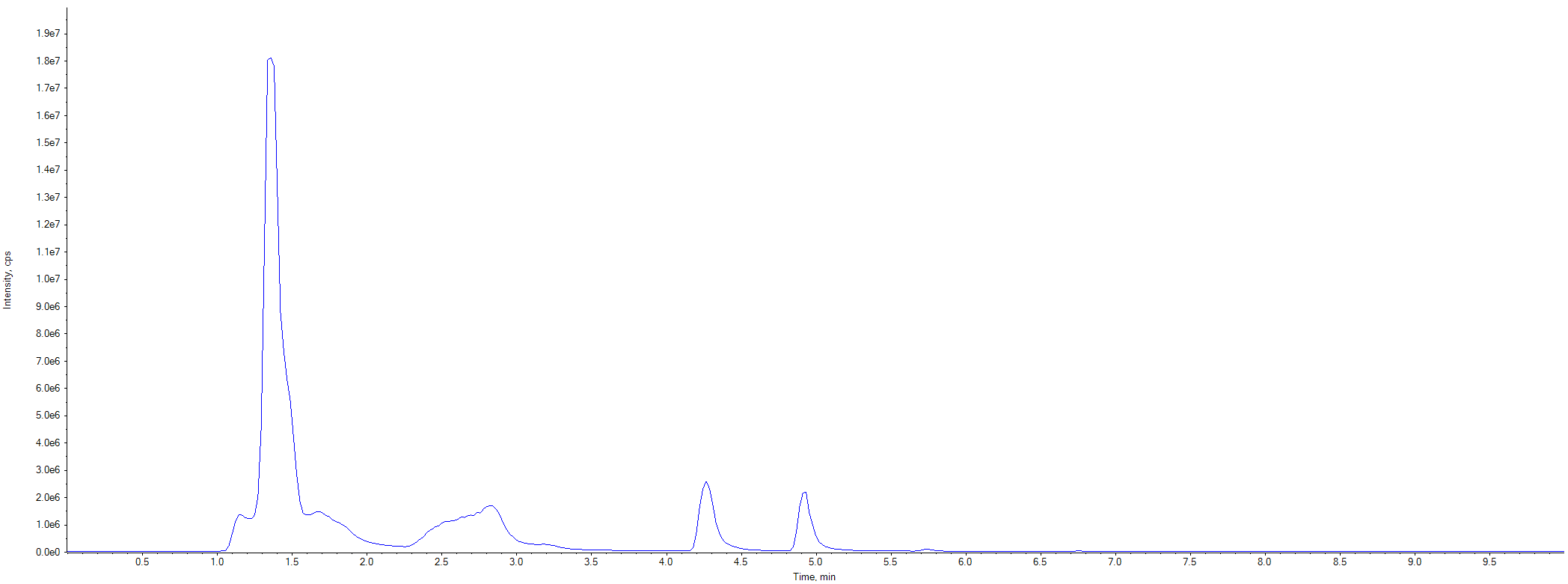


#### **Sample Name:** A_5 **Vial #:** 26

####
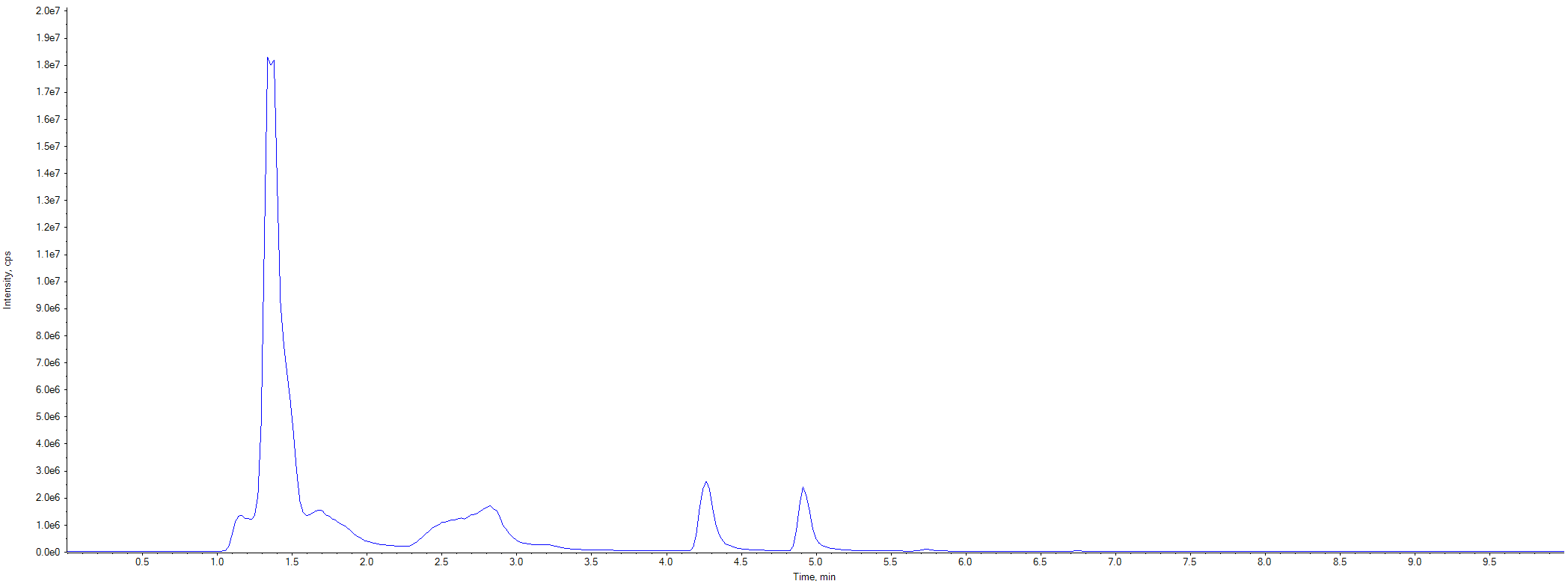


#### **Sample Name:** A_6 **Vial #:** 27

####
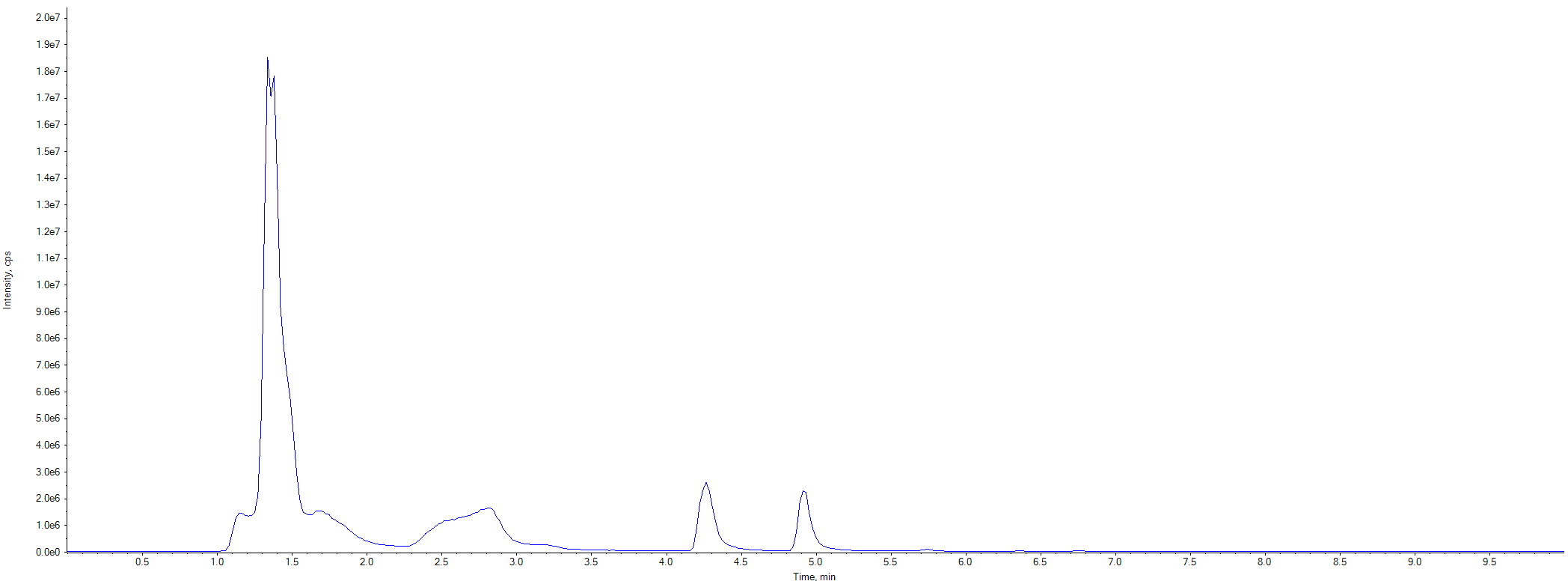


#### **Sample Name:** B_1 **Vial #:** 28

####
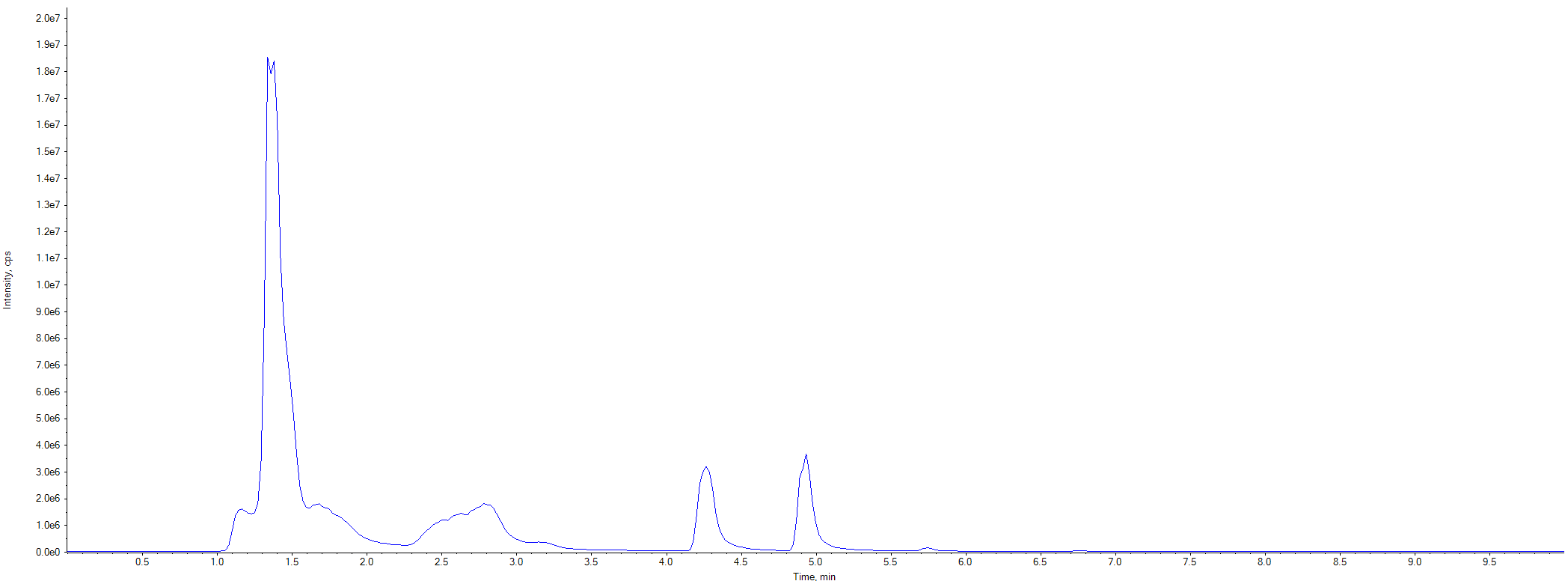


#### **Sample Name:** B_2 **Vial #:** 29

####
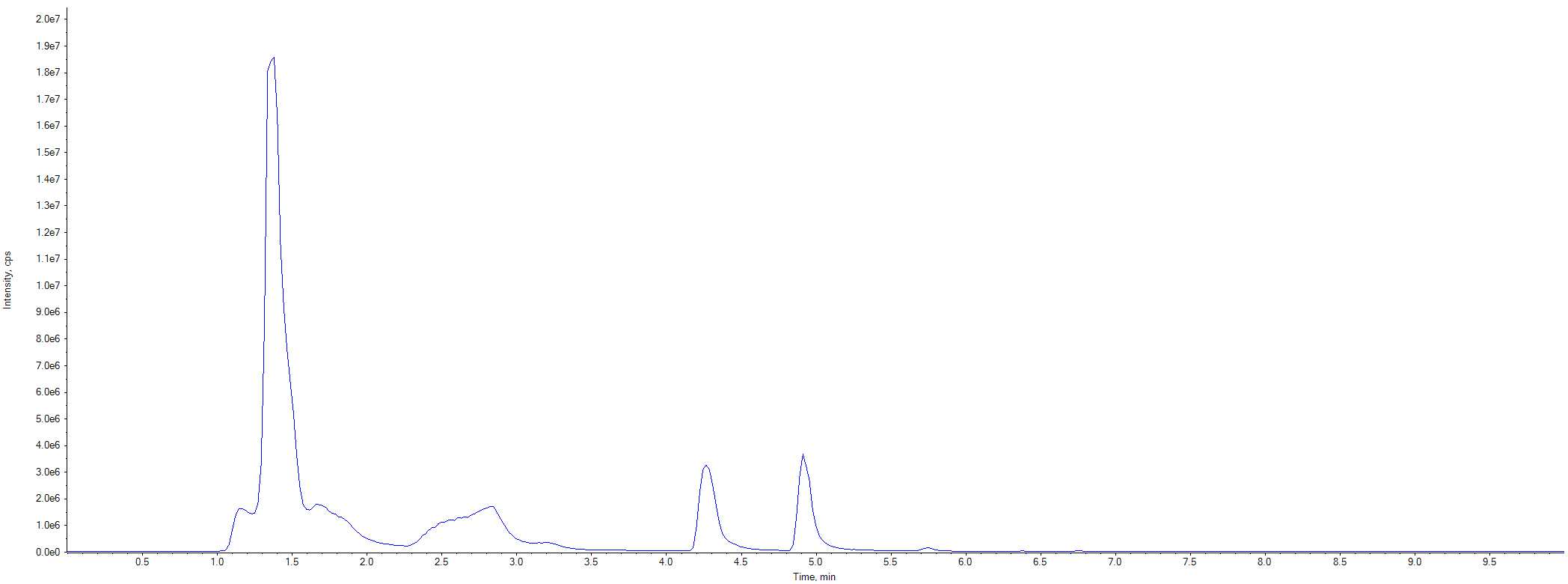


#### **Sample Name:** B_3 **Vial #:** 30

####
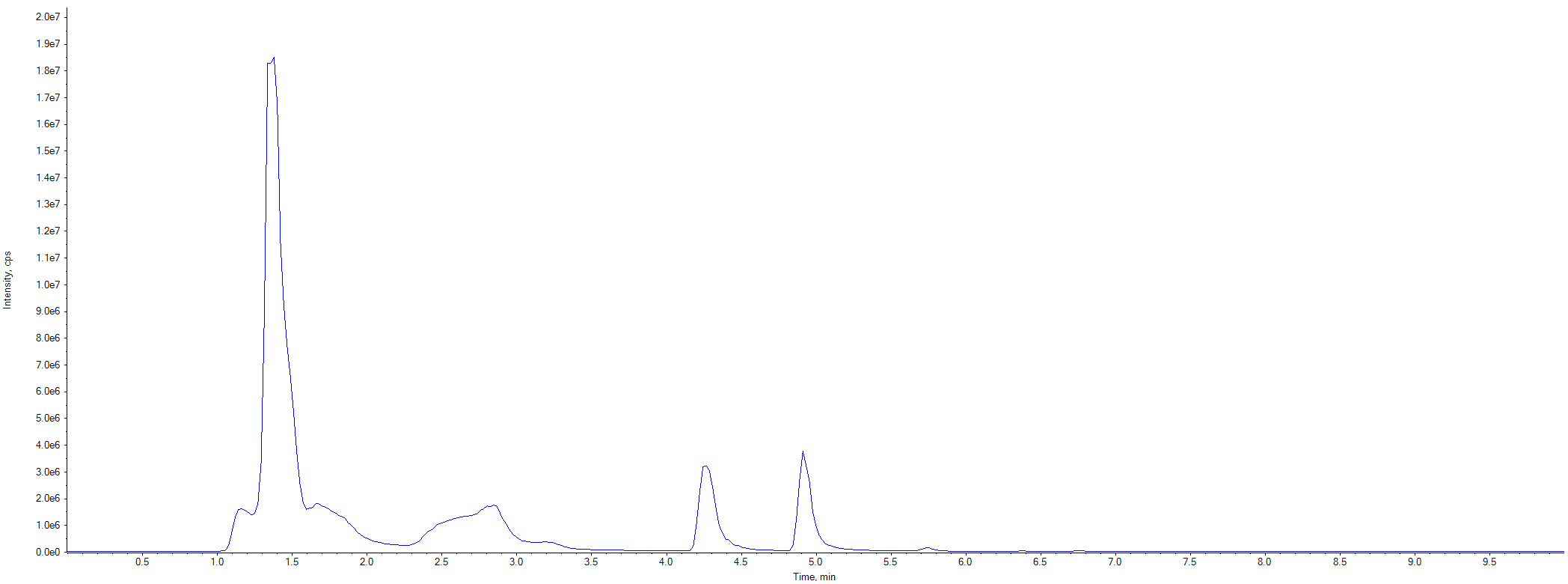


#### **Sample Name:** B_4 **Vial #:** 31

####
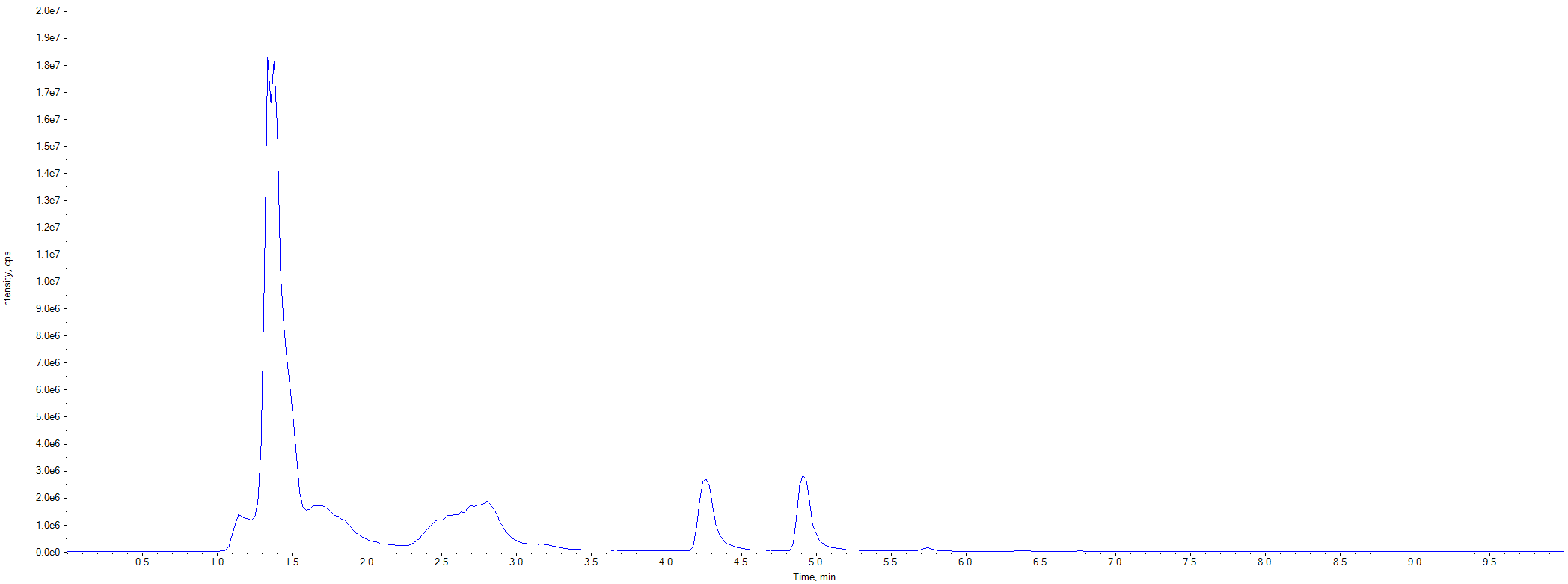


#### **Sample Name:** B_5 **Vial #:** 32

####
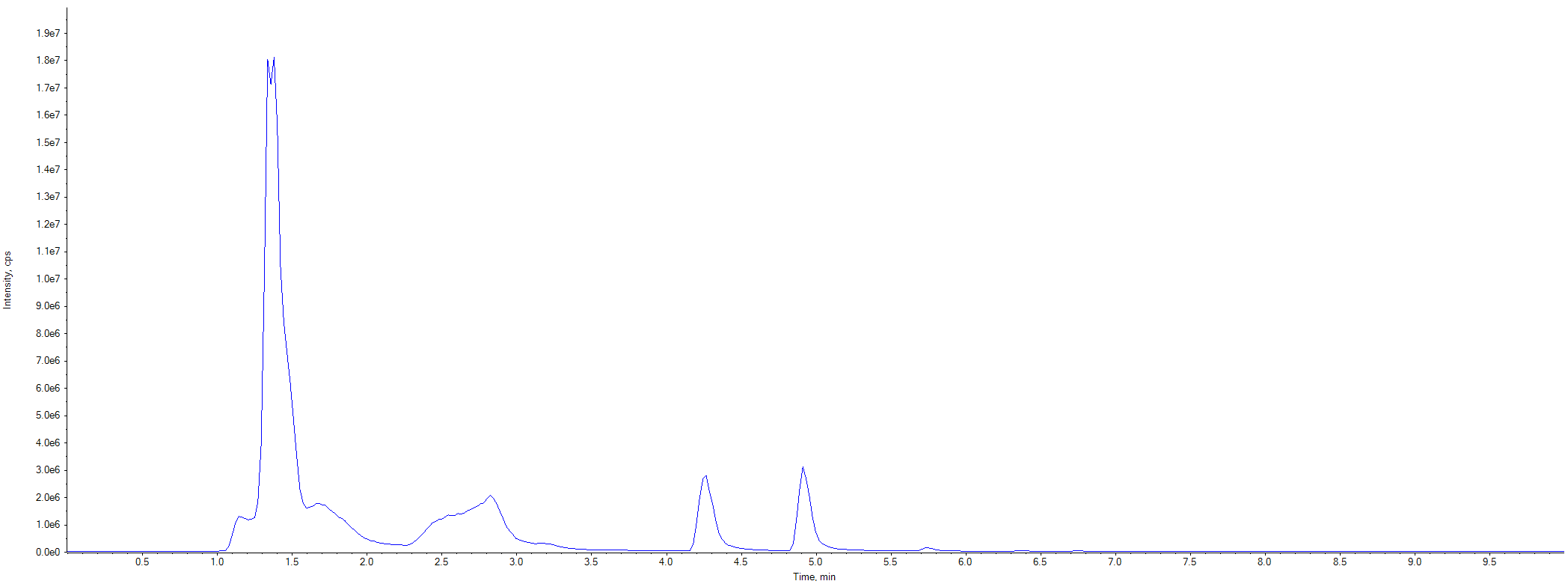


#### **Sample Name:** B_6 **Vial #:** 33

####
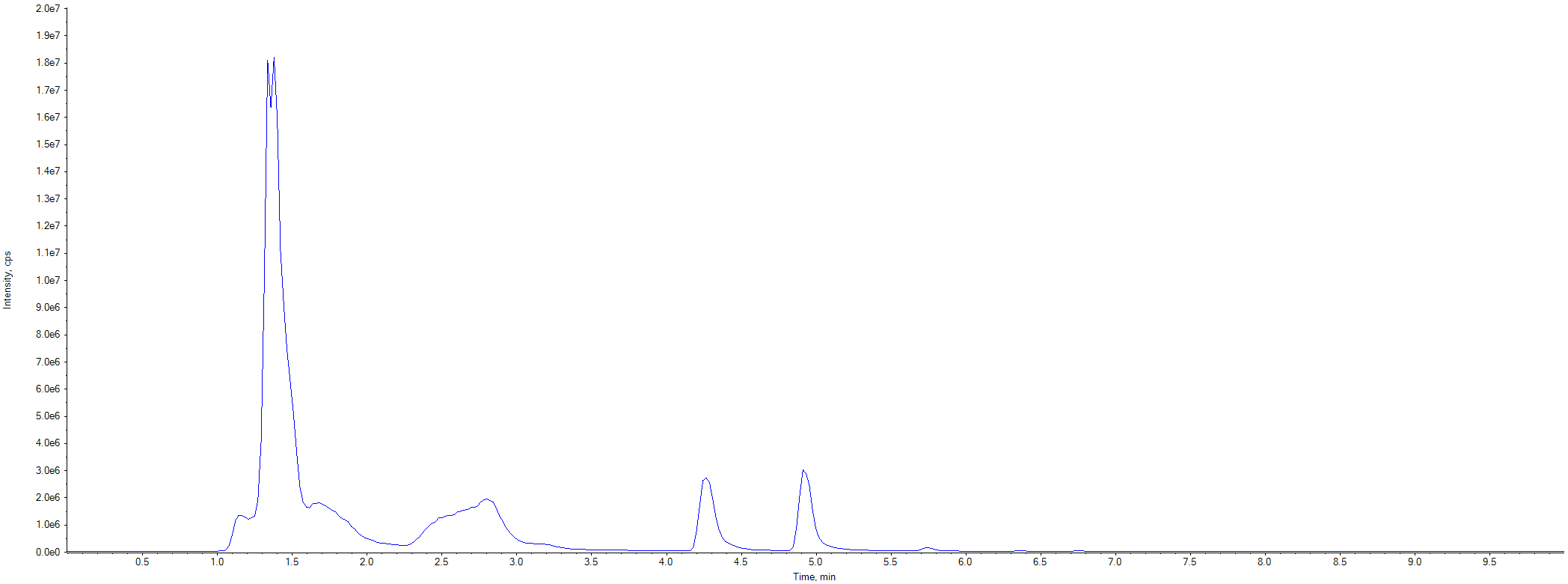


#### **Sample Name:** C_1 **Vial #:** 34

####
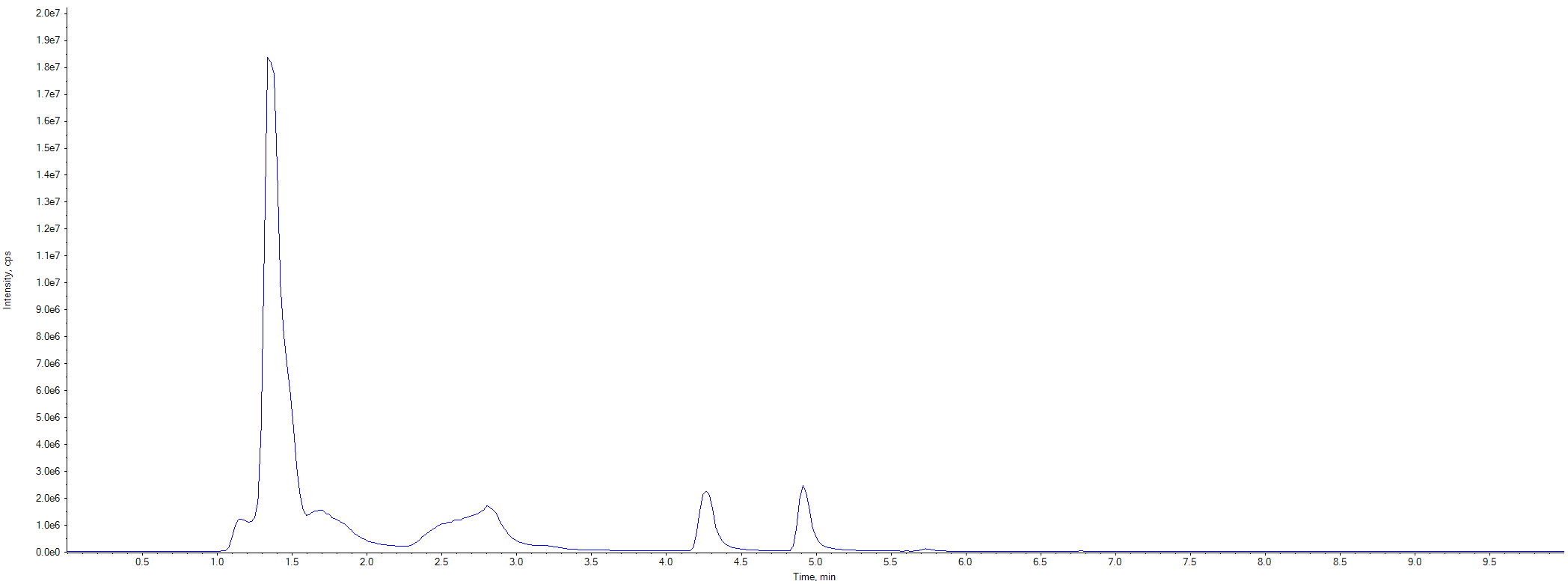


#### **Sample Name:** C_2 **Vial #:** 35

####
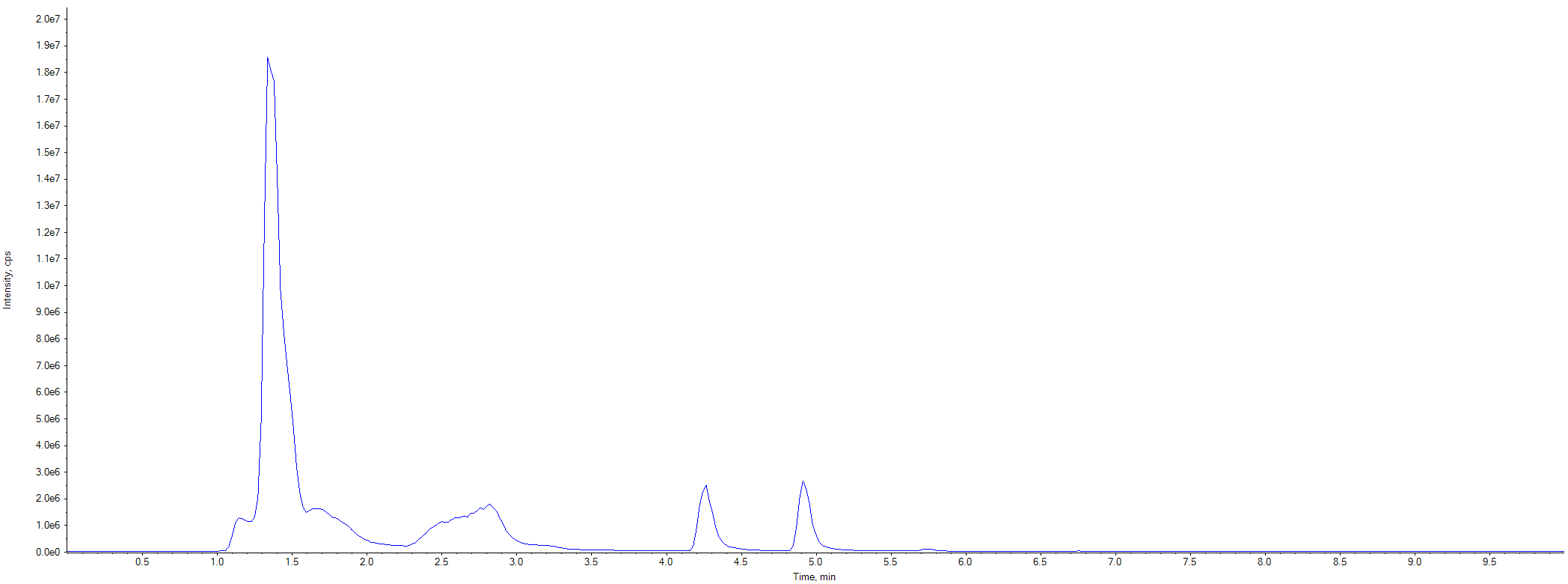


#### **Sample Name:** C_3 **Vial #:** 36

####
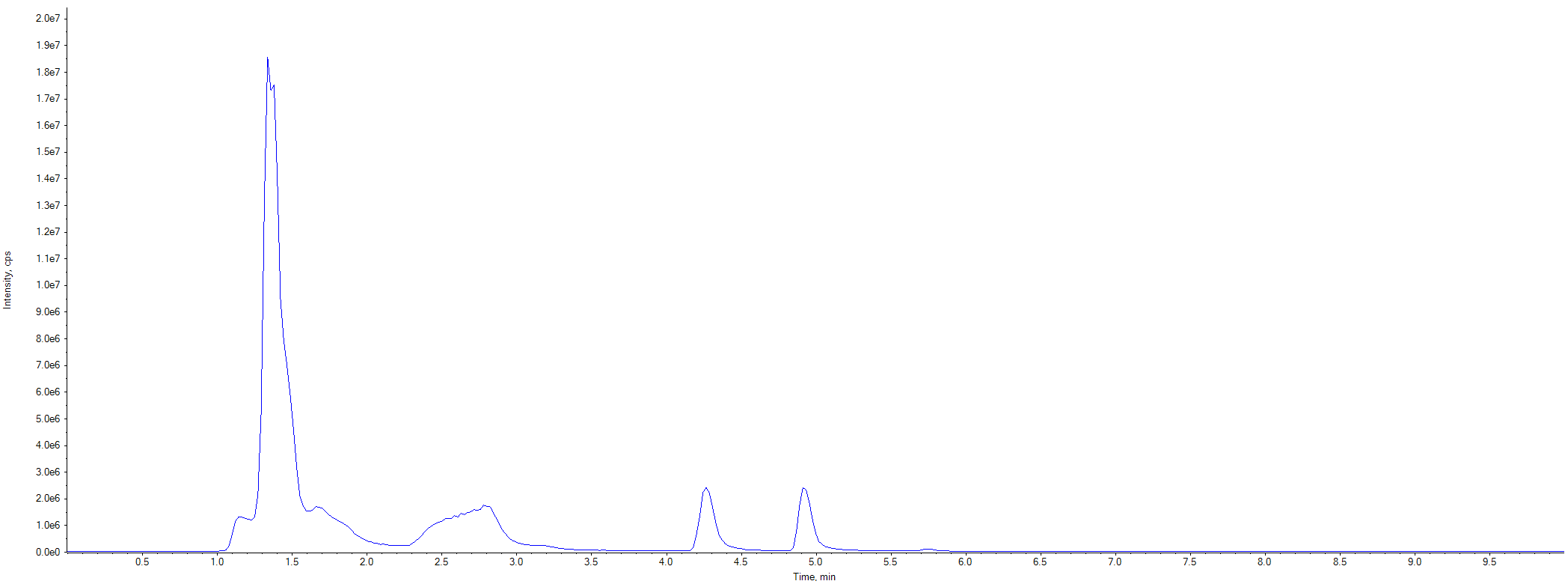


#### **Sample Name:** C_4 **Vial #:** 37

####
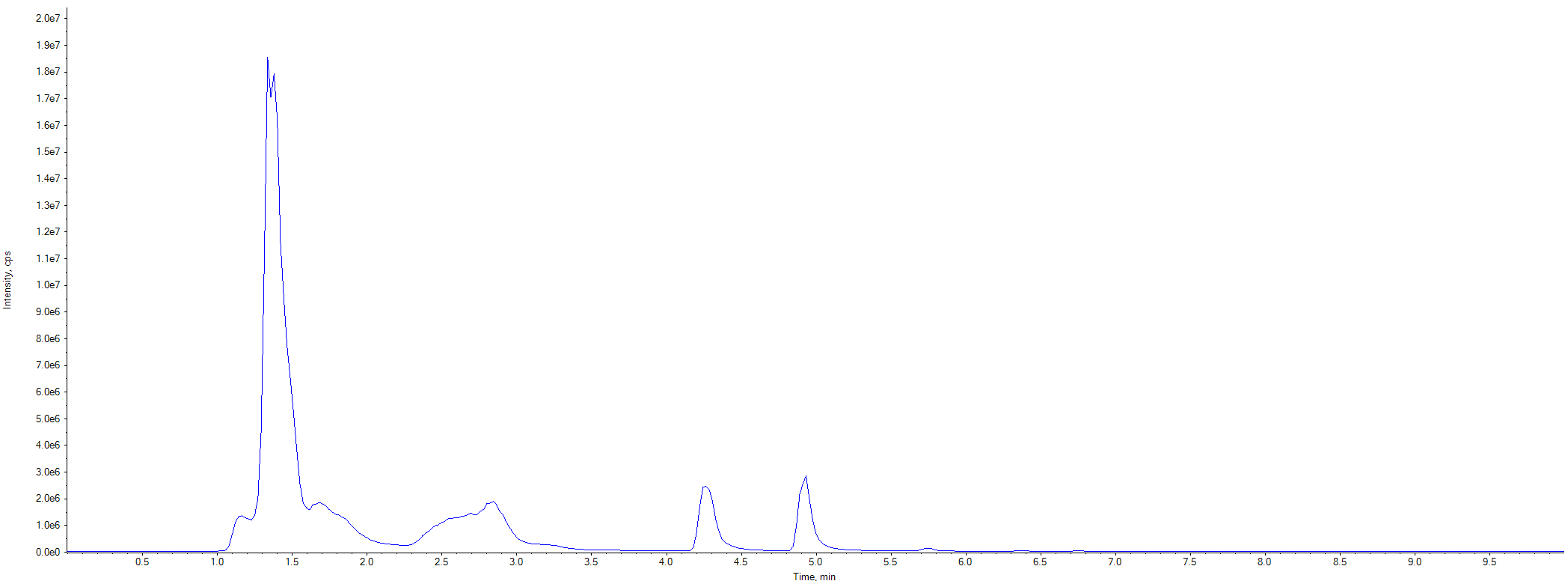


#### **Sample Name:** C_5 **Vial #:** 38

####
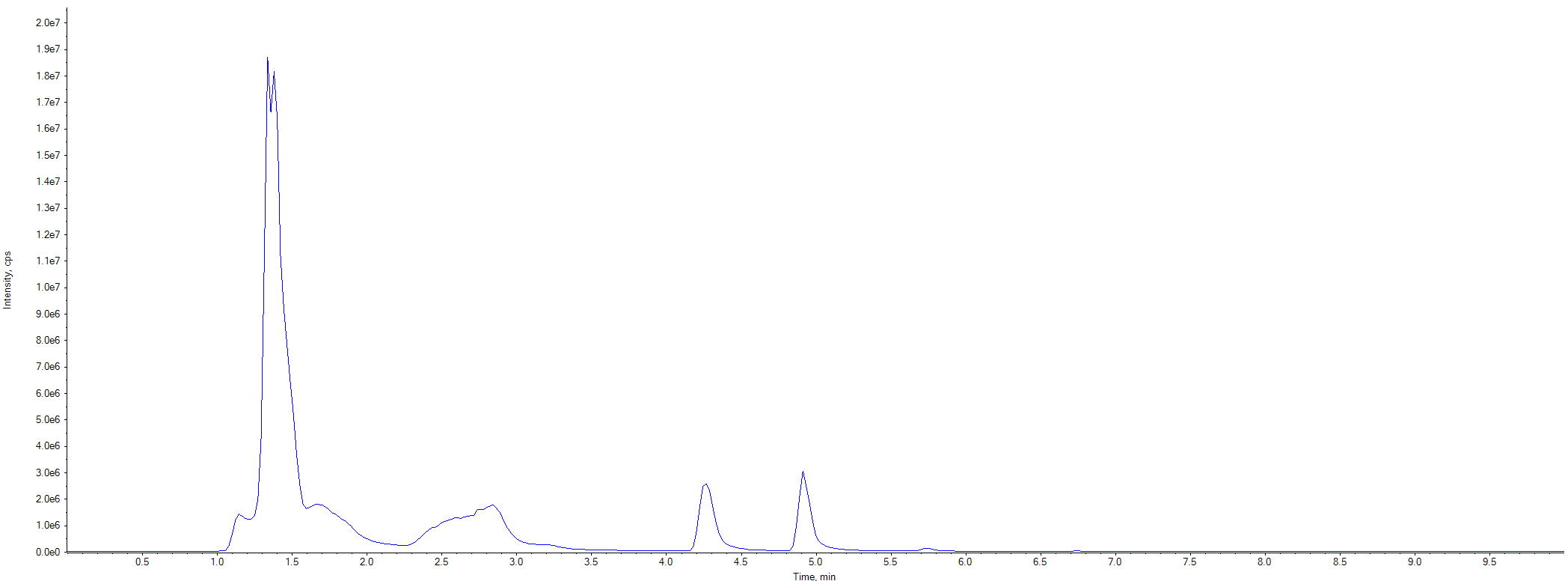


#### **Sample Name:** C_6 **Vial #:** 39

####
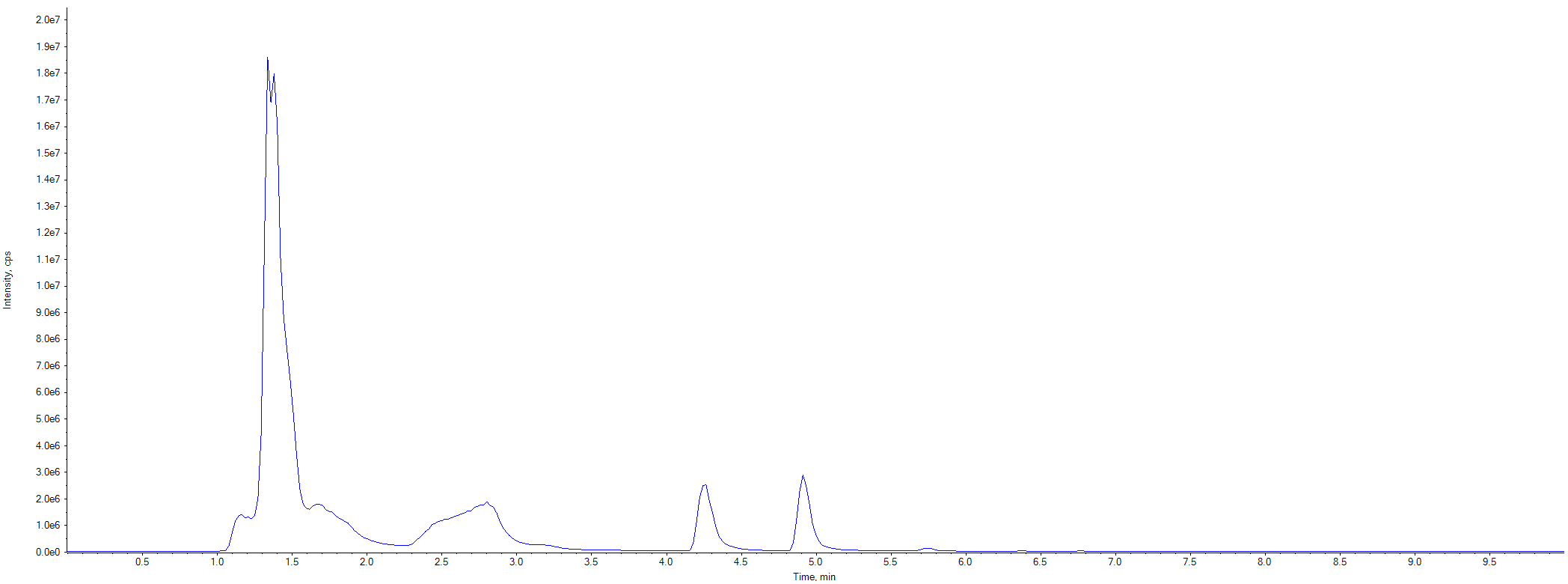


#### **Sample Name:** QC **Vial #:** 21

####
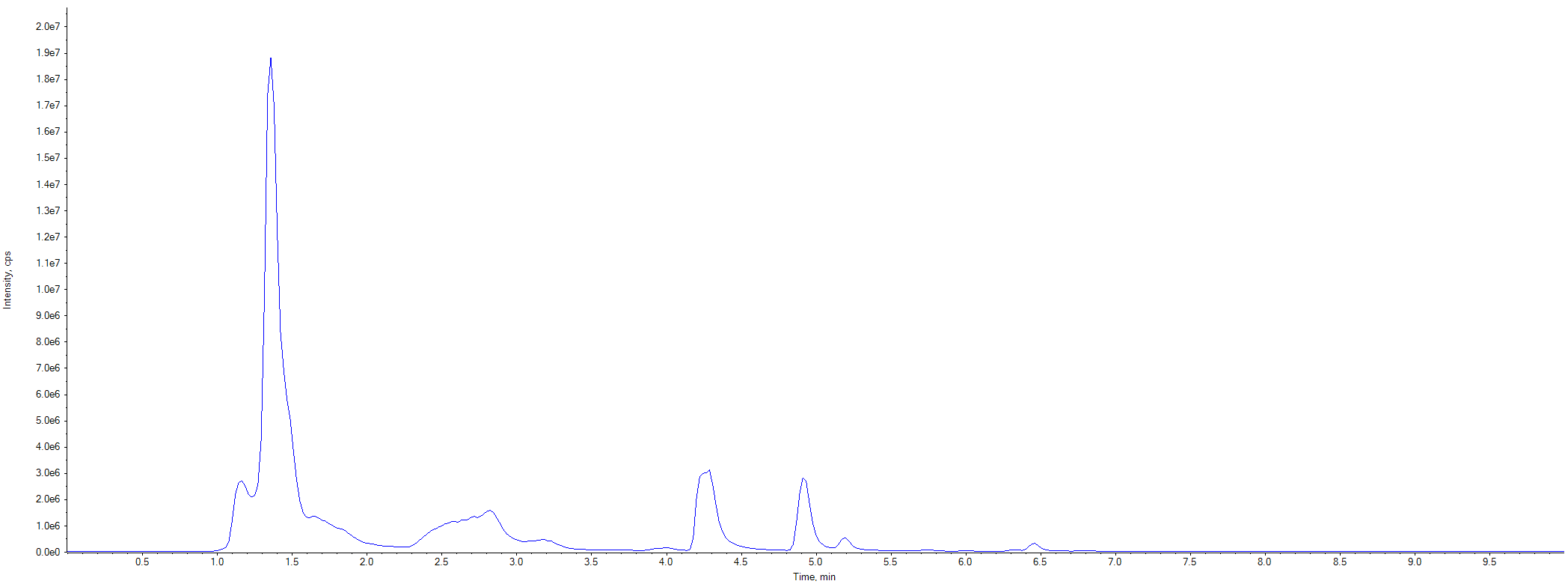


#### **Sample Name:** QC **Vial #:** 21

####
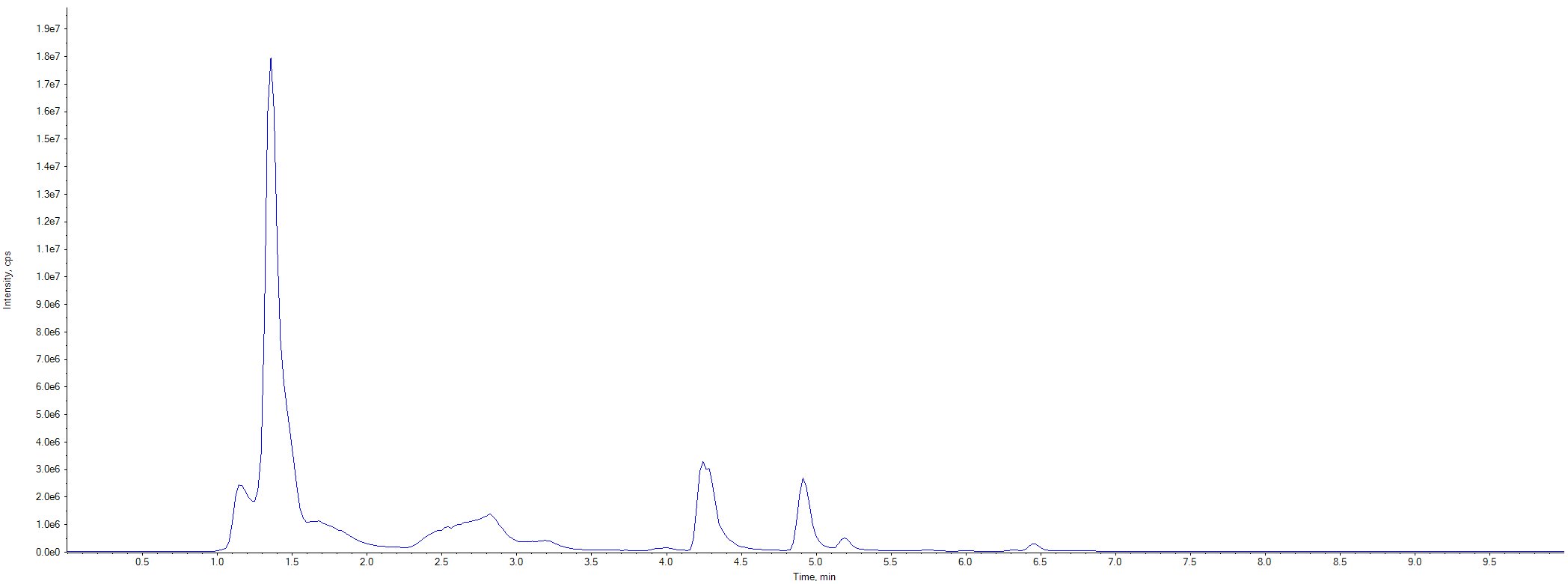


#### **Sample Name:** QC **Vial #:** 21

####
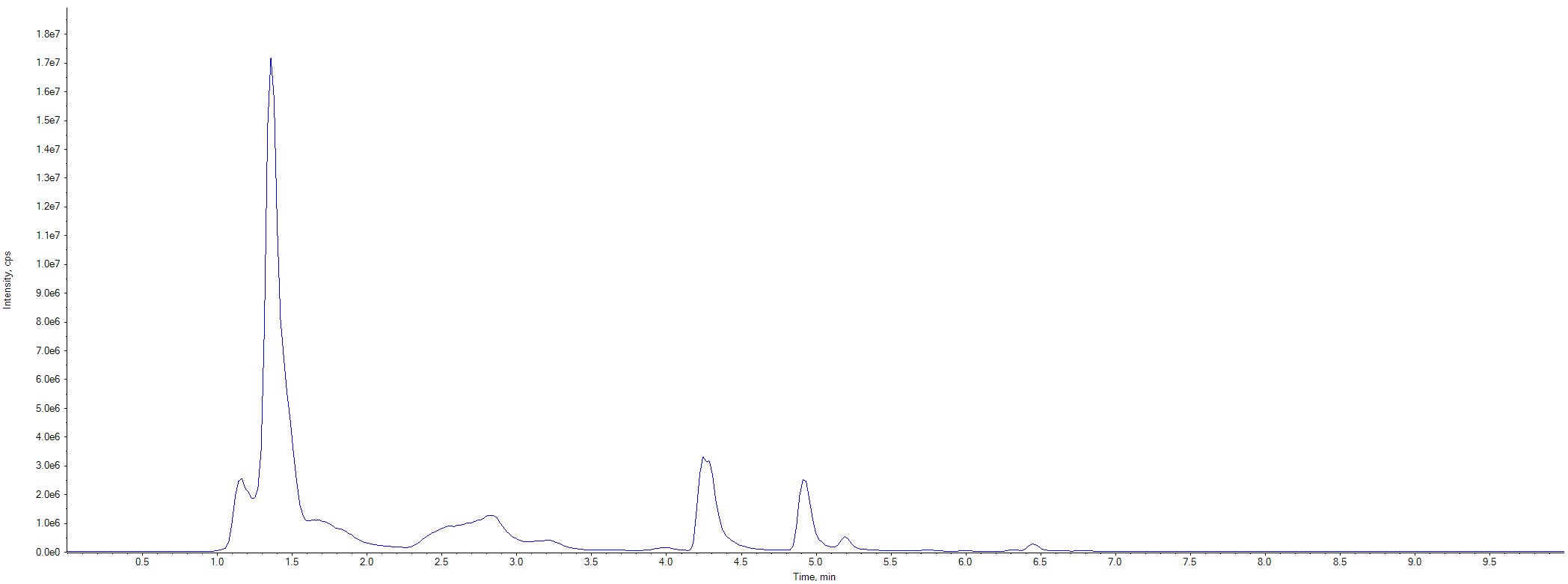


#### **Sample Name:** QC **Vial #:** 21

####
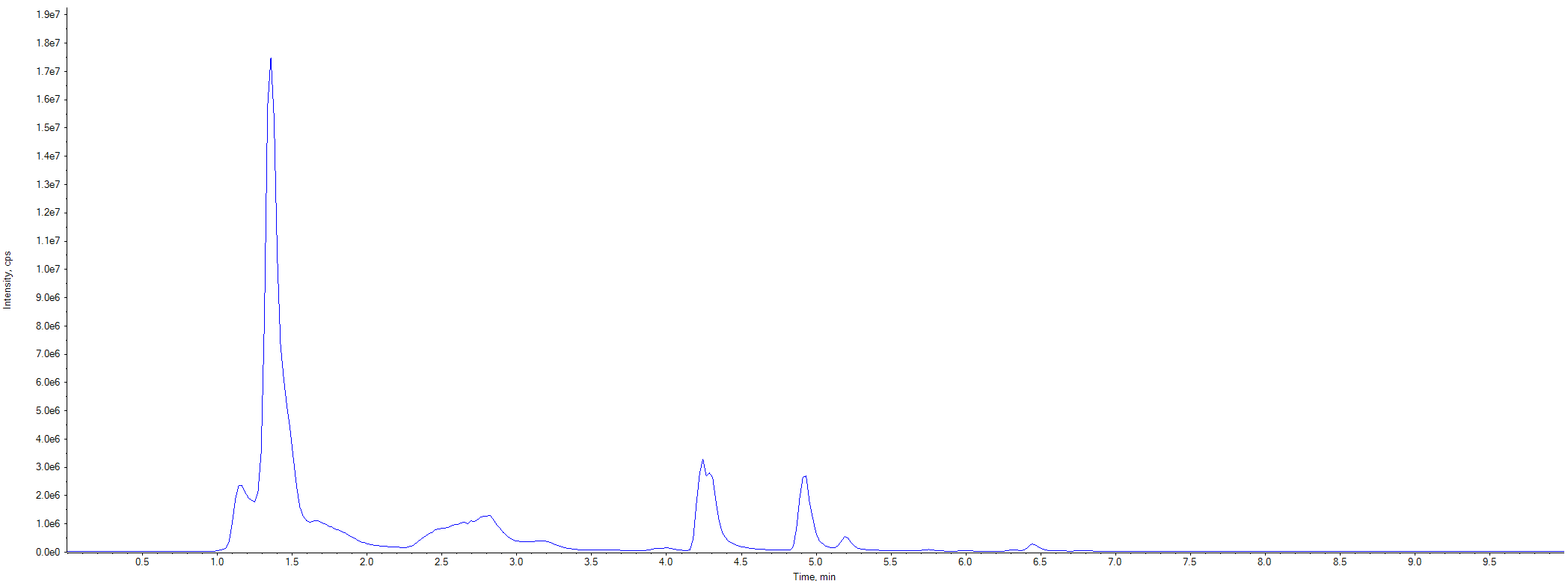


#### **Sample Name:** A_1_100 **Vial #:** 32

####
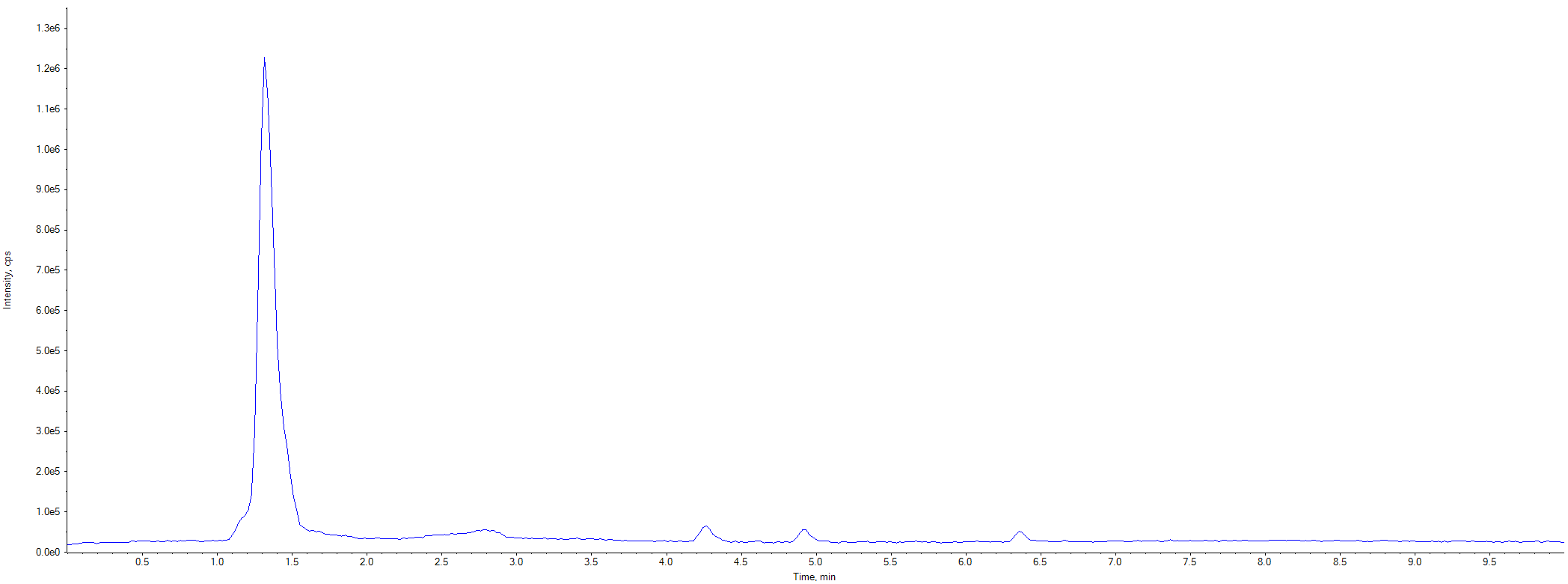


#### **Sample Name:** A_2_100 **Vial #:** 33

####
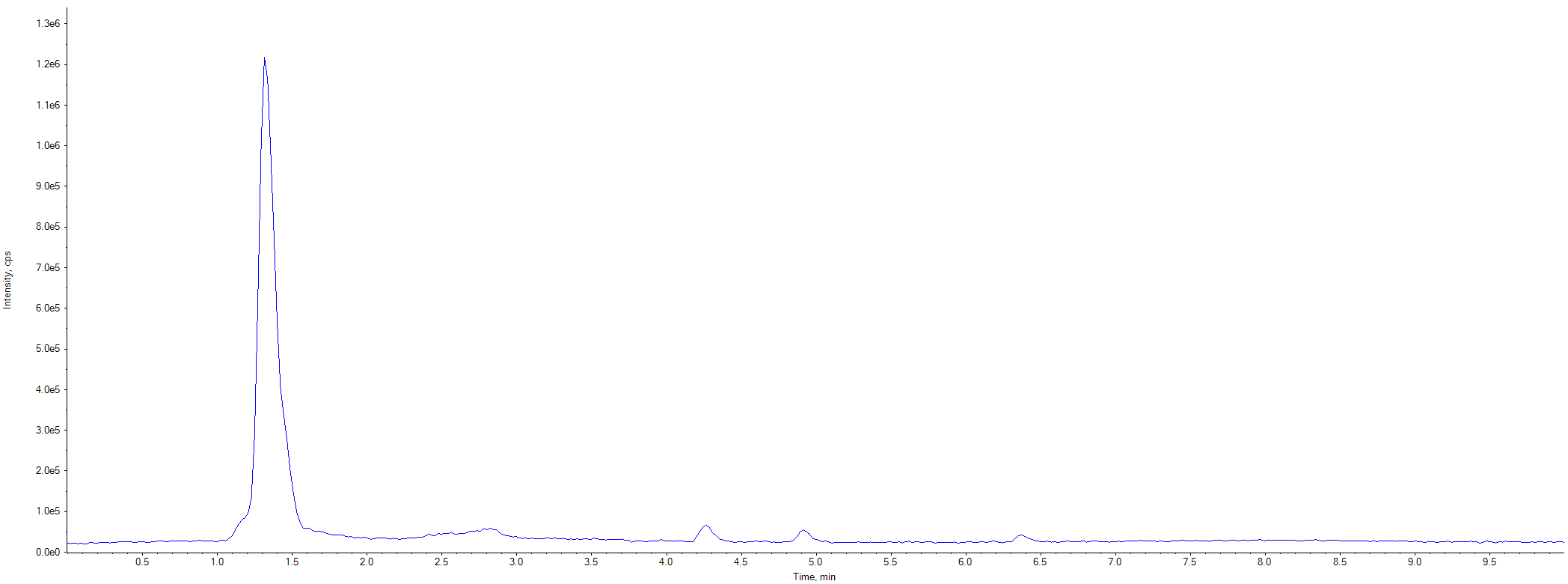


#### **Sample Name:** A_3_100 **Vial #:** 34

####
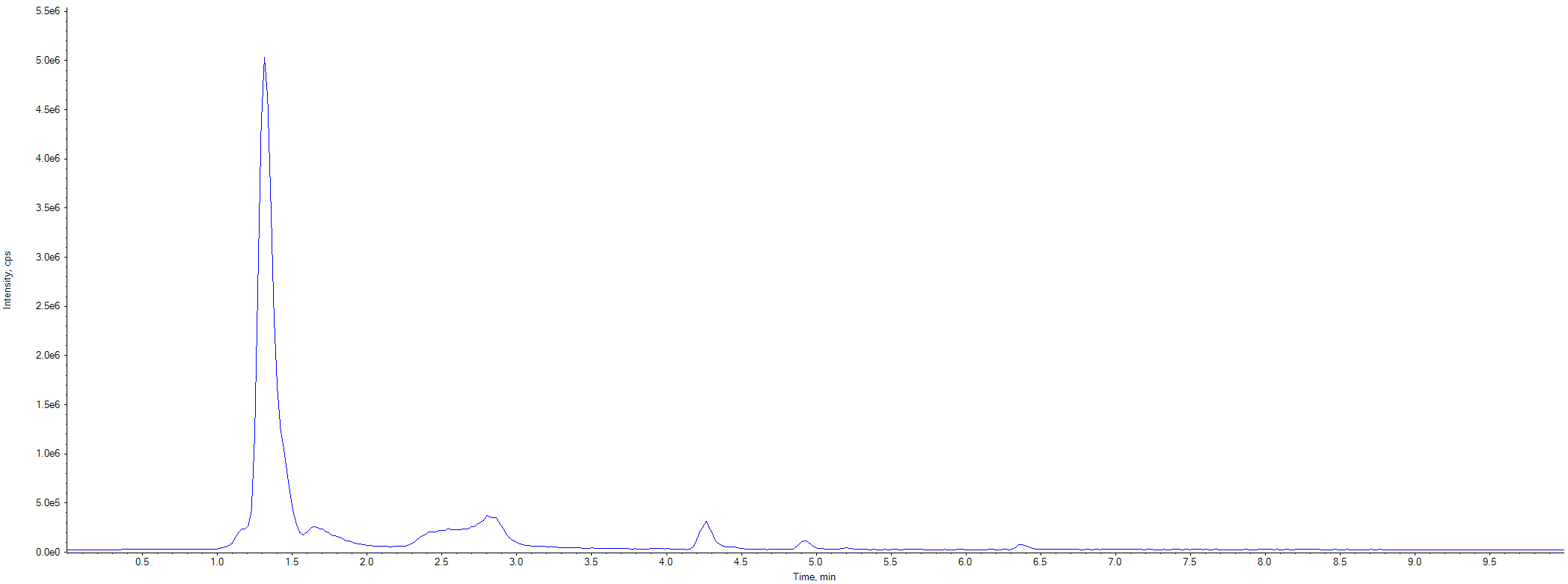


#### **Sample Name:** A_4_100 **Vial #:** 35

####
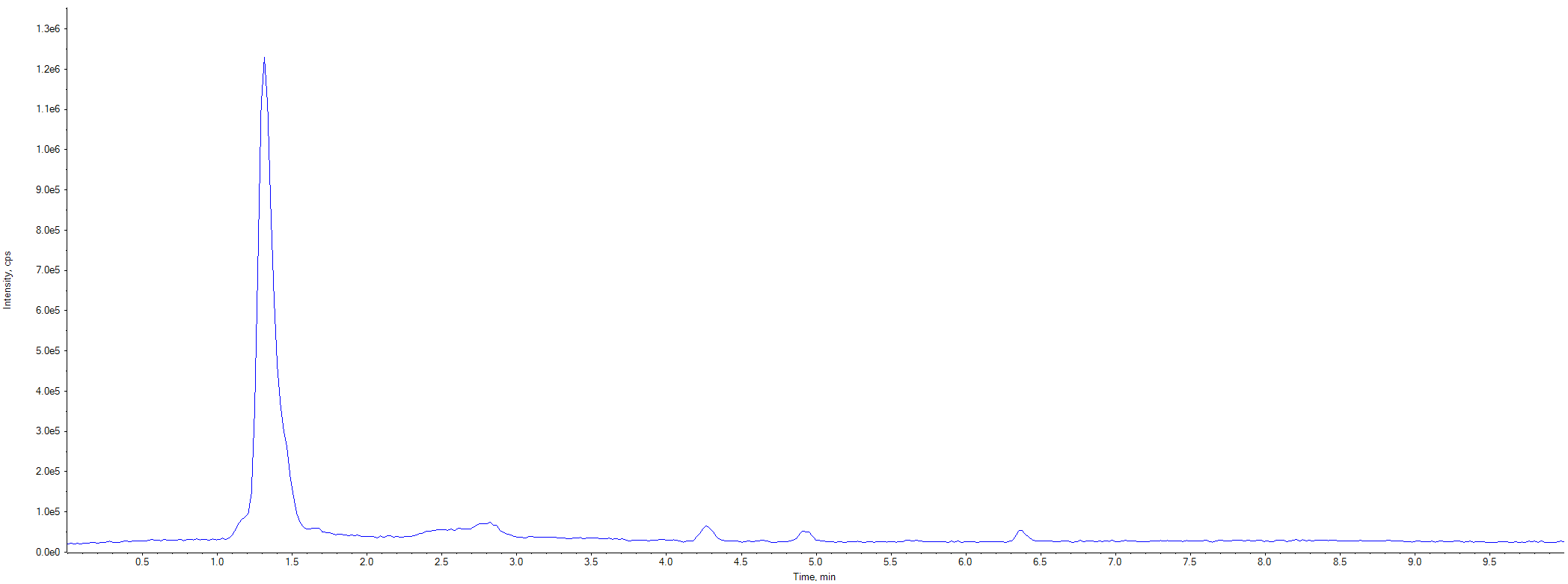


#### **Sample Name:** A_5_100 **Vial #:** 36

####
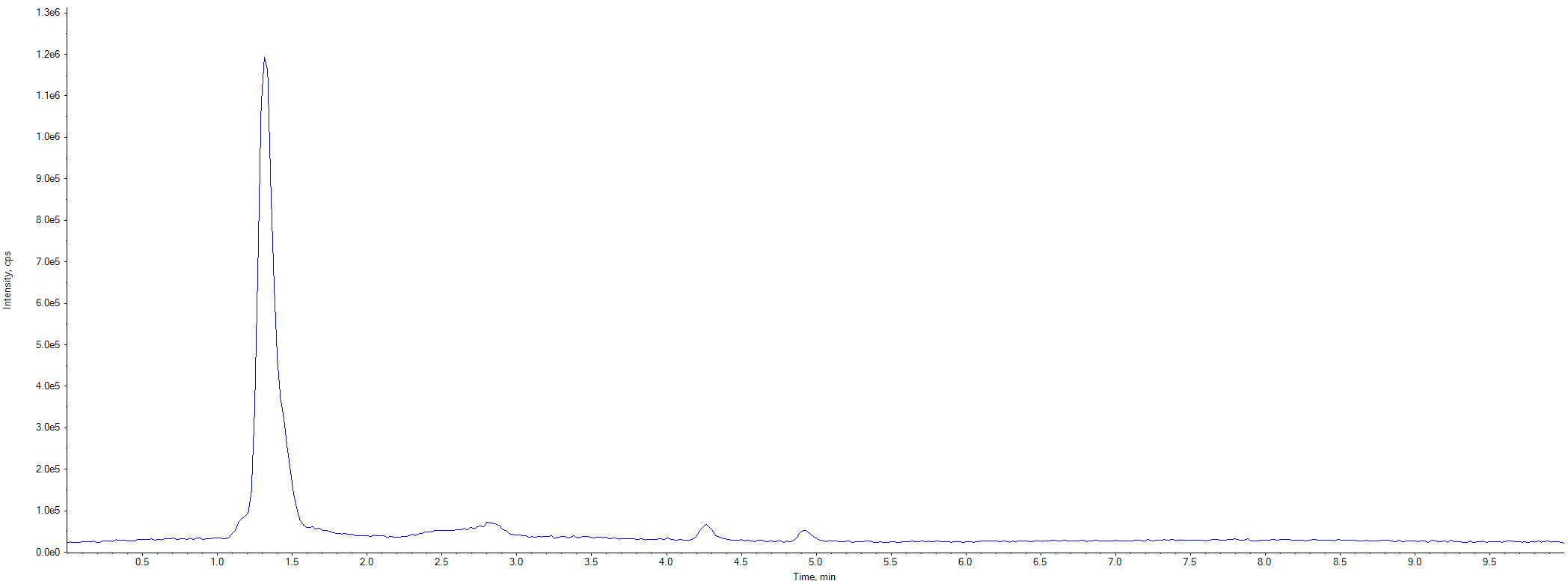


#### **Sample Name:** A_6_100 **Vial #:** 37

####
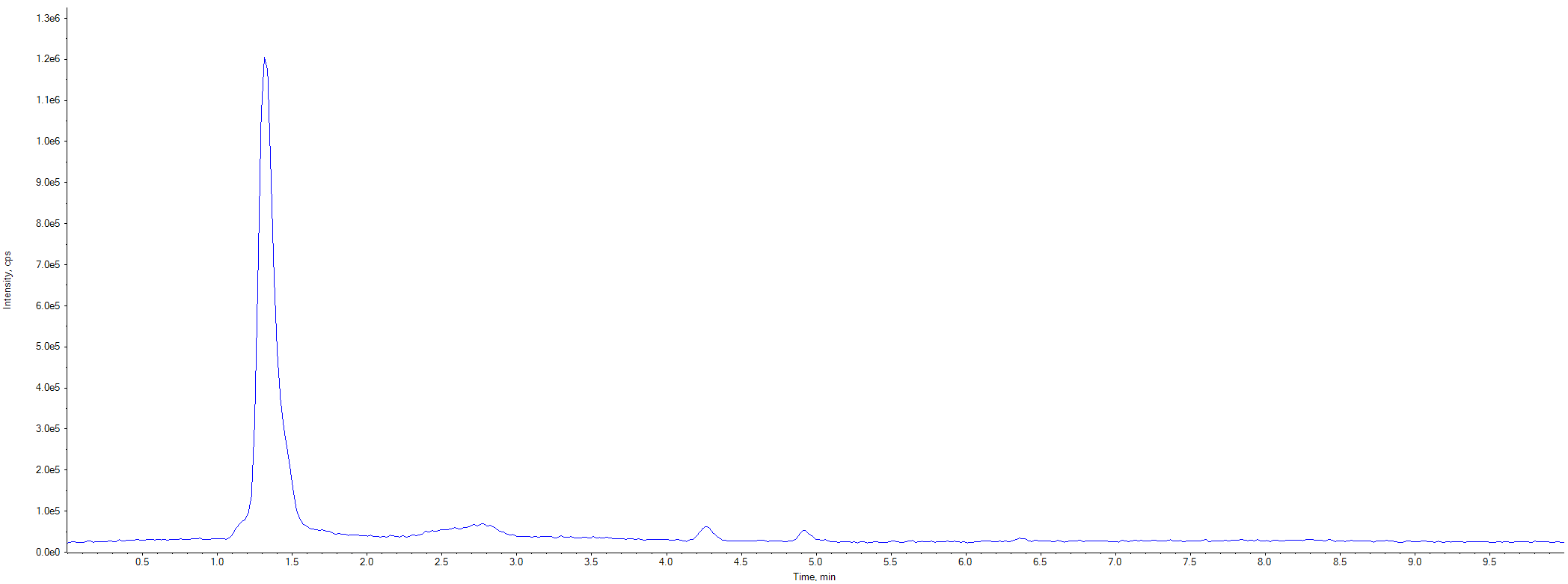


#### **Sample Name:** B_1_100 **Vial #:** 38

####
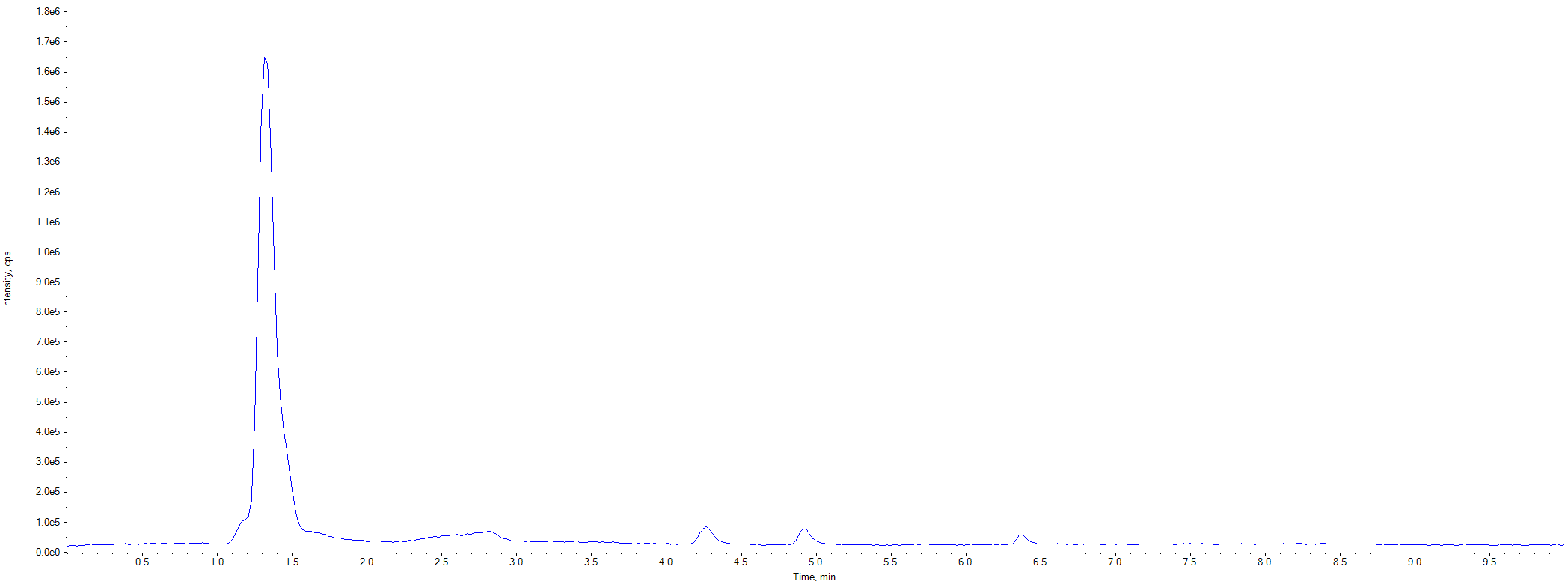


#### **Sample Name:** B_2_100 **Vial #:** 39

####
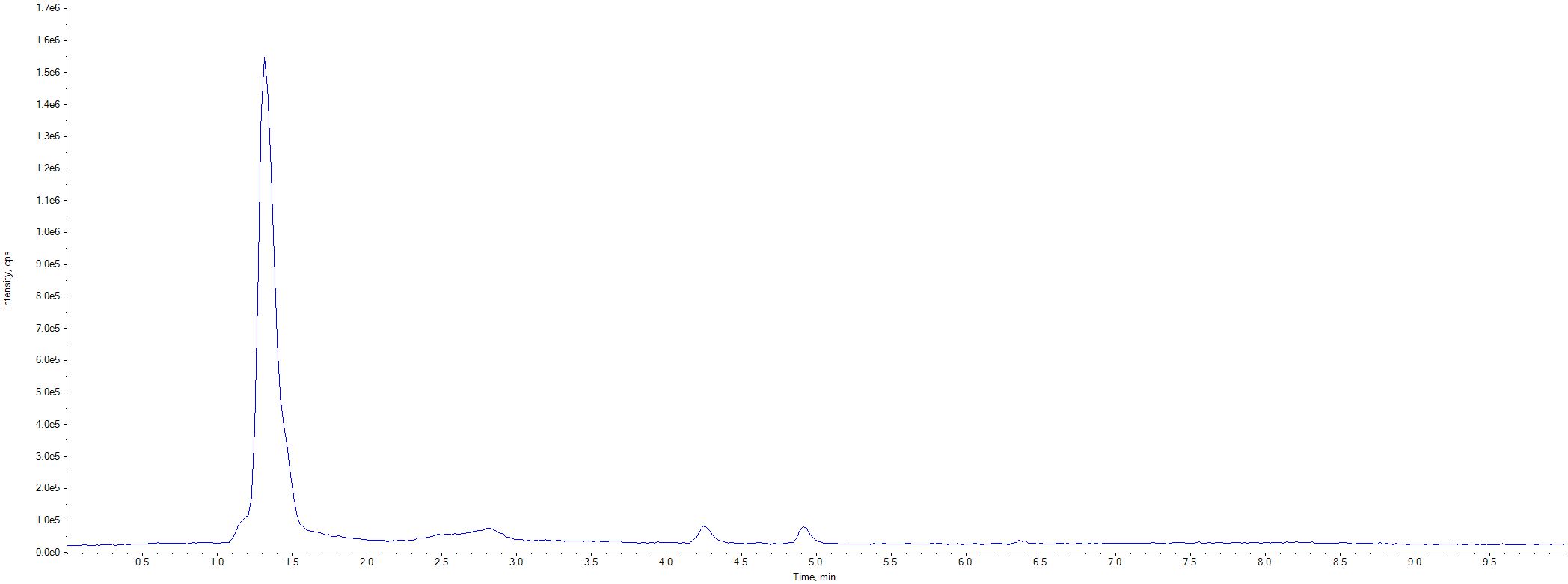


#### **Sample Name:** B_3_100 **Vial #:** 40

####
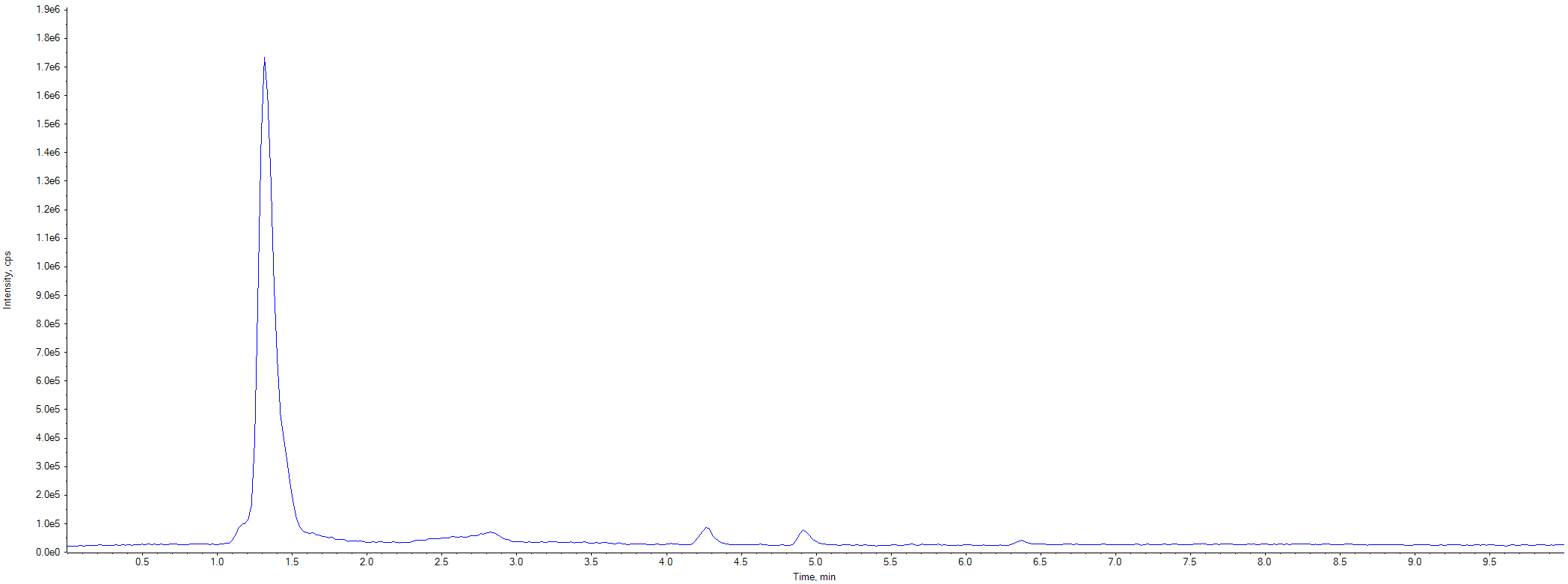


#### **Sample Name:** B_4_100 **Vial #:** 41

####
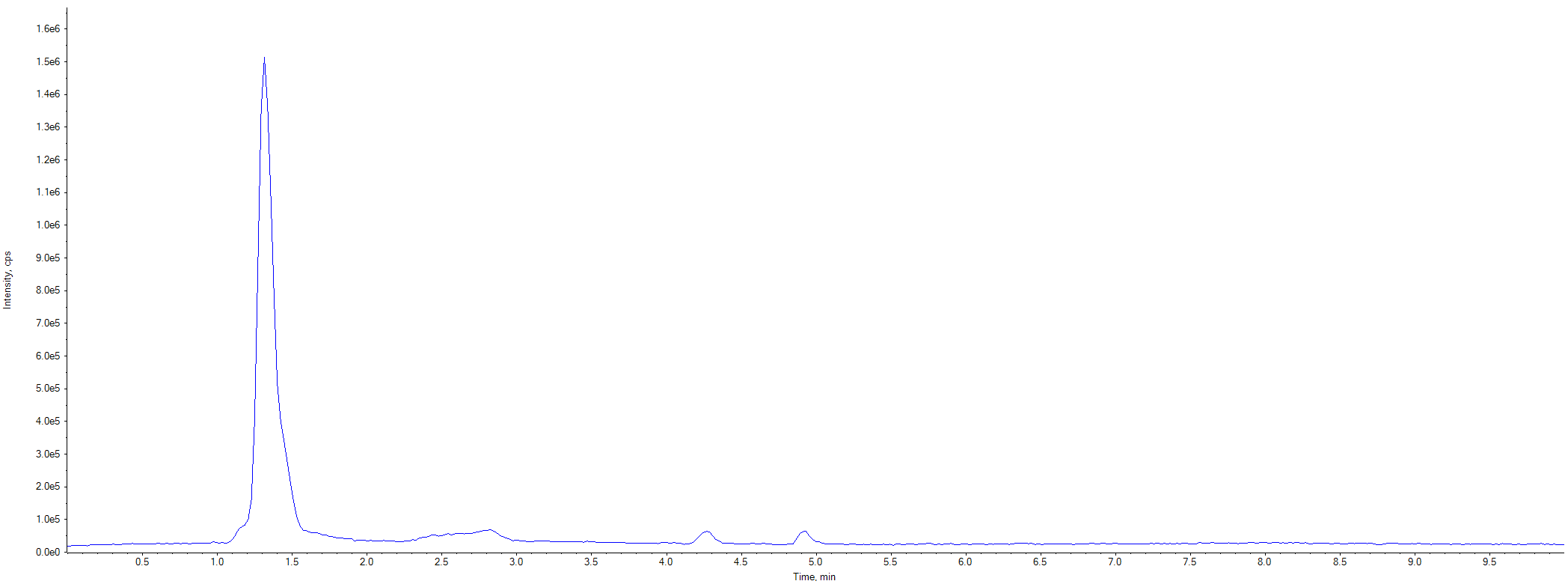


#### **Sample Name:** B_5_100 **Vial #:** 42

####
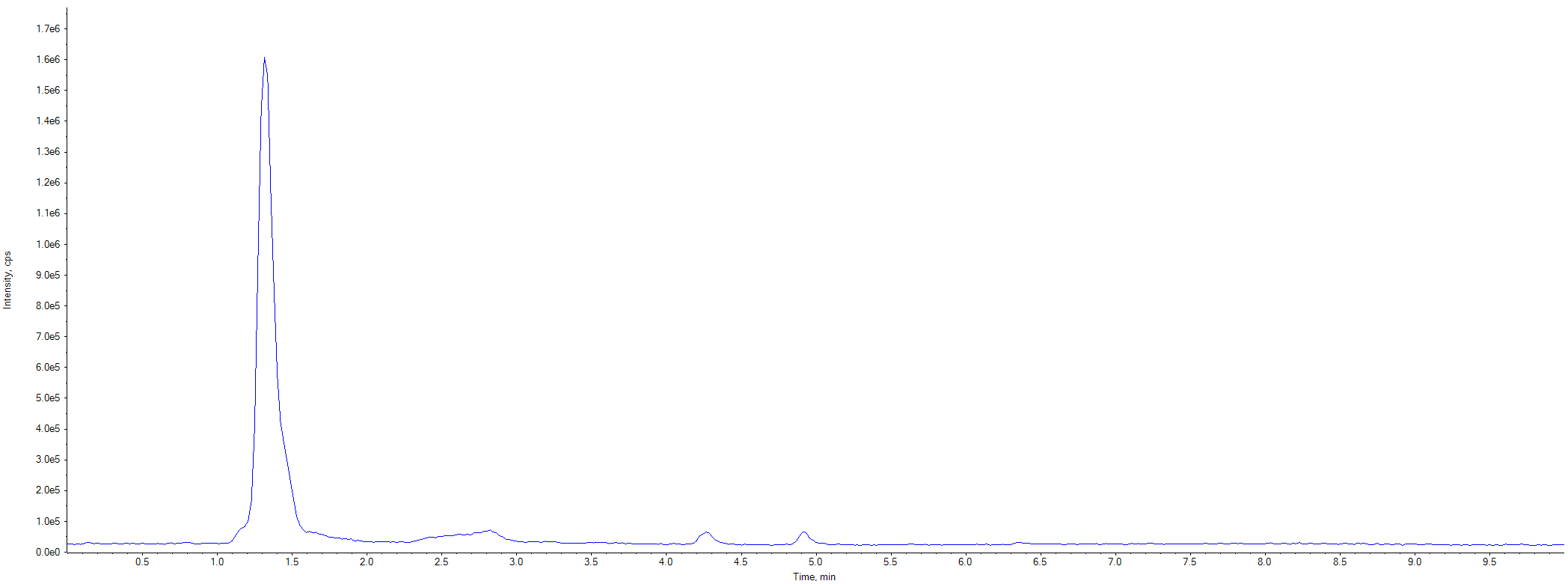


#### **Sample Name:** B_6_100 **Vial #:** 43

####
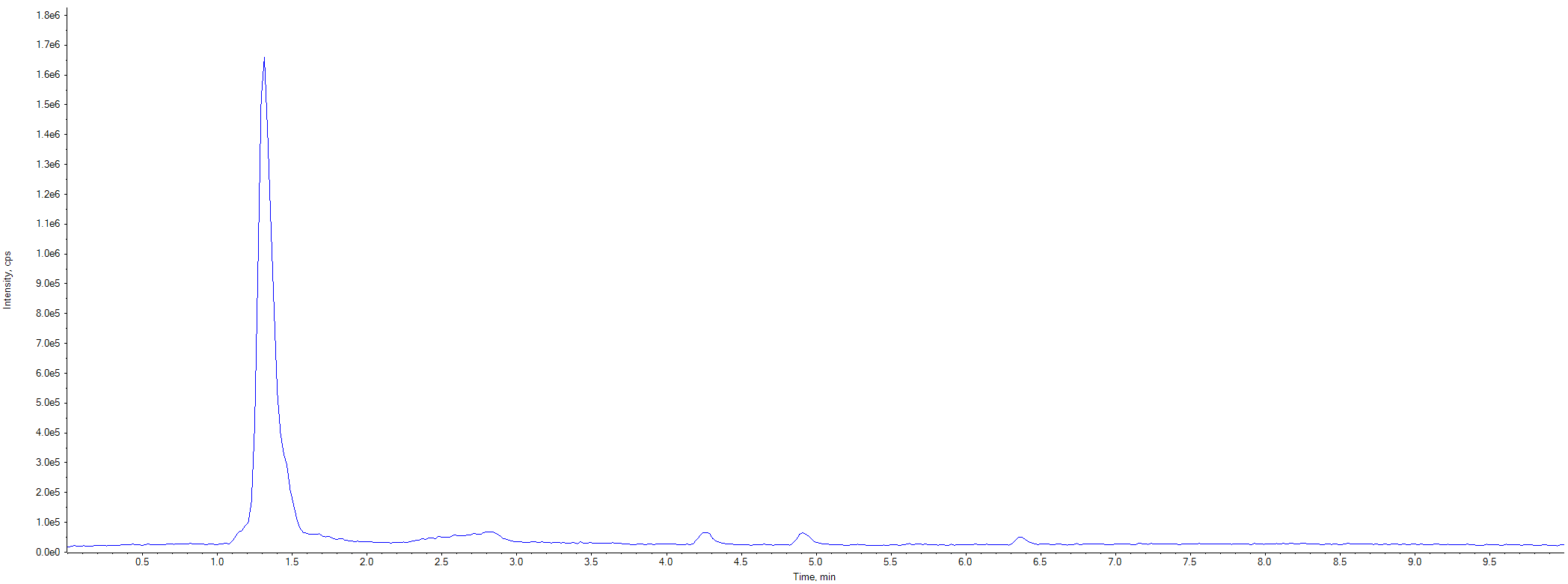


#### **Sample Name:** C_1_100 **Vial #:** 44

####
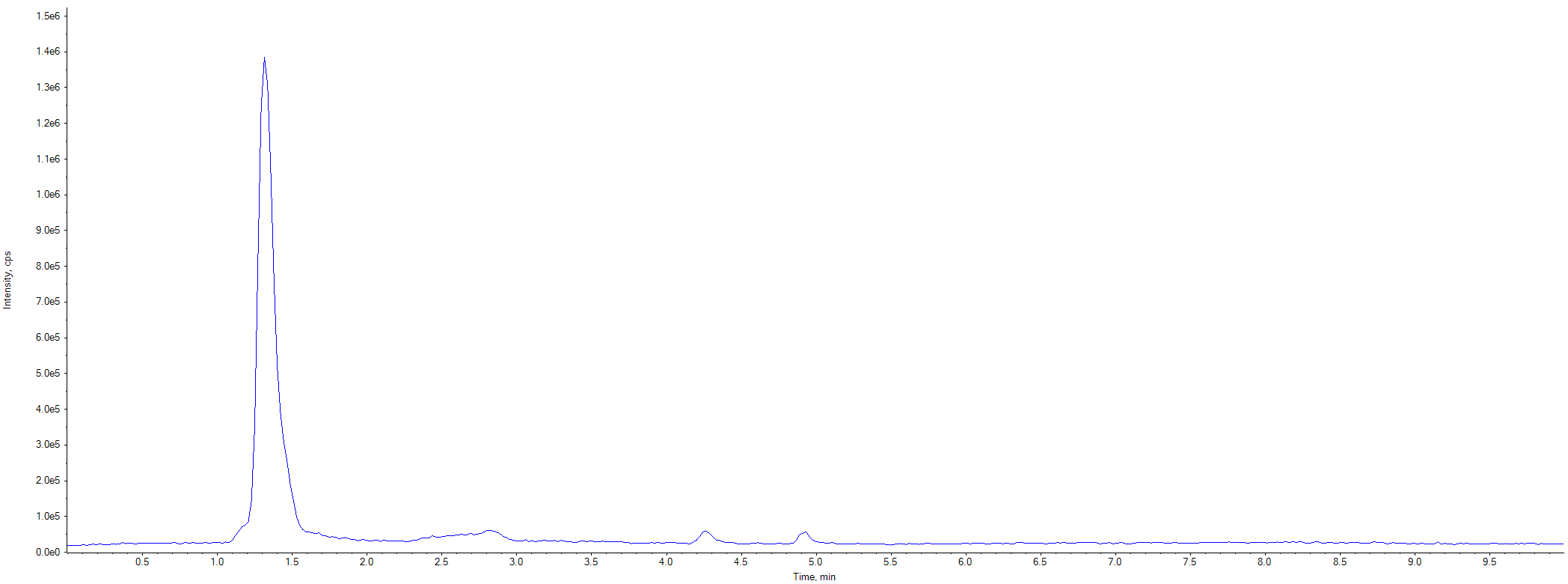


#### **Sample Name:** C_2_100 **Vial #:** 45

####
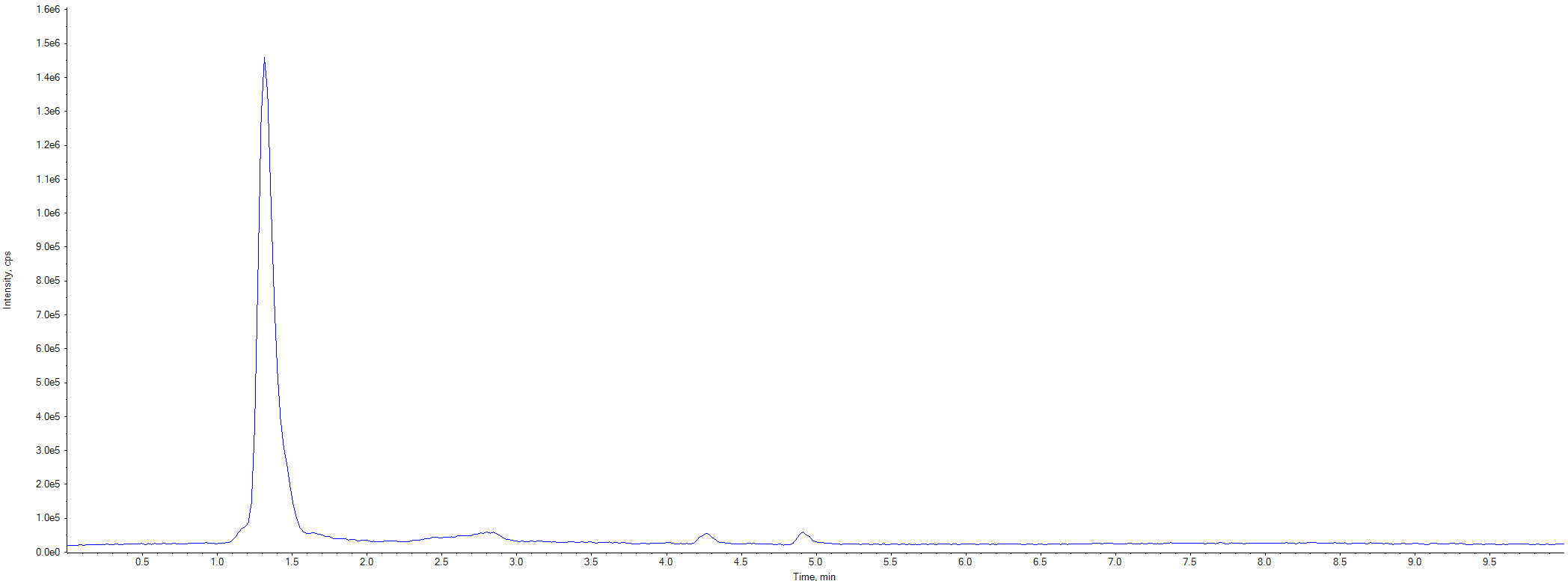


#### **Sample Name:** C_3_100 **Vial #:** 46

####
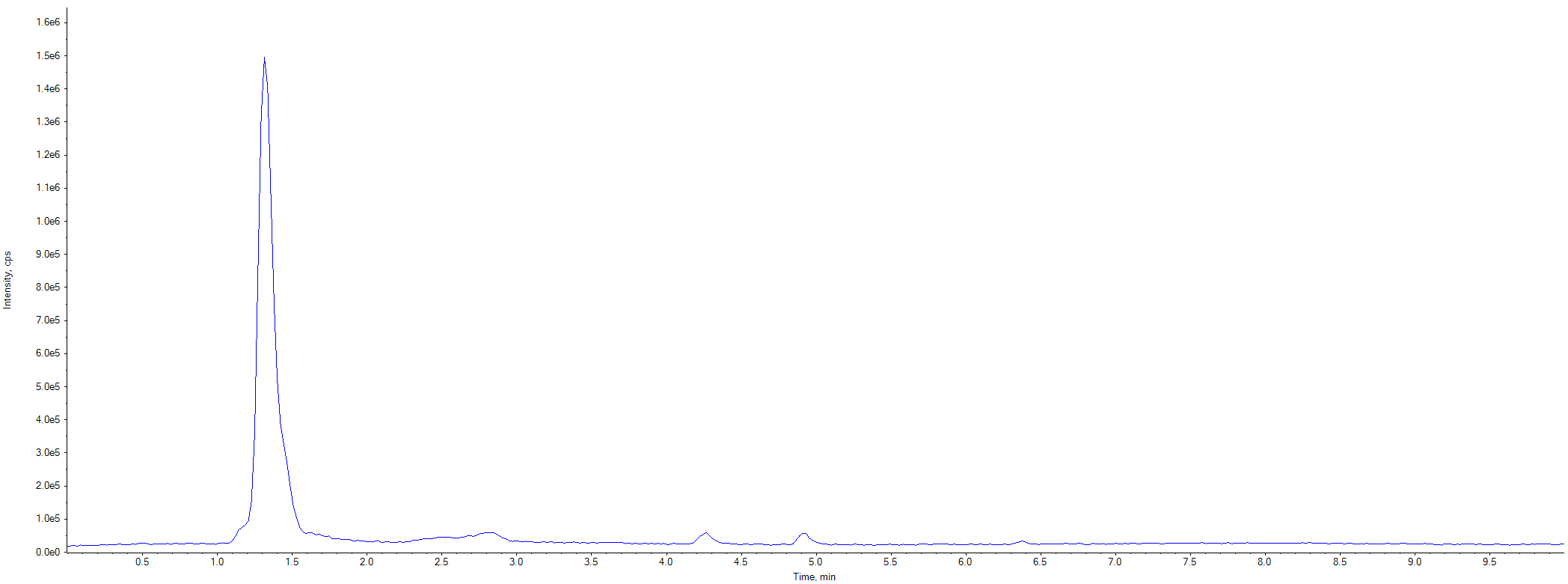


#### **Sample Name:** C_4_100 **Vial #:** 47

####
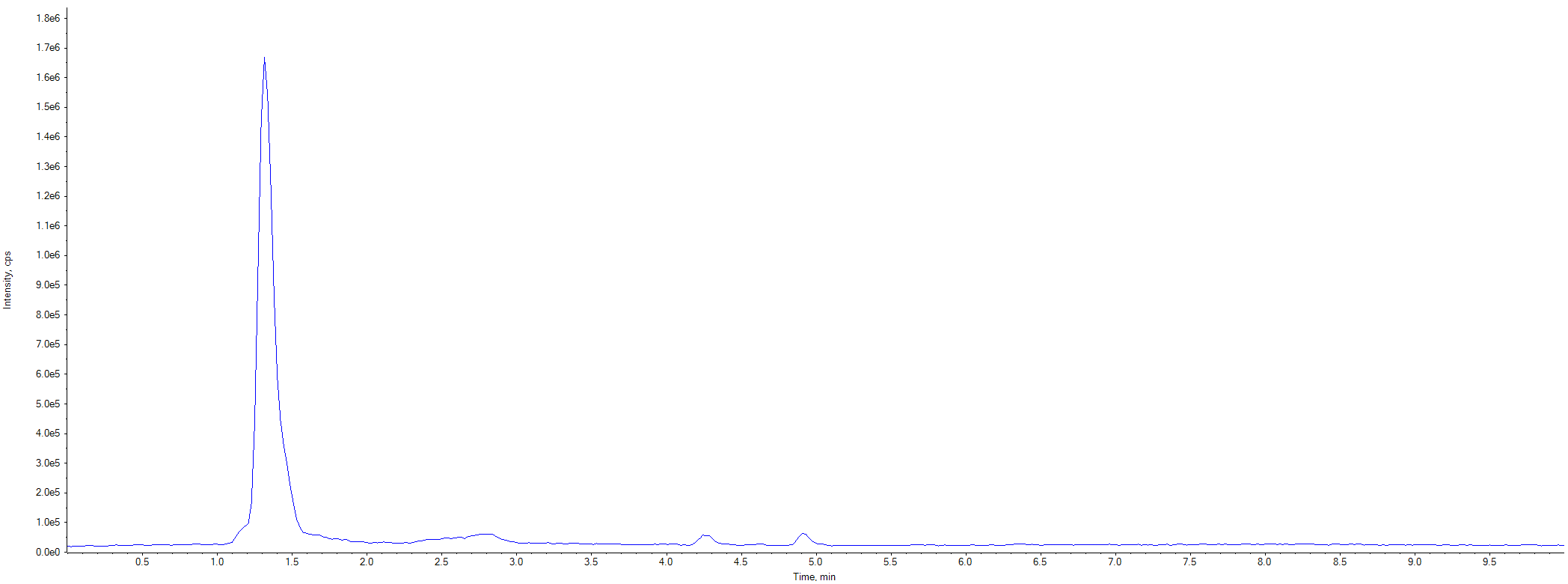


#### **Sample Name:** C_5_100 **Vial #:** 48

####
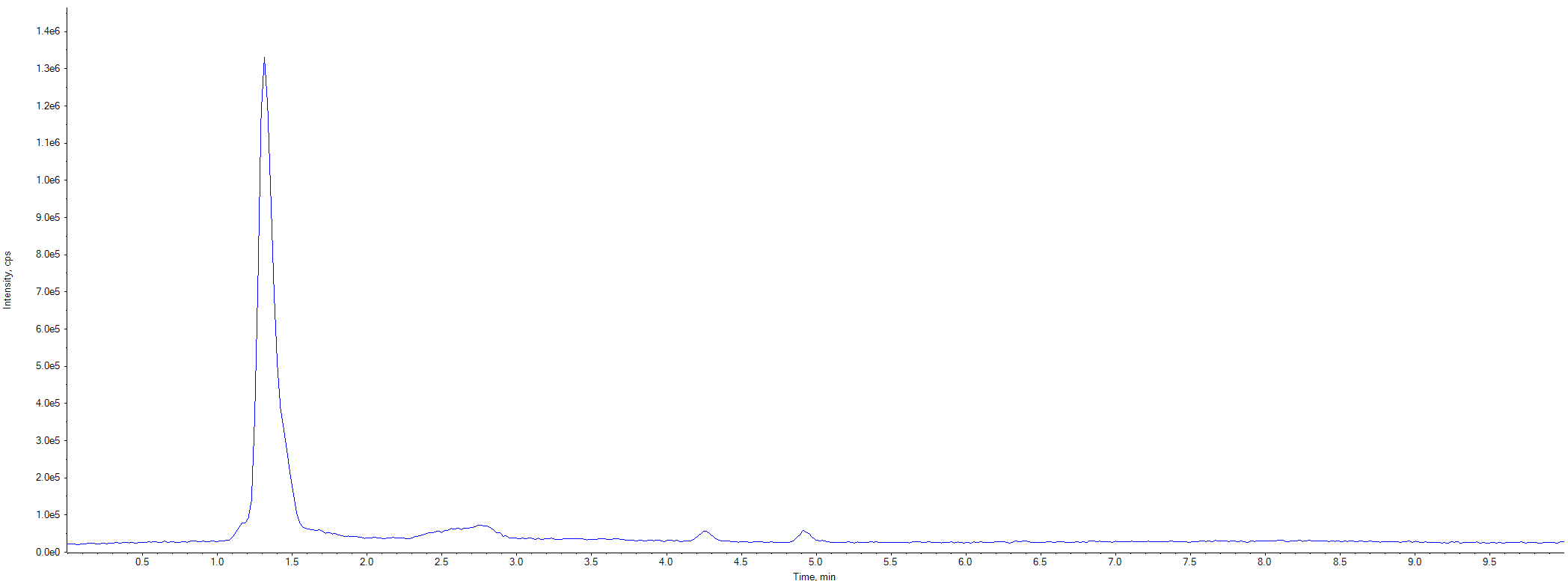


#### **Sample Name:** C_6_100 **Vial #:** 2

####
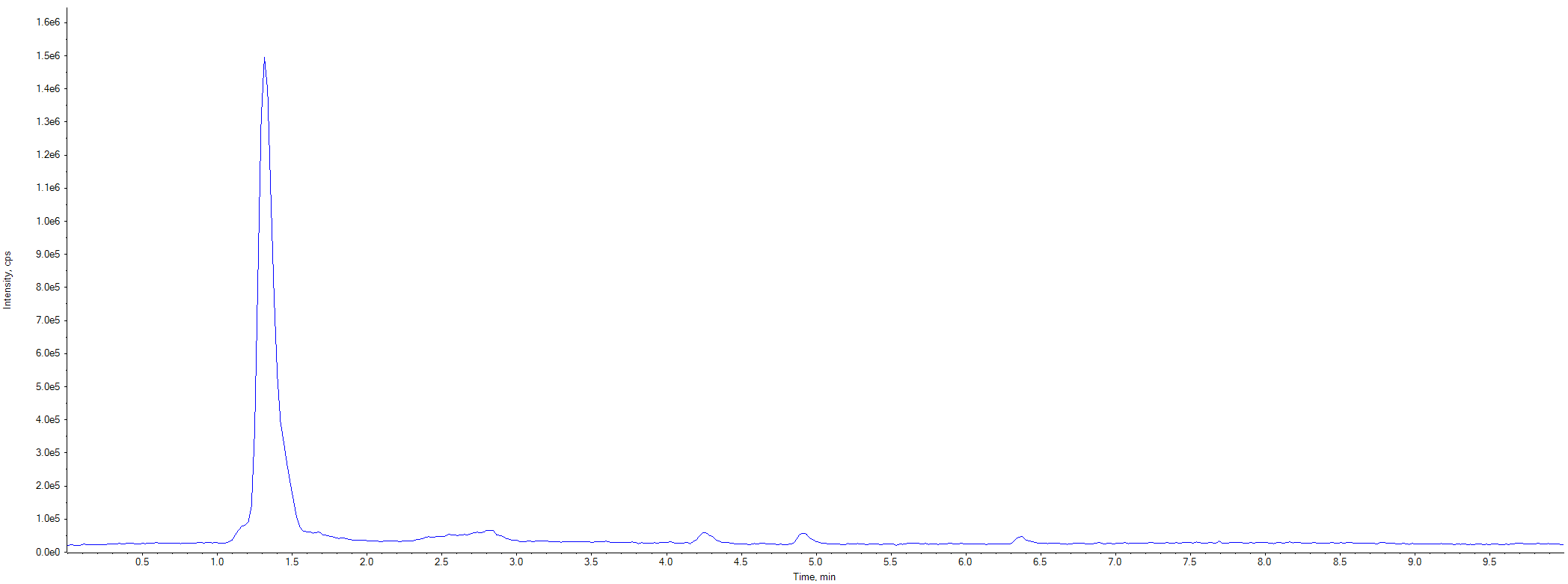


#### **Sample Name:** QC_100 **Vial #:** 31

####
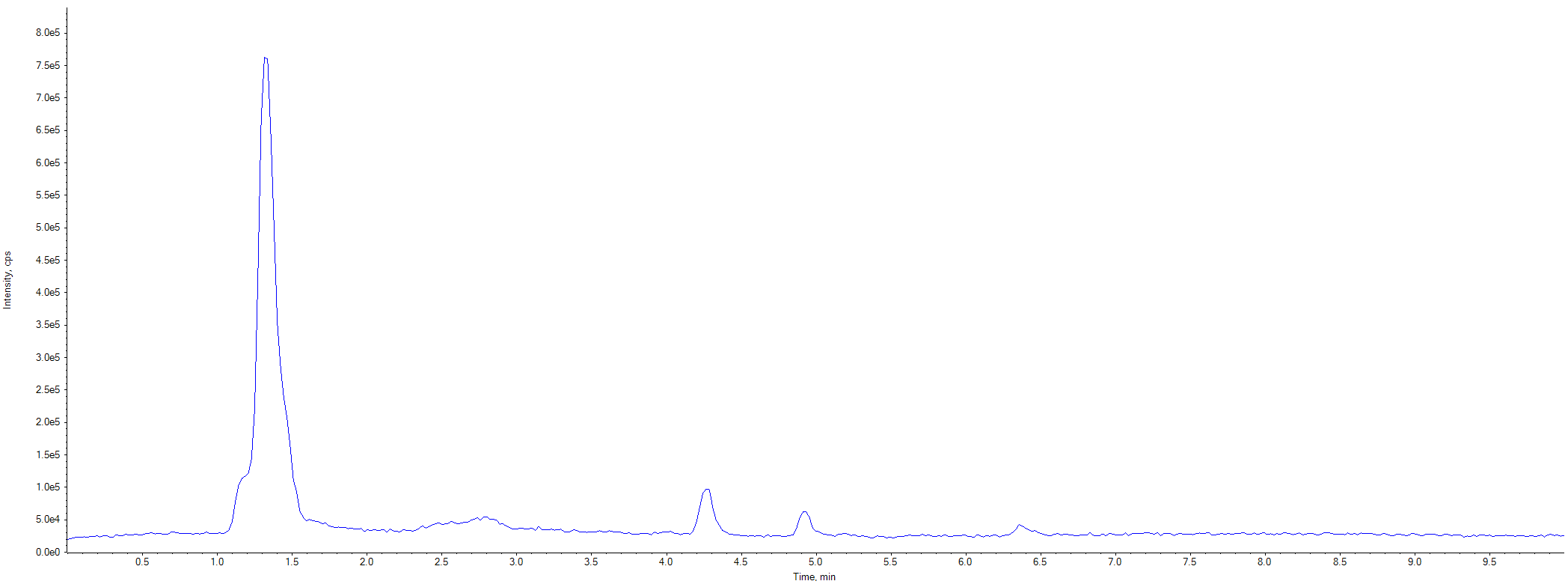


#### **Sample Name:** QC_100 **Vial #:** 31

####
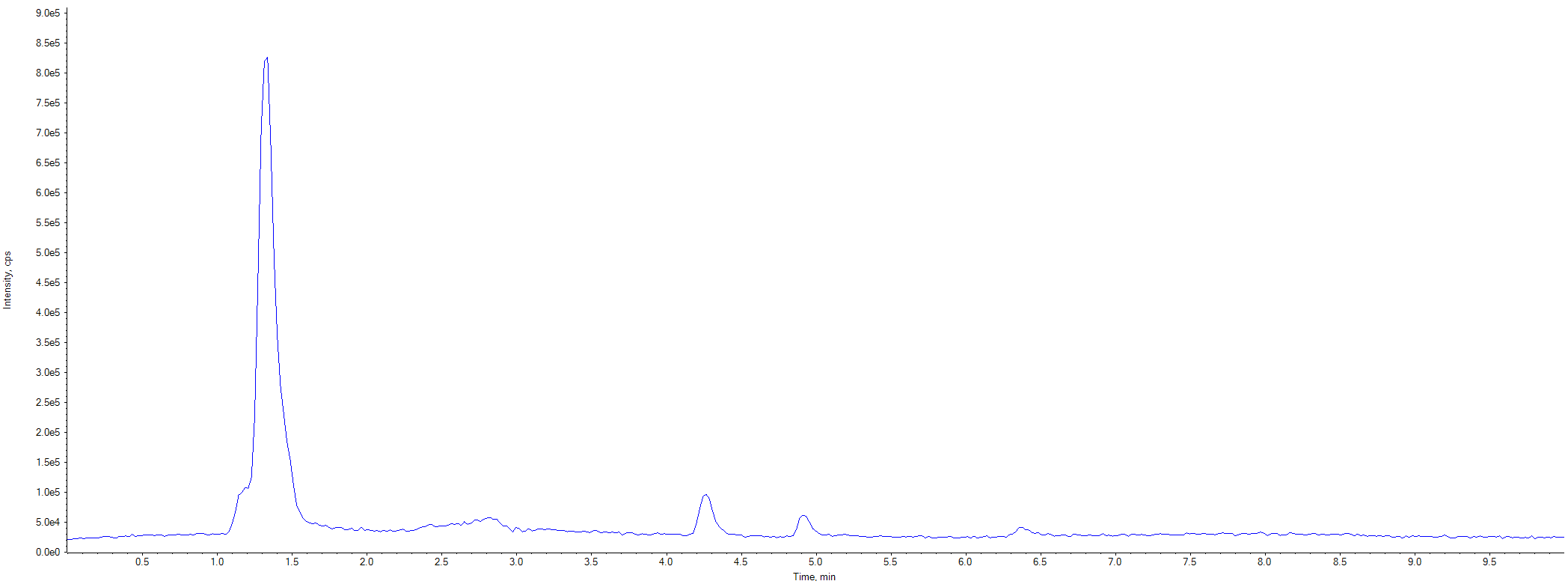


#### **Sample Name:** QC_100 **Vial #:** 31

####
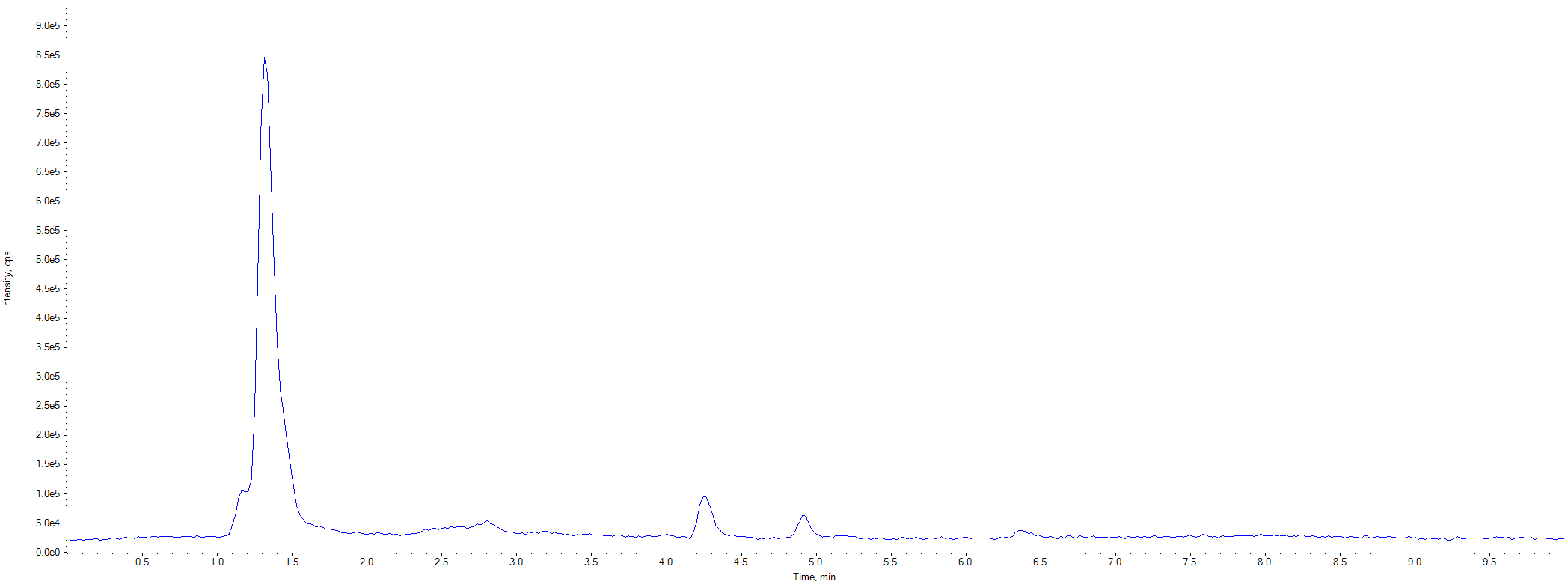


#### **Sample Name:** QC_100 **Vial #:** 31

####
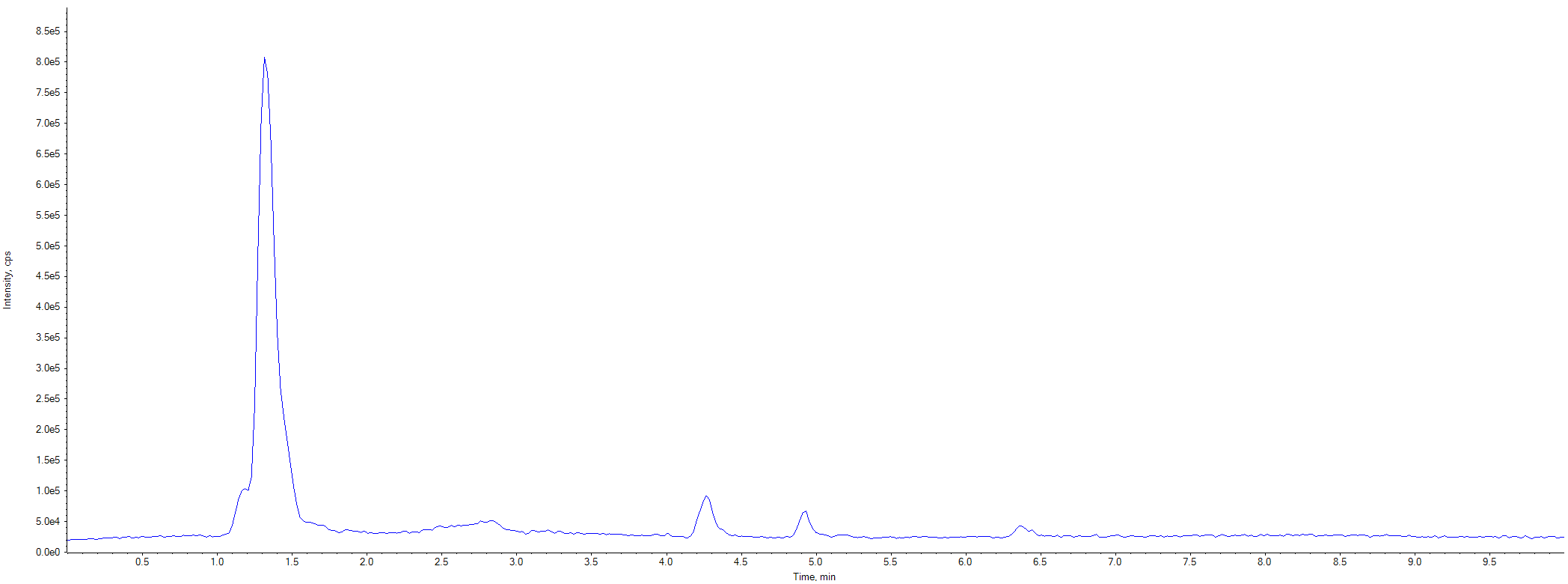

Supplement: Supplementary file 4 [file Table4.docx]
